# Construction of a high-density genetic map and QTL mapping of leaf traits and plant growth in an interspecific F_1_ population of *Catalpa bungei* × *Catalpa duclouxii* Dode

**DOI:** 10.1186/s12870-019-2207-y

**Published:** 2019-12-30

**Authors:** Nan Lu, Miaomiao Zhang, Yao Xiao, Donghua Han, Ying Liu, Yu Zhang, Fei Yi, Tianqing Zhu, Wenjun Ma, Erqin Fan, Guanzheng Qu, Junhui Wang

**Affiliations:** 1 State Key Laboratory of Tree Genetics and Breeding, Key Laboratory of Tree Breeding and Cultivation of State Forestry Administration, Research Institute of Forestry, Chinese Academy of Forestry, Beijing, 100091 People’s Republic of China; 2 College of Landscape Architecture, Nanjing Forestry University, Nanjing, 210037 Jiangsu People’s Republic of China; 3 College of Forestry, Northwest A&F University, Yangling, 712100 Shaanxi People’s Republic of China; 4 State Key Laboratory of Tree Genetics and Breeding, Northeast Forestry University, Harbin, People’s Republic of China

**Keywords:** *Catalpa bungei*, Genetic map, RAD-seq, QTL mapping, Leaf traits, Plant growth

## Abstract

**Background:**

*Catalpa bungei* is an important tree species used for timber in China and widely cultivated for economic and ornamental purposes. A high-density linkage map of *C. bungei* would be an efficient tool not only for identifying key quantitative trait loci (QTLs) that affect important traits, such as plant growth and leaf traits, but also for other genetic studies.

**Results:**

Restriction site-associated DNA sequencing (RAD-seq) was used to identify molecular markers and construct a genetic map. Approximately 280.77 Gb of clean data were obtained after sequencing, and in total, 25,614,295 single nucleotide polymorphisms (SNPs) and 2,871,647 insertions-deletions (InDels) were initially identified in the genomes of 200 individuals of a *C. bungei* (7080) × *Catalpa duclouxii* (16-PJ-3) F_1_ population and their parents. Finally, 9072 SNP and 521 InDel markers that satisfied the requirements for constructing a genetic map were obtained. The integrated genetic map contained 9593 pleomorphic markers in 20 linkage groups and spanned 3151.63 cM, with an average distance between adjacent markers of 0.32 cM. Twenty QTLs for seven leaf traits and 13 QTLs for plant height at five successive time points were identified using our genetic map by inclusive composite interval mapping (ICIM). Q16–60 was identified as a QTL for five leaf traits, and three significant QTLs (Q9–1, Q18–66 and Q18–73) associated with plant growth were detected at least twice. Genome annotation suggested that a cyclin gene participates in leaf trait development, while the growth of *C. bungei* may be influenced by CDC48C and genes associated with phytohormone synthesis.

**Conclusions:**

This is the first genetic map constructed in *C. bungei* and will be a useful tool for further genetic study, molecular marker-assisted breeding and genome assembly.

## Background

*Catalpa bungei* (2n = 2 × =40) is a woody plant belonging to the genus *Catalpa*, family Bignoniaceae [1], and an important ornamental tree species widely used in urban forests in central and northern cities in China due to its beautiful flowers, straight stems and moderate efficiency in particulate matter removal [2, 3]. *C. bungei* is native to China and remains mainly distributed in China. According to the records, people in ancient China started to cultivate and utilize *C. bungei* as early as the Han dynasty (202 BC to 220 AD) [4]. In addition to its value in landscaping, *C. bungei* has wood with excellent mechanical properties and high durability that can resist the corrosion caused by microorganisms and insects. It is usually used to make coffins, musical instruments, boats and other upscale wooden products in ancient China [5–8]. Even today, it is still a popular material for upmarket furniture making and house decorating. To satisfy the vast demand for timber supply and urban landscaping in China, efforts have been made towards *C. bungei* hybridization for economic and ecological purposes [9, 10]. However, the lack of genetic information for the target traits has made the formulation of a highly efficient breeding strategy in *C. bungei* much more difficult than in crops.

As *C. bungei* is a timber and urban forest tree, its growth traits are its most important economic traits, and recent studies have demonstrated that leaf traits such as leaf area (LA), petiole length (PL) and others are associated with its capacity to capture particles from the air [11]. These traits are complex quantitative traits that may be determined by several factors, including cell division and expansion, phenology, and photosynthesis efficiency [12]. An understanding of quantitative trait loci (QTLs) would be beneficial to reveal the genetic architecture of these important traits.

Genetic mapping is one of the main methods used to identify QTLs and genes that regulate complex but important traits, such as plant growth [12], flowering time [13] and abiotic stress resistance [14, 15], in plant breeding. Genetic mapping in forest studies mostly employs F_1_ populations, and because of the long lifespan and large size of trees, building a genetic mapping population usually requires large fields and a long duration of land use, which makes the application of genetic mapping much less common in perennial forest species than in annual crops. Despite the difficulties in population construction, limited studies have demonstrated that linkage mapping is still a powerful tool for dissecting complex quantitative traits in forest species. For example, Xia et al. identified nine QTLs and candidate genes regulating leaf shape using a genetic map constructed from an F_1_ population of *Populus deltoides* × *Populus simonii* Carr [16]. Du et al. revealed the genetic architecture of growth traits in poplars using linkage analysis and association studies [17]. Other traits, such as bud burst timing [18], lignin content [19], reproduction-related traits [20], and fruit-related traits [21, 22], have also been subjected to linkage-based QTL mapping in forest species and other woody plants. According to QTL studies, genetic mapping is still one of the most efficient methods for studying genetic characteristics. In addition, genetic maps are also basis for map-based cloning and other genetic analyses [23, 24].

Genetic maps are constructed according to the linkage relationships between molecular markers in the genome, including random amplified polymorphic DNA (RAPD) [25, 26], amplified fragment length polymorphisms (AFLPs) [26–28], simple sequence repeats (SSRs) [26, 28–30], sequence-related amplified polymorphisms (SRAPs) [31], single nucleotide polymorphisms (SNPs) [32], and insertions-deletions (InDels) [33]. Among these types of markers, SNPs and InDels are considered potential applied molecular markers [34]. Currently, next-generation sequencing (NGS) technology, such as whole-genome resequencing and reduced-representation genome sequencing (RRGS), has facilitated the identification of SNP and InDel markers and the construction of high-density genetic maps with molecular markers throughout the plant genome [35]. Restriction site-associated DNA sequencing (RAD-seq) is an RRGS method and has been effectively applied in high-throughput molecular marker discovery and QTL mapping of important traits in woody plants [36–38]. Consequently, the construction of a *C. bungei* high-density genetic map using RAD-seq will not only aid in the development of markers for genetic studies but also help accelerate the breeding process in *C. bungei*; however, such work has not yet been reported.

In this study, an F_1_ segregating population derived from two *Catalpa* cultivars, namely, *C. bungei* “7080” (female parent) and *Catalpa duclouxii* “16-PJ-3” (male parent), was generated. A high-density genetic map was constructed using RAD technology based on the F_1_ population. Subsequently, we located and analysed QTLs associated with leaf traits using the genetic map. In addition, we also studied QTLs associated with plant height at six time points during the growing season. This is the first genetic map in *C. bungei* and lays a foundation for future genetic studies and marker-assisted selection (MAS) of *C. bungei*.

## Results

### Construction of the genetic map

Nearly 288.75 Gb (288,747,675,610 bp) of raw data containing paired-end reads was generated by Illumina sequencing of the 200 F_1_ progeny and their parents using RAD-sequencing (for offspring individuals) and resequencing (for parents). After data filtering, we obtained 963,326,642 clean reads totalling more than 280.72 Gb of clean data with an average Q30 (%) value of 93.0% and a guanine-cytosine (GC, %) content of 37.0%. For the two parents, approximately 9.90 and 9.97 Gb of resequenced clean data was obtained from 16-PJ-3 and 0708, with resequencing coverage of 10.09× and 10.47×, respectively. For the offspring, an average of approximately 1.30 Gb of clean data (ranging from approximately 0.83 to 2.17 Gb) was obtained (Table 1). The clean reads were aligned to the *C. bungei* genome (Additional file 1). Clean reads aligned to multiple positions or no position in the reference genome were discarded. Consequently, 90.76% clean reads for the female parent and 93.74% clean reads for the male parent were obtained. For F_1_ individuals, an average of 94.79% clean reads were aligned to unique positions in the reference genome. All clean reads aligned to unique positions in the reference genome were kept for subsequent SNP calling and genotype determination.

**Table 1:** Summary of RAD-seq and re-sequence data

	7080	16-PJ-3	Offspring (average)	Total
Clean reads				
AANo. of reads	33,078,367	32,809,373	4,487,195	
GC (%)	35.93	36.81	37.12	37.09
Q30 (%)	93.89	94.24	92.51	92.61
Average depth (×)	10.47	10.09	1.30	
Initial variable sites				
No. of SNPs	11,605,507	13,221,020	1,492,295	22,319,406
No. of InDels	1,307,918	1,428,832	147,808	1,593,074
Markers on the map				
No. of markers	6023	4710	9593	
Average depth (×)	13.70	12.86	13.46	

SNPs and InDels were identified using the filtered clean reads with the Unified Genotyper. A total of 25,614,295 SNPs and 2,871,647 InDels were initially identified in 200 F_1_ individuals (Table 1). After removal of the markers with low quality and shallow sequencing depth, 712,786 polymorphic sites were retained and classified into eight segregation patterns. Then, we removed the SNP markers with abnormal bases, a low calling rate in progeny individuals and significant segregation distortion. Finally, 9593 polymorphic sites (including 9072 SNPs and 521 InDels) with four segregation patterns, namely, “nn × np” (3570), “hk × hk” (1119), “lm × ll” (4883) and “ef × eg” (21), were used for linkage analysis. The read counts of individual alleles at the 9593 polymorphic loci were showed in Additional file 2: Table S1.

Female and male maps were first constructed using the selected markers. In the female map (7080), a total of 6023 polymorphic markers fell into 20 linkage groups (LGs) with a 3440.02 cM total distance and a 0.57 cM average marker interval distance. LG2 was the largest LG with a total distance of 212.84 cM and 179 markers (Table 2). LG18 had 662 markers, the maximum number of markers among the 20 LGs. The average distance ranged from 0.22 (LG12) to 1.81 (LG20) cM. Among the 6003 gaps, no gap was less than 5 cM in length, and the length of the largest gap, which was located in LG2, was approximately 4.53 cM. In the male map, 4710 markers fell into 20 LGs, and the total genetic distance was 3226.28 cM, with an average marker interval distance of 0.73 cM. Among the 20 LGs, LG1 had the maximum number of markers (413), with a 173.48-cM total distance, and LG11 had the longest total distance (198.42 cM), with 119 SNPs. The average marker distance ranged from 0.37 (LG18) to 1.8 (LG10) cM. In the male map (16-PJ-3), the longest gap was 3.35 cM in LG11, and no gap was less than 5 cM in length (Table 3). Subsequently, the male and female maps were merged into an integrated map. The final map spanned 3151.63 cM and contained 9593 markers in 20 LGs. Among the 20 LGs, LG5 and LG14 were the longest and shortest groups, spanning 198.05 cM and 125.5 cM and containing 450 and 441 polymorphic sites, respectively. The average distance between markers was 0.32 cM, with a range from 0.16 cM (LG18) to 1.08 cM (LG10). All gaps were fewer than 5 cM in length, and the longest gap was 4.12 cM in LG2 (Table 3). Detailed information on the markers used for genetic map construction and the distances between adjacent markers are provided in Additional file 3: Table S2.

**Table 2:** The description of total marks and distance for the 20 linkage groups

Linkage groups (LGs)	Total Marker			Total Distance (cM)		
	7080	16-PJ-3	Integrated map	7080	16-PJ-3	Integrated map
1	509	413	844	135.41	173.48	169.88
2	179	134	289	212.84	152.89	196.76
3	315	252	500	207.62	121.42	156.11
4	159	189	306	187.06	161.84	127.77
5	239	280	450	170.10	180.62	198.05
6	204	177	351	181.78	194.96	192.45
7	218	149	331	177.67	136.33	152.19
8	270	125	353	185.33	144.81	129.63
9	177	214	338	203.10	151.75	138.26
10	96	91	165	122.66	161.59	177.98
11	62	119	172	140.22	198.42	153.44
12	559	370	842	121.76	194.03	141.11
13	440	281	672	194.144	155.63	149.51
14	281	209	441	199.82	120.88	125.5
15	381	351	616	197.15	155.91	142.12
16	297	270	519	172.33	188.81	149.22
17	545	259	733	127.61	152.87	138.05
18	662	486	996	171.66	179.08	164.09
19	327	198	455	146.85	160.91	169.17
20	103	143	220	184.91	140.03	180.35
Total	6023	4710	9593	3440.02	3226.28	3151.63

**Table 3:** The description of basic characteristics for the marker distance of 20 linkage groups

Linkage groups (LGs)	Average Distance (cM)			Max Gap (cM)		
	7080	16-PJ-3	Integrated map	7080	16-PJ-3	Integrated map
1	0.27	0.42	0.20	0.88	0.97	1.03
2	1.20	1.15	0.68	4.53	3.32	4.12
3	0.66	0.48	0.31	1.65	1.06	1.3
4	1.18	0.86	0.42	2.90	2.17	1.79
5	0.71	0.65	0.44	2.47	2.00	2.37
6	0.90	1.11	0.55	2.65	2.76	2.74
7	0.82	0.92	0.46	1.93	1.73	1.76
8	0.69	1.17	0.37	3.80	2.43	2.25
9	1.15	0.71	0.41	2.80	2.20	1.87
10	1.29	1.80	1.09	3.02	3.34	3.92
11	2.30	1.68	0.90	4.21	3.56	3.19
12	0.22	0.53	0.17	0.67	1.20	0.81
13	0.44	0.56	0.22	1.48	1.42	1.24
14	0.71	0.58	0.29	1.99	1.33	1.29
15	0.52	0.45	0.23	1.37	1.25	1.05
16	0.58	0.7	0.29	1.32	1.43	1.13
17	0.23	0.59	0.19	0.95	1.30	1.1
18	0.26	0.37	0.16	0.75	1.04	0.81
19	0.45	0.82	0.37	1.63	2.04	1.94
20	1.81	0.99	0.82	4.31	2.02	3.04
Total	0.57	0.68	0.33	4.53	3.56	4.12

Haplotype analysis and heat maps are two effective methods for evaluating the quality of genetic maps [39]. In our study, we constructed haplotype maps of the 20 LGs with polymorphic markers to reflect the recombination events of each offspring. The haplotype maps indicated that the missing marker ratio in the genetic map was 0.19%, suggesting high quality (Additional file 4: Figure S1 and Additional file 5: Table S3). Heat maps can reflect the recombination relationships between all the markers in the same LG. Heat maps of 20 LGs indicated that the adjacent markers in the linkage groups were strongly linked and became gradually less linked with increasing distance, suggesting a correct order of the markers in most LGs (Additional file 6: Figure S2). In addition, for most LGs, the Spearman correlation coefficient between the genetic and physical locations was 0.99, with an average physical coverage of 99%, suggesting a relatively high level of genetic collinearity (Additional file 7: Table S4).

### Analysis of leaf and growth traits

**Fig. 1:**
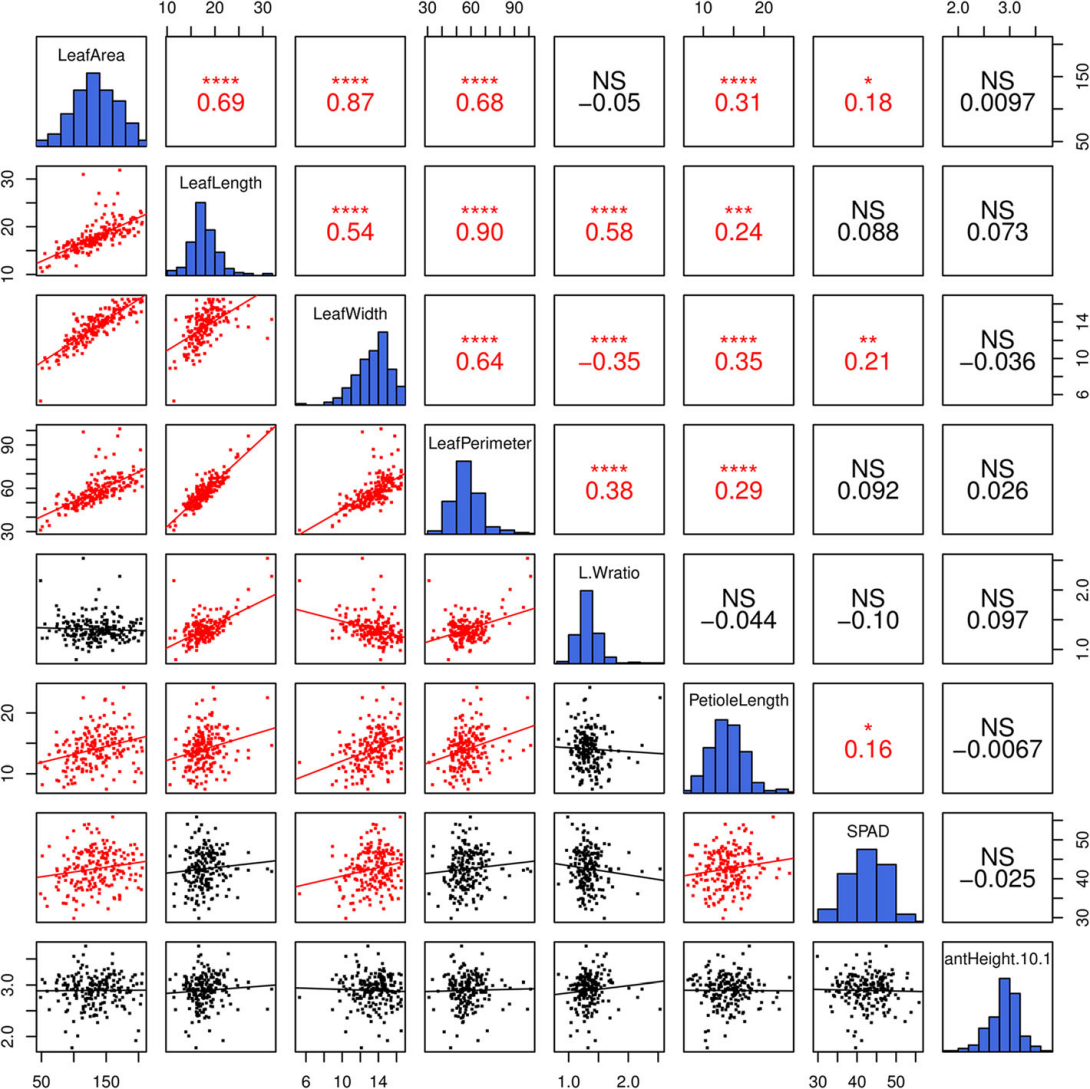
Scatter plots (lower triangle) and correlations (upper triangles) among seven leaf traits and plant height (10.10) in the F_1_ mapping population

A wide range of variation in the seven leaf traits and plant height at six time points was observed (Table 4). The leaves of “7080” were larger and wider than those of “16-PJ-3”, but the remaining five leaf traits did not show significant differences between the parents. In addition, “7080” grew faster than “16-PJ-3” at all six time points, which may be partly due to the higher efficiency of nitrogen utilization and distribution in the photosynthetic system, according to our previous study [10]. The frequency distribution analysis (Fig. 1) indicated that most of the phenotypic values were generally normally distributed, which suggested that the phenotypic data could be used for further QTL analysis. The coefficient of variation (CV, %) in the seven leaf traits ranged from 11.18 to 25.61%. The leaf perimeter (LP) and leaf length (LL) had similar CVs: 17.93 and 17.03%, respectively. Plant height varied from 1.78 to 3.75 m on the 10th of October, and the CV at the six time points ranged from 10.02 to 14.42%. The plant heights on the 31st of July, 15th of August, 31st of August and 10th of October had very similar CVs (10.07, 10.15, 10.02 and 10.20%, respectively), which suggested only minor differences between these growth time points.

**Table 4:** The phenotypic information of *C. bungei* “7080”, *C. duclouxii* “16-PJ-3” and their F_1_ Population

Traits	Male (mean)	Female (mean)	F_1_ Population				
			Mean ± SD	Min	Max	Repeatability	CV (%)
LA (cm^2^)^a^	127.99	170.63	133.41 ± 34.17	48.69	205.70	0.89	25.61
LL (cm)	17.98	19.59	17.84 ± 3.03	10.61	31.86	0.87	17.03
LW (cm)^a^	12.68	15.06	13.43 ± 1.82	5.29	16.51	0.92	13.57
LP (cm)	52.83	63.79	57.57 ± 10.32	30.98	101.19	0.90	17.93
L/W	1.41	1.30	1.34 ± 0.20	0.83	2.53	0.86	15.41
PL (cm)	13.88	13.98	14.03 ± 2.97	7.46	24.22	0.94	21.19
SPAD	43.50	42.98	42.55 ± 4.76	29.84	55.88	0.91	11.18
Plant height (m)							
6/30^a^	1.07	1.42	1.28 ± 0.18	0.77	1.84	–	14.42
7/15^a^	1.60	2.01	1.85 ± 0.23	1.11	2.51	–	12.85
7/31^a^	1.92	2.42	2.22 ± 0.22	1.44	2.92	–	10.07
8/15^a^	2.36	3.02	2.72 ± 0.27	1.70	3.54	–	10.15
8/31^a^	2.48	3.06	2.85 ± 0.28	1.75	3.66	–	10.02
10/10^a^	2.55	3.21	2.89 ± 0.29	1.78	3.75	0.92	10.20

“^a^” indicates a significant difference of the measured traits between male and female parent (one-way ANOVA, *P* < 0.05, α = 0.05)

Correlation analysis of the seven leaf traits and plant height (10.10) showed that leaf traits have no correlations with plant height, suggesting that the two types of traits may develop independently of each other (Fig. 1). Similarly, the SPAD readings had no correlations with the other six leaf traits, which implied that the chlorophyll content of leaves may not significantly influence their development. LA exhibited strong positive correlations with leaf width (LW) (0.87) and moderate positive correlations with LL (0.69) and LP (0.68) and weak positive correlations with PL (0.31) but no correlations with L/W. Four leaf traits, LA, LL, LW and LP, had certain positive correlations with each other; PL had weak positive correlations with LA (0.31), LL (0.24), LW (0.35) and LP (0.29), suggesting a possible minor association between the development of petioles and leaves. In addition, no significant negative correlations were found, except for the L/W ratio and LW (−0.35).

### QTL mapping of leaf and growth traits

A total of 33 QTLs for seven leaf traits and plant height at five time points were successfully identified using the integrated genetic map and the phenotypic data (Additional file 8: Figure S3 and Additional file 9: Figure S4). Twenty QTLs for leaf traits, including six LA associations, five LL associations, one LW association, one LP association, one L/W ratio association, four PL associations and two SPAD value associations, explained 2.33 to 16.51% of the phenotypic variation. Two QTLs, Q3–172 (LA) and Q17–84 (SPAD), exhibited over-dominance, while six QTLs, Q16–60 (LA), Q16–67 (LA), Q16–60 (LL), Q16–60 (LP), Q16–60 (L/W) and Q16–60 (PL), showed partial dominance. Among the 20 QTLs for leaf traits, Q16–60, Q16–67 and Q16–97 were mapped to chromosome 16, and Q16–60, which was identified for LA, LL, LP, the L/W ratio and PL, explained 5.16, 16.51, 14.0, and 6.21% of the phenotypic variation in LA, LL, LP and L/W with LOD scores of 3.28, 17.64, 5.47 and 4.27, respectively, and the high phenotypic variance explained (PVE) of LA and LL at Q16–60 suggested that this site may be highly associated with these two traits. In addition, four QTLs were detected on chromosome 19, three of which (Q19–106, Q19–116 and Q19–126) were associated with LA and another of which (Q19–137) was associated with SPAD value.

We also detected 13 QTLs for plant height at five time points, except for time point 7.31. The QTLs explained 5.81 (Q18–60) to 9.02% (Q18–73) of the phenotypic variation. Eight QTL sites (13 associations) were mapped to LG3 (1), 9 (1), 16 (2), 18 (2) and 19 (1), and eight QTLs were detected at more than one time point: Q9–1 (at time points 6.30 and 7.15), Q18–66 (at time points 8.15 and 8.31) and Q18–73 (at time points 6.30, 7.15, 8.15 and 8.31). Three of the 13 QTLs, Q9–1 (6.30), Q9–1 (7.15) and Q19–59 (7.15), showed over-dominance, and Q16–56 (8.15) showed partial dominance. Detailed information on the QTLs has been provided in Additional file 10: Table S5 and Additional file 11: Table S6.

### Candidate gene prediction in a subset of QTLs

**Fig. 2:**
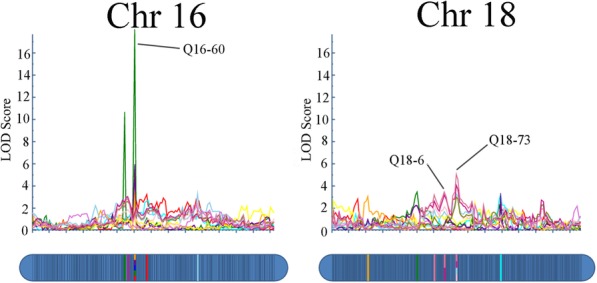
LOD profile for QTL associated with five leaf traits: LA (red), LL (green), LP (blue), L/W (purple), PL (yellow) at Q16–60; Plant growth traits: 8/15(rosy), 8/31(pink) at Q18–66 and 6/30(light pink), 7/15(dusty blue), 8/15(rosy), 8/31(pink), at Q18–73

To further test the accuracy and usability of our genetic map, the leaf trait QTL Q16–60 and plant height QTLs Q18–66 and Q18–73 were used for gene prediction because they were identified at more than one time point or for more than one leaf trait (Fig. 2, Additional file 12: Table S7). Q18–66 was found as a QTL for plant height (8.15) and plant height (8.31). Similarly, Q18–73 was found as a QTL for plant height (6.30), plant height (7.15), plant height (8.15) and plant height (8.31). Although Q9–1 was also identified as a QTL at more than two time points (plant height 6.30 and 7.15), no genes were found between marker sca9_231994 and sca9_261968 in the physical map. The QTL region of Q16–60 was 0.45 cM in length, exhibited a physical distance of approximately 58 Kb and contained 5 putative predicted genes in the reference genome of *C. bungei*. Four of these genes were annotated in the Gene Ontology (GO) database, but none were identified in the Kyoto Encyclopedia of Genes and Genomes (KEGG) pathway database. GO annotation suggested that the genes were involved in DNA binding (evm.model.group5.473), cyclin regulation (evm.model.group5.471), and embryo development (evm.model.group5.475), among other processes.

The QTL regions of Q18–66 and Q18–73 were mapped to 0.320 and 0.359 cM, respectively, and their physical distances were approximately 213 Kb and 52 bp in the reference genome. The 52-bp sequence of Q18–73 was located in the coding region of one putative gene, evm.model.group7.1784, which may encode a cleavage and polyadenylation specificity factor 3 (CPSF) subunit 3-II isoform X3 according to its annotation. GO annotation suggested that this gene mainly participates in catalytic activity, mRNA processing, polar nucleus fusion, and protein binding, among other processes. Fifteen putative predicted genes were found in Q18–66. Except for one unknown gene, GO annotation suggested that the remaining 14 genes in Q18–66 mainly participate in flavonoid biosynthesis, DNA binding, and magnesium ion binding, among other processes. KEGG annotation suggested that two genes, namely, evm.model.group7.1478 and evm.model.group7.1479, mainly participate in glycolysis, carbon metabolism, and biosynthesis of amino acids, among other processes, suggesting possible functions in plant growth. Other genes in Q18–66 may be involved in anthocyanin biosynthesis, alpha-linolenic acid metabolism, aminobenzoate degradation, and the mRNA surveillance pathway, among others.

## Discussion

### Using the RAD-seq strategy to identify molecular markers

Genetic maps have been used as an important tool to assist in plant breeding and to elucidate the genetic architecture of complex quantitative traits of interest to breeders. Genetic maps with a high marker density are essential for marker discovery and precise QTL location. RRGS methods, such as RAD-seq, genotyping-by-sequencing (GBS) and specific locus amplified fragment (SLAF) sequencing, have strongly facilitated molecular marker identification. For example, by using 3868 SNPs identified with GBS, Zhang et al. constructed a high-density genetic map of tree peony (*Paeonia suffruticosa* Andr.) with a much longer total genetic distance (13,175.5 cM) and shorter average marker interval distance (3.40 cM) than the genetic map containing 35 SSR markers with a 9.70 cM average marker interval and 338.2 cM total genetic distance obtained by Guo et al. for the same mapping population [40, 41]. A similar outcome was observed for genetic maps of groundnut, in which SSR-based genetic maps contained approximately 135 to 191 markers and an RRGS-based map contained 1685 SNPs [42]. RAD-seq has proven to be an effective method for identifying large numbers of polymorphic sites in plants. For example, in a report on *Vitis* plant genetic map construction, RAD-seq identified 8,481,484 SNPs and 1,646,131 InDels in the parents and 176 F_1_ plants, 65,299 and 4832 of which, respectively, were used to construct a high-density genetic map spanning 3014 cM, with an average coverage of 99.83% and with 99.99% of gaps fewer than 5 cM in length [35]. Using the same method, Guo et al. constructed a genetic map of peach (*Prunus persica*) that included 1310 SNPs spanning 454.2 cM with an average marker distance of 0.347 cM [43]. This map was of much higher quality than maps constructed with SSRs or other markers [44, 45] because the former included many more markers and was of a larger scale. Although the process of RAD library construction is more complicated than double digest-RAD (dd-RAD), SLAF and GBS, longer genome fragments can be obtained for other studies [46]. In total, 260.85 Gb RAD reads with an average length of nearly 290 bp were obtained from the 200 progeny, and these data could also be used for other studies. The GC content of the RAD sequences was approximately 37.11%, which is slightly different from that of the reference genome (34.12%); this difference may be due to the selection of restriction enzymes. We initially found 22,319,406 SNPs and 1,563,074 InDels according to the RAD sequences. For the SNPs, approximately 66.85% were transition-type, which is similar to the results for *Taxodium distichum* (64.52%) [12], *Juglans regia* (68.32%) [47], *Corchorus capsularis* (69.07%) [48] and other species. In addition, of the initial variants, more than 50% SNPs showed polymorphic between the parents, which suggested a high genetic diversity of *C. bungei* and provided various genetic and genomic information for further studies*.*

### Analysis of the genetic map

We constructed a genetic map using an F_1_ population, which is usually used for genetic map construction in forest trees due to their long life cycle, of *C. bungei* × *C. duclouxii*. The final genetic map contained 9593 polymorphic markers and 20 LGs with an average of nearly 500 markers on each LG. Compared with the genetic maps of other woody species constructed using the RRGS strategy, the number of mapped markers was greater than those of *Ziziphus jujube* [49]*, Juglans regia* [47] and *Paeonia suffruticosa* [50], but less than those of *Taxodium distichum* [12], *Vitis* [35], and *Actinidia chinensis* [51]*.* The smaller number of mapped markers compared to a few previous studies may be partly due to the smaller depth of sequencing in the offspring individuals in our study, which may have led to the elimination of markers that did not satisfy the sequencing depth threshold. To further optimize our genetic map, more markers will be added in the future. The total genetic distance of the genetic map was 3151.63 cM, with a 0.32 cM average interval distance. Moreover, all gaps (distances between adjacent markers) were less than 5 cM, suggesting a high density. Increasing the number of individuals in the mapping population could effectively improve the resolution of the genetic map by detecting the homologous recombination of chromosomes with lower recombination rates during meiosis [12]. Our results suggested that a mapping population with 200 individuals may be enough for the construction of a high-density genetic map. The markers on the 20 LGs were not evenly distributed in our study: a maximum of 996 markers was found on LG 18 and a minimum of 165 markers on LG 10. More than half of the LGs (11) contained fewer than 500 markers (fewer than the average level), which may indicate a lack of genetic information on these LGs. RRGS strategies using double enzyme digestion, such as dd-RAD and SLAF, could obtain markers that are more uniformly distributed, and these techniques could be considered in later studies.

### QTL analysis of leaf and growth traits

Concerning leaf and growth trait variation, we found high repeatability for all measured traits. The repeatability ranged from 0.86 (L/W) to 0.94 (PL). Our results suggested that the phenotypic stability of the offspring clones was high. The high-quality genetic map developed in our study allowed for high-resolution QTL mapping, and a total of 16 QTLs for seven leaf traits were mapped, including Q16–60, which was identified as a QTL for LA, LL, LP, the L/W ratio and PL, for a total of five traits. According to the correlation analysis, most of the six leaf traits (LA, LL, LW, LP, L/W ratio and PL) were correlated with each other; however, no QTL associated with all six leaf traits was identified. Similar results can also be found in the QTL mapping of leaf or growth traits in other species [12, 52, 53]. This may be because these traits are complex quantitative characters that are controlled by multiple QTLs, and it is not easy to precisely identify all the QTLs for the target traits. Moreover, several previous studies have found that the results of QTL mapping for flag leaf size in crops could be influenced by the environment, which suggested QTL mapping in different environments could be used to enhance accuracy, and in fact, this strategy has been tried in other studies [54, 55]. In a recent study, Xia et al. mapped 42 QTLs for nine leaf traits in an F_1_ poplar population at three time points, and 9 of these QTLs were found at two or more time points. This repeatability at different time points suggests high QTL mapping confidence [16]. However, in our study, only one time point was mapped; in a future study, QTLs for leaf traits will be mapped at multiple time points and locations to enhance QTL mapping accuracy. Leaf size is controlled by the interconnection between cell division and cell expansion [56]. According to a previous study, changing the expression of cyclin B1;1 and other cell cycle-related genes significantly influences the cell division rate of leaves [57, 58], and a moderate increase in *CYCD3* expression in *Arabidopsis* increases the cell number and LA [59, 60]. In addition, cyclin genes also influence leaf flatness and erectness [61, 62]. In our study, a possible cyclin gene (evm.model.group5.471) was found in Q16–60, which implied that cyclin-mediated cell division may participate in the formation of leaf traits.

In recent years, examination of the dynamic QTLs for plant growth characteristics in a continuous set of time points has been performed; for example, Du et al. mapped the growth traits of poplar at 12 successive time points and found that some QTLs were specific to one time point, while some QTLs were found at several continuous or discontinuous time points, which may be due to their specific functions in plant growth at different growth stages [63]. In the present study, QTLs for plant height at five time points were mapped, and 8 QTLs were detected at more than one time point for a cumulative PVE of 61.4%. Thus, these loci were further examined to analyse the candidate genes. In our study, the inclusive composite interval mapping (ICIM) method was used to map the QTLs associated with leaf traits and plant height. Yang et al. mapped QTLs of growth traits of *Taxodium ‘Zhongshansha’* and identified 5 common QTLs by the CIM and ICIM methods to ensure the reliability of the QTLs. In their study, the number of QTLs detected by the CIM method was only half that identified by the ICIM method, which may have been due to the higher detection power of the ICIM method [12]. Similarly, Dodia et al. used the CIM and ICIM additive (ICIM-ADD) methods to map QTLs for stem rot disease resistance and plant architecture in groundnut and obtained different results: the QTLs obtained with the CIM method explained more phenotypic variance [42]. Improper mapping methods may lead to false results, and different methods may influence QTL mapping results; for this reason, using more mapping methods may be an effective strategy for improving QTL mapping accuracy [12], and more mapping methods should be used to precisely identify QTLs.

By using QTL mapping, we identified two genomic regions of 213.76 Kb and 52 bp on Chr18 that contained QTLs for plant height. The 213.76-Kb genomic region with the flanking markers sca18_15484674 and sca18_15698439 (0.32 cM in the genetic map) had a PVE of ~ 6.6%. A total of 15 genes were identified in this region according to the genome annotation. The gene evm.model.group7.1464 located in this region is an orthologue of *AT3G01610.1* in the genome of *Arabidopsis thaliana. AT3G01610.1* encodes the cell division control protein 48 homologue C-like (CDC48C), which is involved in cell division, cell expansion, and cell differentiation, among other processes [64]. CDC48 is reportedly involved in plant growth: Bae et al. suppressed the expression of the CDC48 homologue gene in tobacco by virus-induced gene silencing, and antisense RNA interference caused not only severe growth cessation in the shoot and leaf but also flower sterility [65]. Another gene located in this region, evm.model.group7.1477, is an orthologue of *AT1G76680.2* in *A. thaliana*, which encodes a 12-oxophytodienoate reductase that participates in jasmonic acid (JA) biosynthesis [66, 67]. Although a high level of JA inhibits the growth of plant stems [68], low concentrations of JA can promote cell expansion and shoot elongation [69, 70], which indicates that endogenous JA synthesis may influence plant height in our mapping population; however, this possibility requires further verification. Additionally, we found an *AT5G04660.1* orthologue, namely, evm.model.group7.1470, which may encode a cytochrome P450 family member. Several cytochrome P450 family members have been shown to participate in plant growth. For example, *EUI1*, a cytochrome P450 monooxygenase cloned in rice, regulates internode elongation by modulating the gibberellin response [71, 72]. In addition, cytochrome P450 members also participate in brassinosteroid [73] and indole-3-acetic acid [74] biosynthesis and influence cell elongation or plant height [75, 76]. The 52-bp sequence in Q18–73 was located in evm.model.group7.1784 in the physical map, which may encode a possible cleavage and polyadenylation specificity factor 73 (CPSF73) homologue subunit. CPSF73 is an endonuclease that participates in small nuclear RNA (snRNA) 3′-end processing and plays roles in flower and embryo development [77]. However, it is not clear whether it influences plant height.

To increase the precision of candidate gene prediction, transcriptomics or quantitative real-time PCR analyses can be used to identify RNA variants and the expression of genes within the mapped QTL regions. Other omics analyses, such as metabolomics, can also provide some information on the chemical compounds related to phenotypic variations. In future studies, these analyses will be used to further verify the candidate genes in our study.

## Conclusion

A high-density genetic map of *C. bungei* × *C. duclouxii* was constructed using the RAD-seq strategy, with the help of which 20 and 13 QTLs were identified that were associated with leaf and growth traits, respectively, which explained moderate phenotypic variation. Our study has laid a foundation for molecular marker-assisted breeding in *C. bungei*. Moreover, the genome sequences we obtained have enriched the resources for the public to study the evolution and functional genomics of *C. bungei*. The candidate genes identified within the QTLs may be promising genes for regulating leaf traits and increasing plant growth in *C. bungei* and will be further studied.

## Methods

### Mapping population and DNA extraction

*C. bungei* “7080” (female parent, entire leaf, Additional file 13: Figure S5) is an excellent clone selected by Luoyang Academy of Agriculture and Forestry, Luoyang, Henan Province and we have got the permission to applicate “7080” as breeding material from Luoyang Academy of Agriculture and Forestry and cultivated this *C. bungei* clone in an artificial forest belonging to Research Institute of Forestry, Chinese Academy of Forestry in Luoyang (34.71°N, 112.54°E) in the year 2006. *C. duclouxii* Dode “16-PJ-3” (male parent, lobed leaf, Additional file 13: Figure S5) is a wild individual grown in Panjiang town, Guizhou, China (25.75°N, 103.83°E). In the year 2016, Dr. Wenjun Ma collected the pollen and shoots of “16-PJ-3” (the collection of “16-PJ-3” did not need any necessary permission after we consulting to the relevant department) and carried out the hybridization. Finally, a total of 681 F_1_ progenies were obtained by crossing “7080” and “16-PJ-3”. The seeds of F_1_ progeny were sown and grown into seedlings in the greenhouse of Chinese Academy of Forestry in 2017. In the year 2018, 200 randomly selected F_1_ individuals and the two parents were asexually propagated and planted in the experimental field of Luoyang Academy of Agricultural and Forestry Science (Luoyang, China, N 112.55°, E 34.71°). A randomized block design was applied, with two ramets per clone in each plot and 5 replicates. All the plant material collections in our study were complied with national guidelines. The field experiment we made were in accordance with local legislation. The voucher specimens were deposited in Research Institute of Forestry, Chinese Academy of Forestry. Dr. Wenjun Ma and Dr. Junhui Wang undertook the formal identification of the samples.

Young and healthy leaf samples (second or third leaves from the apex) of both parents and 200 F_1_ individuals were collected in June 2018. All leaf samples were frozen in liquid nitrogen immediately and stored at −80 °C. We extracted genomic DNA using a modified cetyltrimethylammonium bromide (CTAB) method [78, 79]. To eliminate residual RNA, all extracted DNA samples were treated with RNase (Takara, Shuzo, Otsu, Japan). Finally, DNA concentration and purity were determined by a NanoDrop 2000 UV-vis spectrophotometer and checked on 1% agarose gels.

### RAD library construction and high-throughput sequencing

We prepared the 200 RAD libraries according to the RAD protocol [80] with minor modification. Briefly, 200 ng of qualified genomic DNA from each sample (200 F_1_ individuals) was fully digested by 20 U of restriction endonuclease *EcoR*I (New England Biolabs, Ipswich, MA, USA) at 37 °C in a 50 μl reaction mixture based on an evaluation of the reference genome and the result of a DNA digestion pre-experiment (Additional file 14: Figure S6). After digestion, barcoded P1 adapters were ligated to the *EcoR*I restriction site for each sample individually. Then, all the samples were sheared to an average size of 500 bp using a Bioruptor (Diagenode, Liège, Belgium). DNA fragments 350 to 450 bp in size were collected using 2% agarose gel for library construction. Thereafter, the fragments were blunt end repaired, and a 3′ adenine overhang was added to the sequences. Finally, a P2 adapter containing unique Illumina barcodes (San Diego, CA, USA) was added to each library. All libraries were amplified using PCR with high-fidelity thermostable DNA polymerase (New England Biolabs, Ipswich, MA, USA) and purified before sequencing. The resequencing libraries of the two parents, “7080” and “16-PJ-3”, were constructed at the same time. The RAD libraries and two resequencing libraries were sequenced using the Illumina HiSeq X Ten platform using 150-bp paired-end reads by Shanghai Major Biological Medicine Technology Co., Ltd.

### SNP and InDel calling and genetic map construction

To ensure read quality for later analysis, all raw reads were filtered using Trimmomatic [81] to discard low-quality reads (quality score below 30), reads with more than 10% unidentified nucleotides and reads aligned to the adapter. Next, the clean data were analysed using a standard SNP and InDel calling pipeline. Briefly, all the clean reads were first mapped to the *C. bungei* reference genome using Burrows-Wheeler Aligner (BWA) software [82] with the setting of “mem -t 4-k 32-M”. To avoid false mapping results, only reads with a unique mapping position in the genome were sorted using SAMtools [83]. Subsequently, variants were called and filtered using the Genome Analysis Toolkit (GATK) unified [84] using standard filtering parameters according to the GATK Best Practices pipeline [85]. Then, the variants were more precisely filtered based on the following three strict criteria: (1) a mapping quality less than 37; (2) a quality depth less than 24; and (3) a sequence depth less than 10-fold (in parents) or 3-fold (in offspring). The screened variant markers were further divided into eight segregation patterns: “ab×cd” (four alleles), “nn × np” (two alleles and one parent heterozygous), “hk × hk” (two alleles and double heterozygous), “ef × eg” (three alleles and double heterozygous), “cc × ab” (three alleles and maternal homozygous), “aa×bb” (two alleles and double homozygous), “ab×cc” (three alleles and parental homozygous) and “lm × ll” (two alleles and maternal heterozygous). Finally, unqualified molecular markers were further removed based on the following three strict criteria: (1) abnormal bases; (2) a variant call rate (missing rate) less than 70%; and (3) significant segregation distortion (chi-square test, *P* value<0.05). The SNP calling parameters were determined after we overall considering the results of several parameter combinations to guarantee the us enough credible markers for later study (Additional file 15: Table S8).

Because an F_1_ population was used in our study, markers with the segregation patterns “ab×cd”, “ab×cc”, “cc × ab”, “ef × eg”, “nn × np”, “hk × hk”, and “lm × ll” were selected to construct the genetic map. All filtered markers were first divided into 20 groups according to their physical locations on the same chromosome, and the markers were then ordered using MSTmap software [86]. Next, the SMOOTH algorithm [87] was used to correct the genotyping errors or deletions according to the relationship between the ordered markers. The genetic distance between markers was calculated using the Kosambi mapping function. Furthermore, a haplotype map and heat map analysis were used to evaluate the quality of the genetic map.

### Phenotyping of leaf traits and dynamic plant height

The leaf trait parameters in the “7080 × 16-PJ-3” F_1_ population were measured on 2018/9/5. We chose the 3rd whorl of fully expanded leaves below the apex to detect the leaf traits (according to our previous study, the 3rd whorl of fully expanded leaves of *C. bungei* were mature functional leaves, and their characters were stable at the collection time chosen for our study). The chlorophyll content was measured five times at different positions on the surface of each leaf using a SPAD-502 Plus chlorophyll meter (Konica Minolta Holdings, Inc., Chiyoda-ku, Tokyo, Japan), and the average value was calculated to represent the chlorophyll content. After we measured the chlorophyll content, the leaves were harvested and scanned by a CI-203 Portable Laser Leaf Area Meter (CID Inc., Washington, USA) for a total of five leaf parameters: leaf length (LL), leaf width (LW), the leaf length/width (L/W) ratio, leaf area (LA) and leaf perimeter (LP). The petiole length (PL) was measured using a ruler.

The development of trees is a complex dynamic process that is regulated by both gene networks and the environment. A traditional mapping strategy using phenotypic data measured at only one time point may not exactly reflect the genetic control of developmental processes [88], so growth data at multiple time points were used to map QTLs. According to our previous observations, the growth of *C. bungei* in Luoyang usually started at the end of April, proceeded rapidly in early July, and finished at the end of September. The height of all plants was measured on 2018/6/30 (before the rapid growth period), 2018/7/15 (during the rapid growth period), 2018/7/31 (during the rapid growth period), 2018/8/15 (during the rapid growth period), 2018/8/31 (the end of the rapid growth period), and 2018/10/10 (the end of the growing season), for a total of six time points, which included the entire rapid growth period (from July to August), in a growth season.

We had two plants of each clone in each block, and the average values of all parameters in each block were calculated from the individual seedlings of the same clone and used as the trait data. The five blocks of plants were measured as five replicates. The correlations between different traits and frequency distribution of the F_1_ population were calculated using R software with the Pearson method [89]. The repeatability of plant height and leaf traits was calculated using the R ASReml package [90]. The phenotypic data were analysed by a one-way ANOVA to discriminate the seven leaf traits and six growth traits between “7080” and “16-PJ-3” using SPSS version 19.0 (SPSS Inc., Chicago, IL, USA).

### QTL mapping and candidate gene selection

Quantitative trait locus (QTL) analysis was conducted using GACD software [91] with the inclusive composite interval mapping (ICIM) method [92]. A significant logarithm of odds (LOD) threshold was determined using 1000 permutation tests (*P* < 0.05) for all traits. Finally, because the average LOD significance thresholds of both the seven leaf traits and the six growth traits were 3.0, a LOD significance threshold value of 3.0 with a 95% confidence interval was determined for the traits [63]. The potential locations of the QTLs were described according to their LOD peak locations and their surrounding regions. Additive (*a*) and dominance (*d*) effects were calculated based on the formulation of Muchero [93], according to the computed results by GACD, which has been introduced in detail by Nzuki [94]. The QTL mode of action was calculated as the ratio of dominance to the absolute additive value (*d*/|*a*|), where *d*/|*a*| ratios larger than 1 were regarded as over-dominance; ratios between 0 and 1 were regarded as partial dominance and ratios less than 1 were regarded as under-dominance [95]. Information about the candidate genes in the mapped QTL regions was obtained according to the annotation of the reference genome.

## Supplementary information

**Additional file 1. **Brief information of *C. bungei* reference genome.

**Additional file 2: Table S1.** The read counts of individual alleles (including both parents and F1 alleles) at the 9,593 polymorphic loci.

**Additional file 3: Table S2.** Information for molecular markers and their locations in each LG of “7080”, “16-PJ-3” and the integrated map.

**Additional file 4: Figure S1.** Haplotype maps of the integrated genetic map. Blue and green represent markers originating from the female (7080) and male (16-PJ-3) parent, respectively. Grey represents the “hk×hk” markers, and white represents missing data. Vertical axis indicates the markers in the LG, and horizontal axis indicates the individuals.

**Additional file 5: Table S3.** Percentages of missing data in each LG.

**Additional file 6: Figure S2.** Heat maps of the integrated genetic map. Each cell represents the pairwise LOD scores of two markers.

**Additional file 7: Table S4.** Description of correlation coefficients between the genetic and physical positions of 20 LGs in the integrated genetic map.

**Additional file 8: Figure S3.** QTL analysis of the seven leaf traits using the ICIM method in GACD. The x-axis indicates the map position (cM) in the 20 LGs, while the y-axis represents the LOD score. The horizontal line in the chart represents the LOD threshold.

**Additional file 9: Figure S4.** QTL analysis of the plant growth traits at different time points using the ICIM method in GACD. The x-axis indicates the map position (cM) in the 20 LGs, while the y-axis represents the LOD score. The horizontal line in the chart is the LOD threshold.

**Additional file 10: Table S5.** Summary of QTLs for leaf traits mapped by ICIM.

**Additional file 11: Table S6.** Summary of QTLs for plant growth mapped by ICIM.

**Additional file 12: Table S7.** The protein sequences and annotation of genes identified in Q16-60, Q18-66 and Q18-73.

**Additional file 13: Figure S5.** The leaf of “7080” (A) and “16-PJ-3” (B).

**Additional file 14: Figure S6.** The gel electrophoresis of DNA digestion pre-experiment using *Taq*I, *EcoR*I and *Mse*I. The “OS” stands for original DNA sample without enzyme treatment; Marker 1 and marker 2 are 1Kb DNA Ladder (TransGen Biotech, Beijing, China) and Marker I (Tiangen, Beijing, China), respectively.

**Additional file 15: Table S8.** The number of variants we obtained using different selecting parameter combinations.

## Data Availability

The clean data of 200 offspring individuals and parents has being uploaded to the NCBI SRA database (http://www.ncbi.nlm.nih.gov/sra), with the accession number: PRJNA551333, Other data that supporting the conclusions of the article have been uploaded as additional files.

## Abbreviations

CIM: Composite interval mapping;
CTAB: Cetyltrimethylammonium bromide
GBS: Genotyping by sequencing
ICIM: Inclusive composite interval mapping
InDel: Insertion-deletion
L/W: Leaf length/leaf width
LA: Leaf area
LG: Linkage group
LL: Leaf length
LOD: Logarithm of odds
LP: Leaf perimeter
LW: Leaf width
MAS: Marker-assisted selection
NGS: Next-generation sequencing
PL: Petiole length
QTL: Quantitative trait loci
RAD-seq: Restriction-site associated DNA sequencing
RRGS: Reduced-representation genome sequencing
SLAF: Specific-locus amplified fragment sequencing
SNPs: Single nucleotide polymorphisms
SPAD: Soil and plant analyzer development
