# Supplementary material for: Construction of a high-density genetic map and QTL mapping of leaf traits and plant growth in an interspecific F1 population of Catalpa bungei × Catalpa duclouxii Dode
Source: BMC Plant Biol. 2019 Dec 30;19:596. doi: 10.1186/s12870-019-2207-y (PMC6937828; doi:10.1186/s12870-019-2207-y)
Supplement: Supplementary file 1 — Additional file 1. Brief information of C. bungei reference genome. [file 12870_2019_2207_MOESM1_ESM.docx]

The *Catalpa bungei* referance genome were sequenced by combing Illumina short reads (69.74 Gb), SMRT long reads using PacBio platforms (80.85 Gb). BioNano Irys optical map (120.14 Gb) and 10×genomics (136.14 Gb) data were used to assemble the sequences. Finally, Hi-C data (74.64Gb) were used to sort majority of sequences into 20 pseudochromosomes. High quality genes were annotated with the assistance of transcriptome data from different organs. For some brief assembled and annotated information, please see the table below:

| Categories |  |
| --- | --- |
| Estimate of genome size (by k-mer) | 763.41Mb |
| Total size of assembled scaffold | 738.10 Mb |
| Number of scaffolds | 151 |
| N50 (contig) | 1.68 Mb |
| N50 (scaffold) | 40.30 Mb |
| Heterozygosity | 1.45% |
| Anchored scaffold size | 695.11Mb |
| Number of gene models | 26,465 |
| Number of annotated genes | 25,478 |
