# Supplementary material for: Construction of a high-density genetic map and QTL mapping of leaf traits and plant growth in an interspecific F1 population of Catalpa bungei × Catalpa duclouxii Dode
Source: BMC Plant Biol. 2019 Dec 30;19:596. doi: 10.1186/s12870-019-2207-y (PMC6937828; doi:10.1186/s12870-019-2207-y)
Supplement: Supplementary file 4 — Additional file 4: Figure S1. Haplotype maps of the integrated genetic map. Blue and green represent markers originating from the female (7080) and male (16-PJ-3) parent, respectively. Grey represents the “hk×hk” markers, and white represents missing data. Vertical axis indicates the markers in the LG, and horizontal axis indicates the individuals. [file 12870_2019_2207_MOESM4_ESM.pdf]

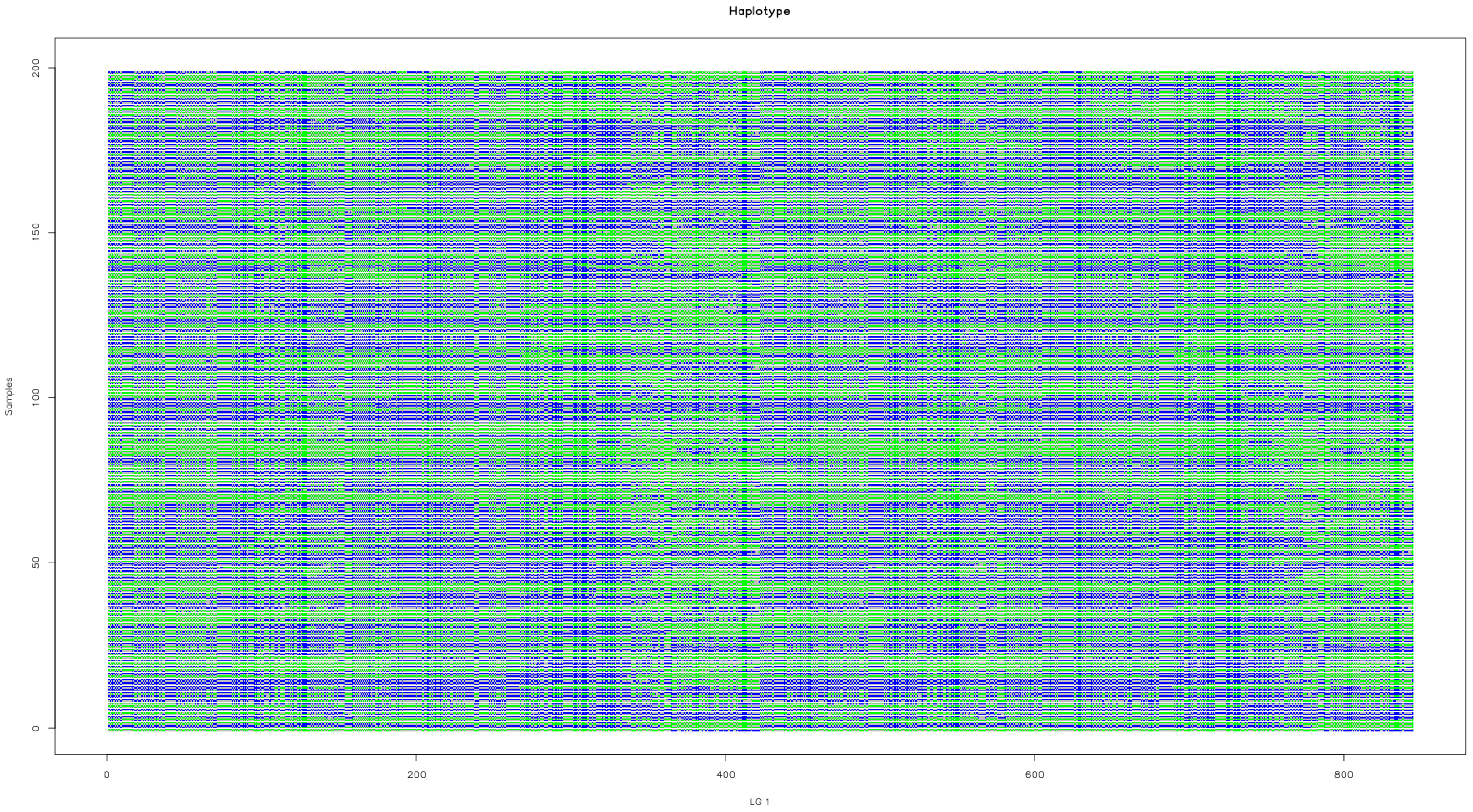
Haplotype
Samples
LG 1
0
50
100
150
200
0
200
400
600
800

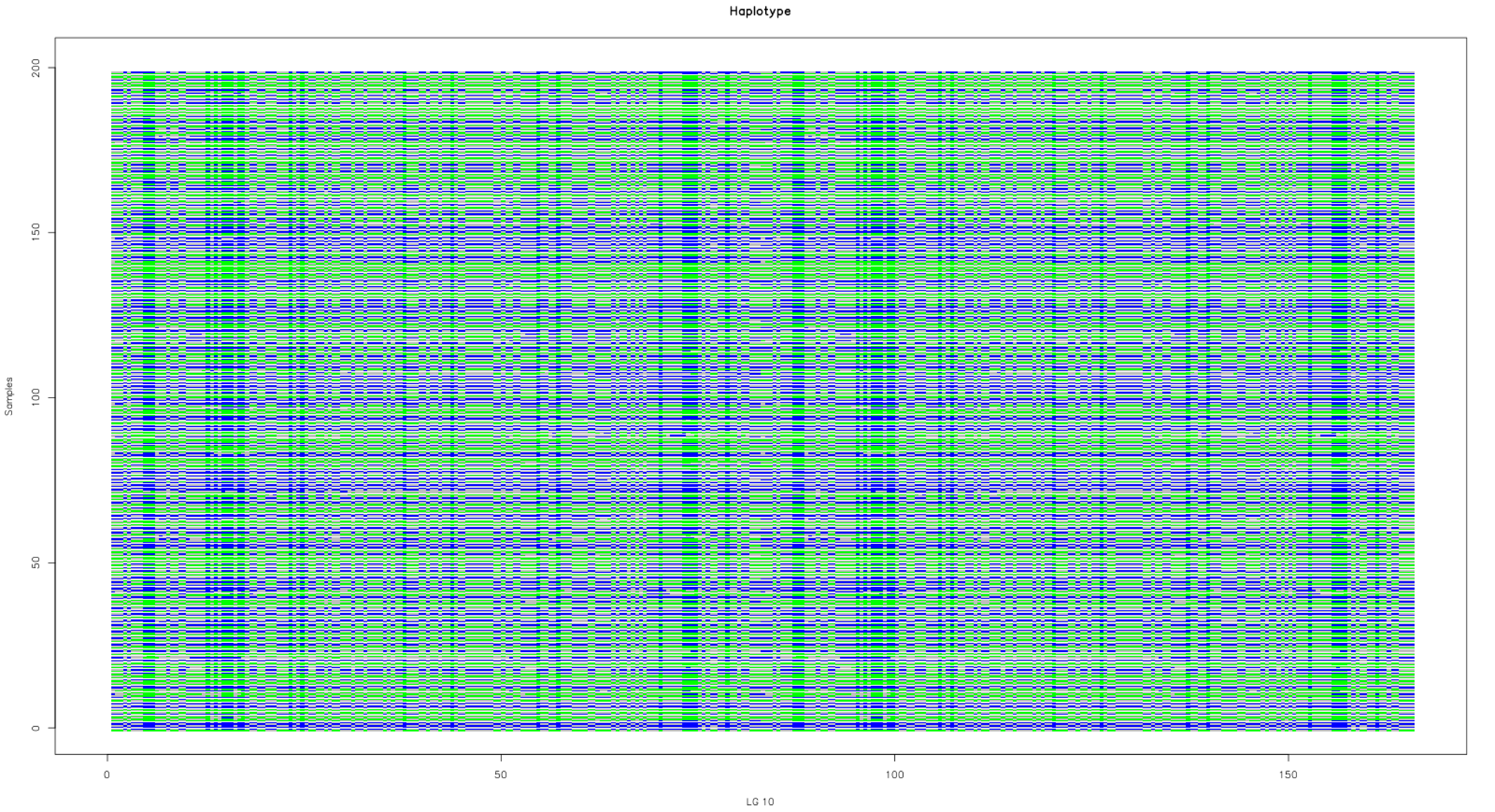
Haplotype
Samples
0
50
100
150
200
LG 10

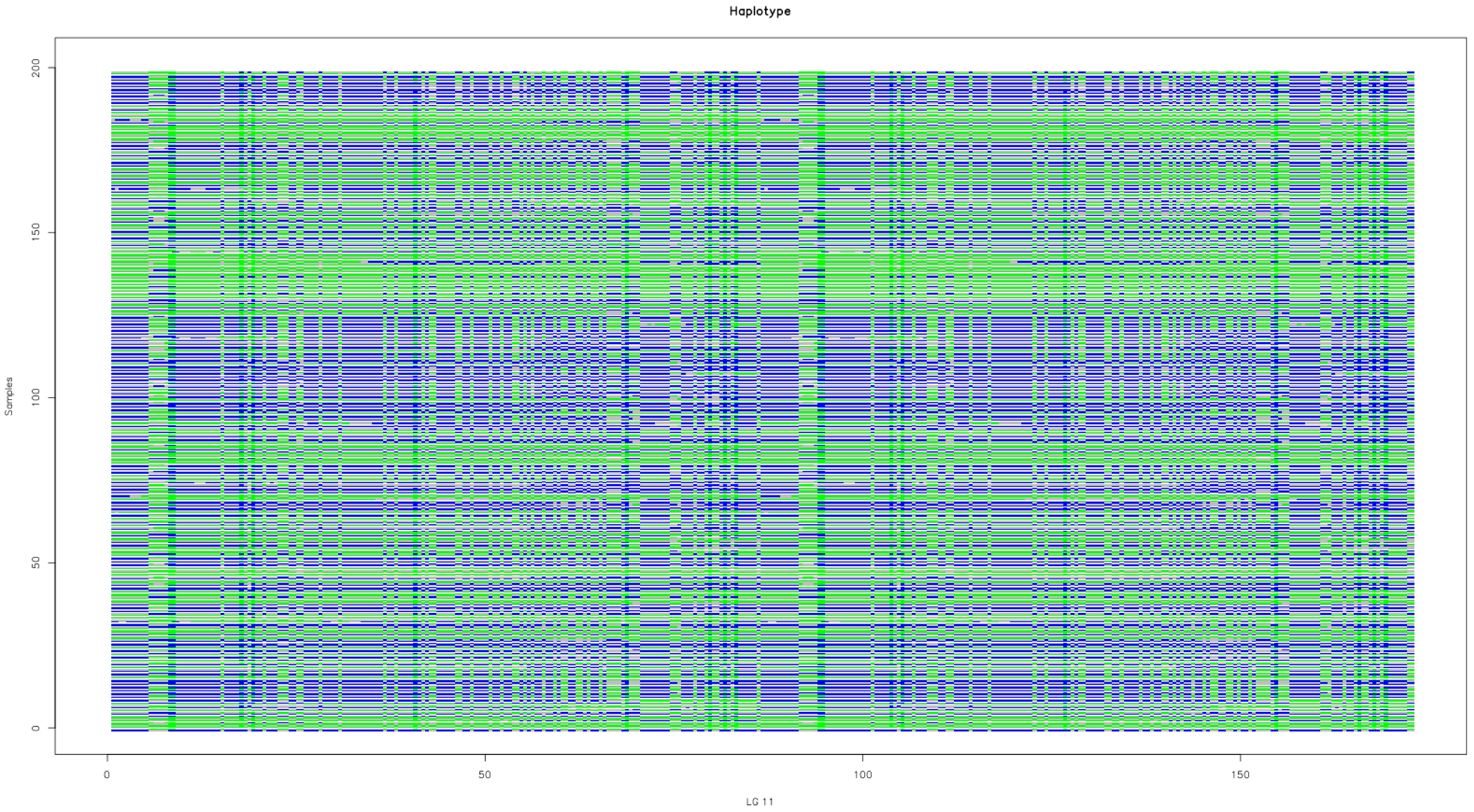
Haplotype
Samples
0
50
100
150
200
LG 11
0
50
100
150

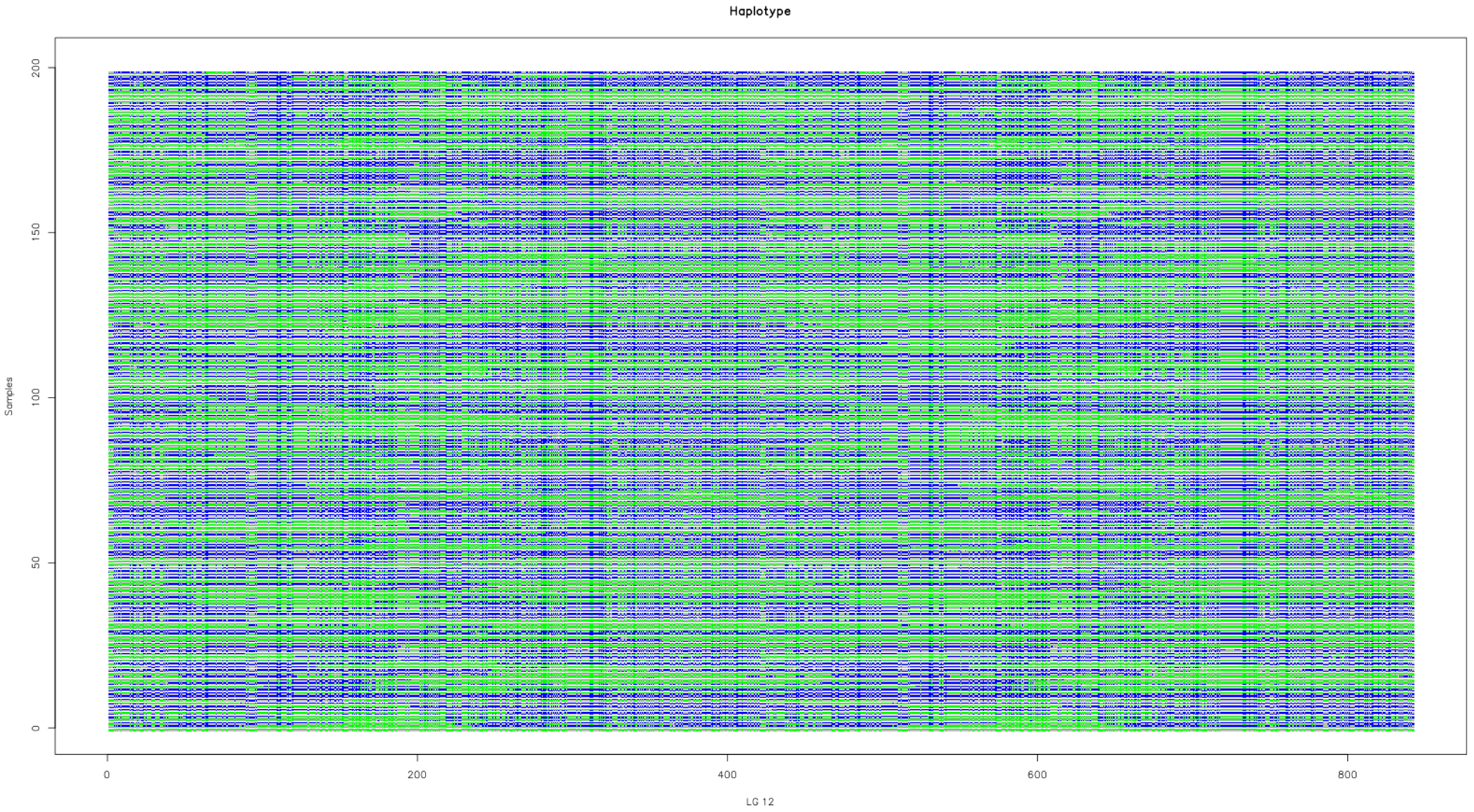
Haplotype
Samples
0
50
100
150
200
0
200
400
600
800
LG 12

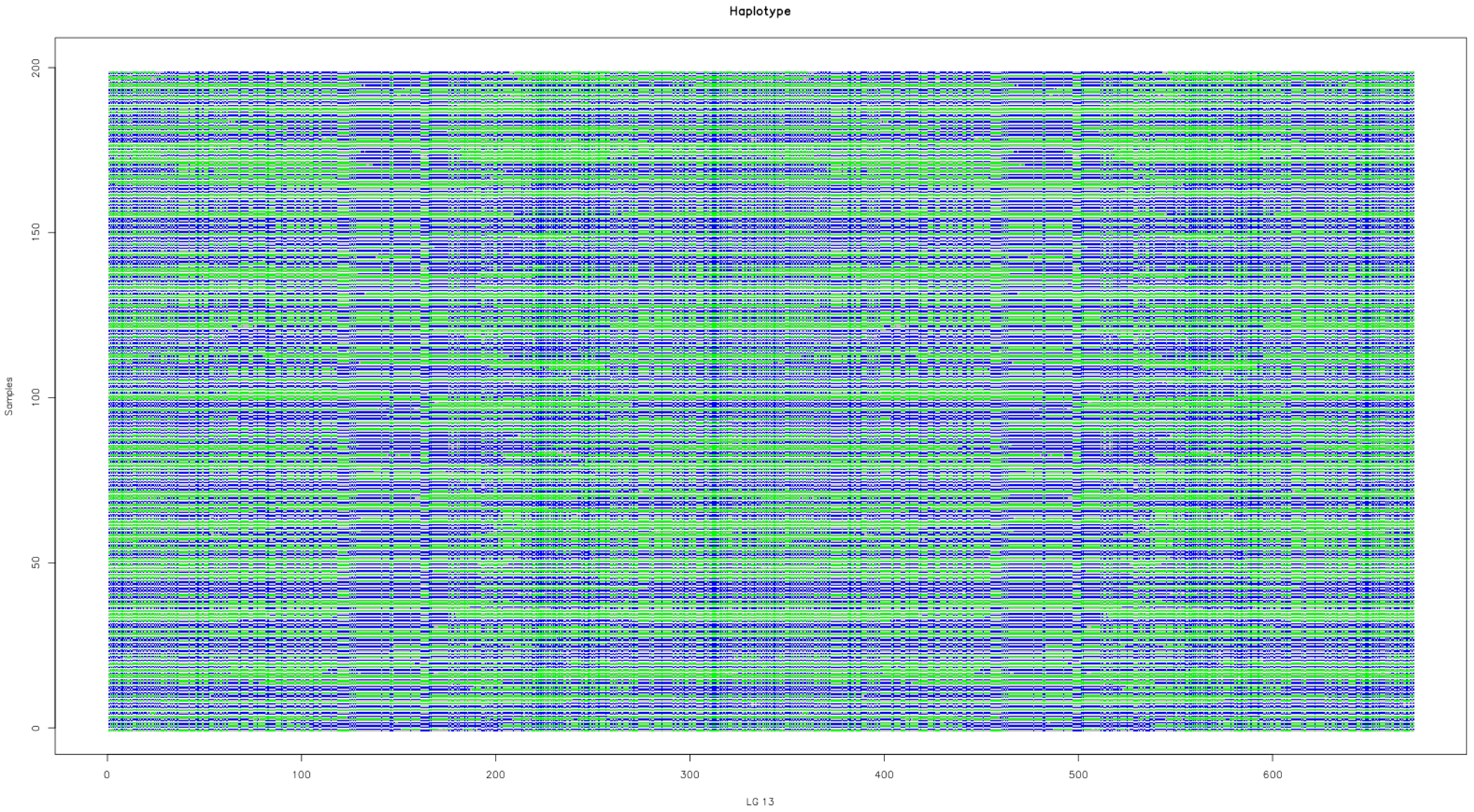
Haplotype
Samples
LG 13
0
50
100
150
200
0
100
200
300
400
500
600

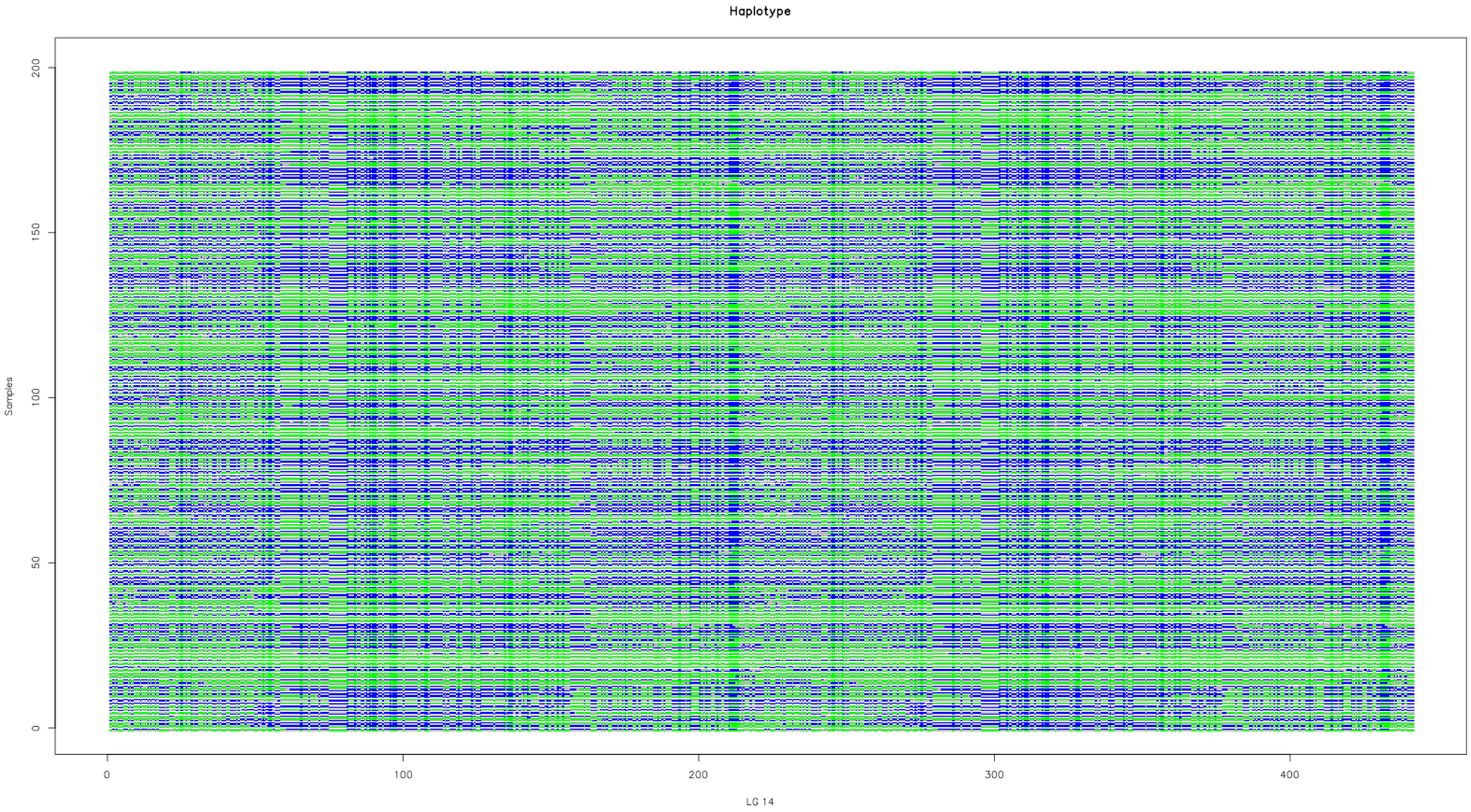

Haplotype
Samples
LG 14
0
50
100
150
200
0
100
200
300
400

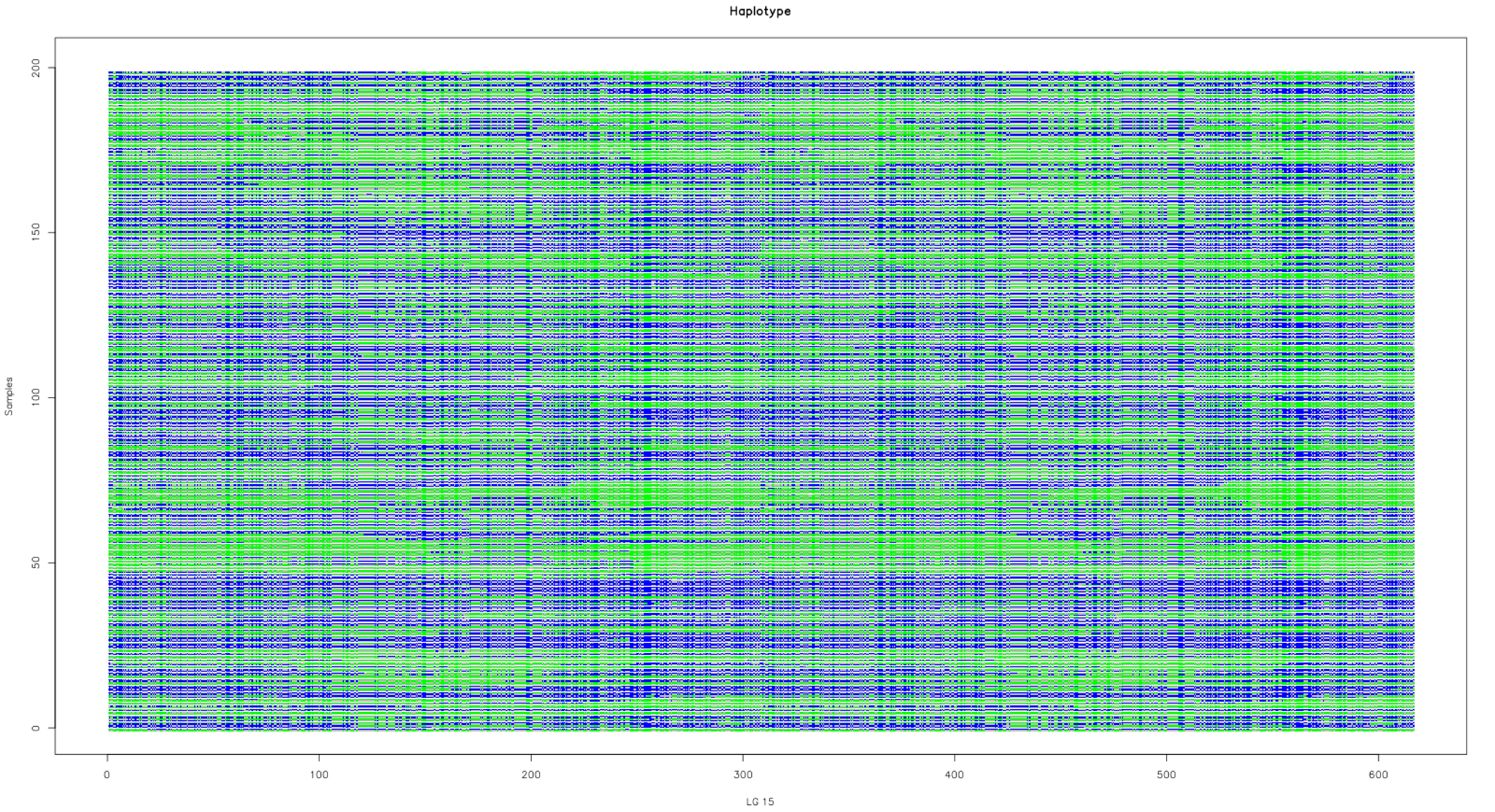
Haplotype
Samples
LG 15
0
50
100
150
200
0
100
200
300
400
500
600

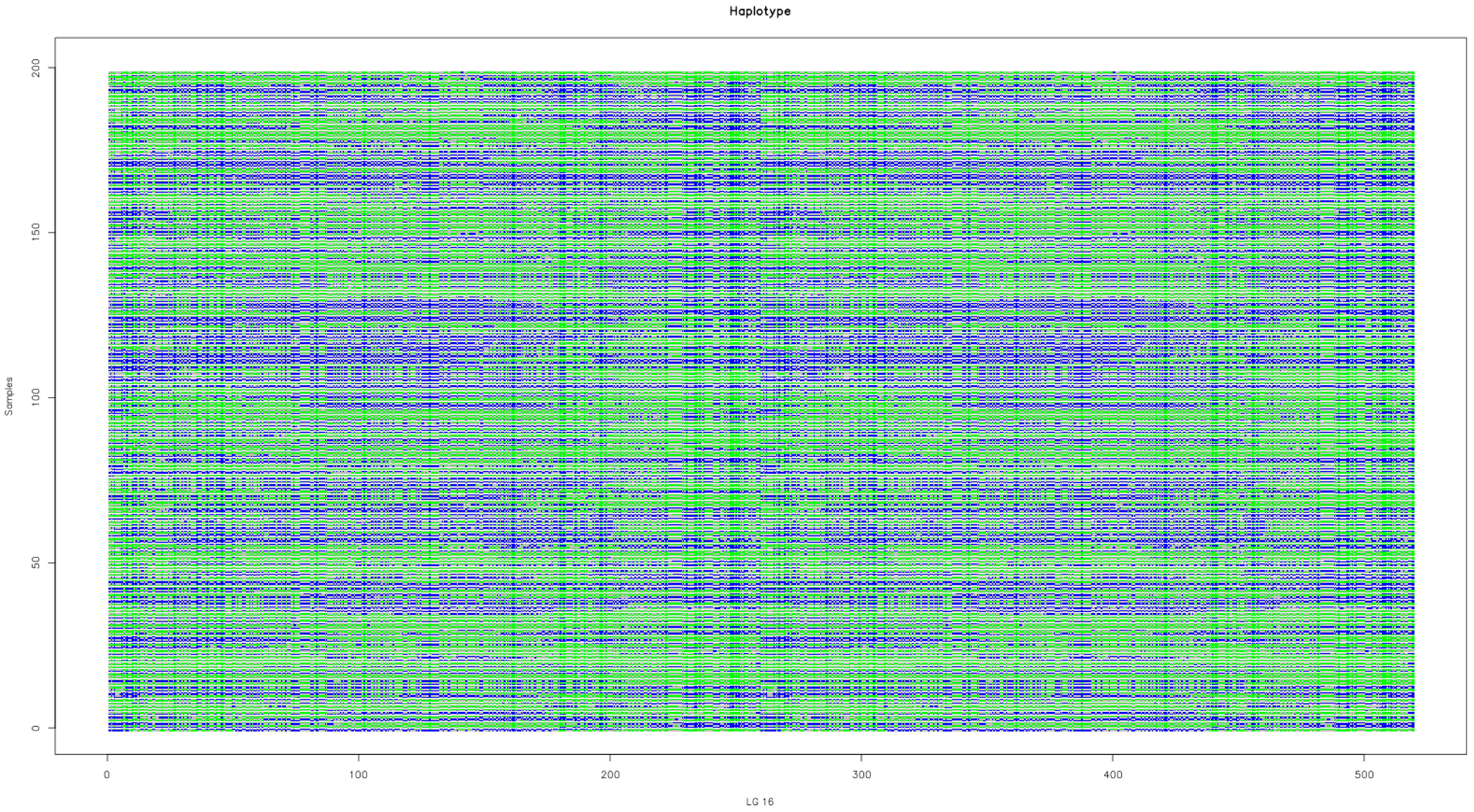
Haplotype
Samples
0
50
100
150
200
LG 16
0
100
200
300
400
500

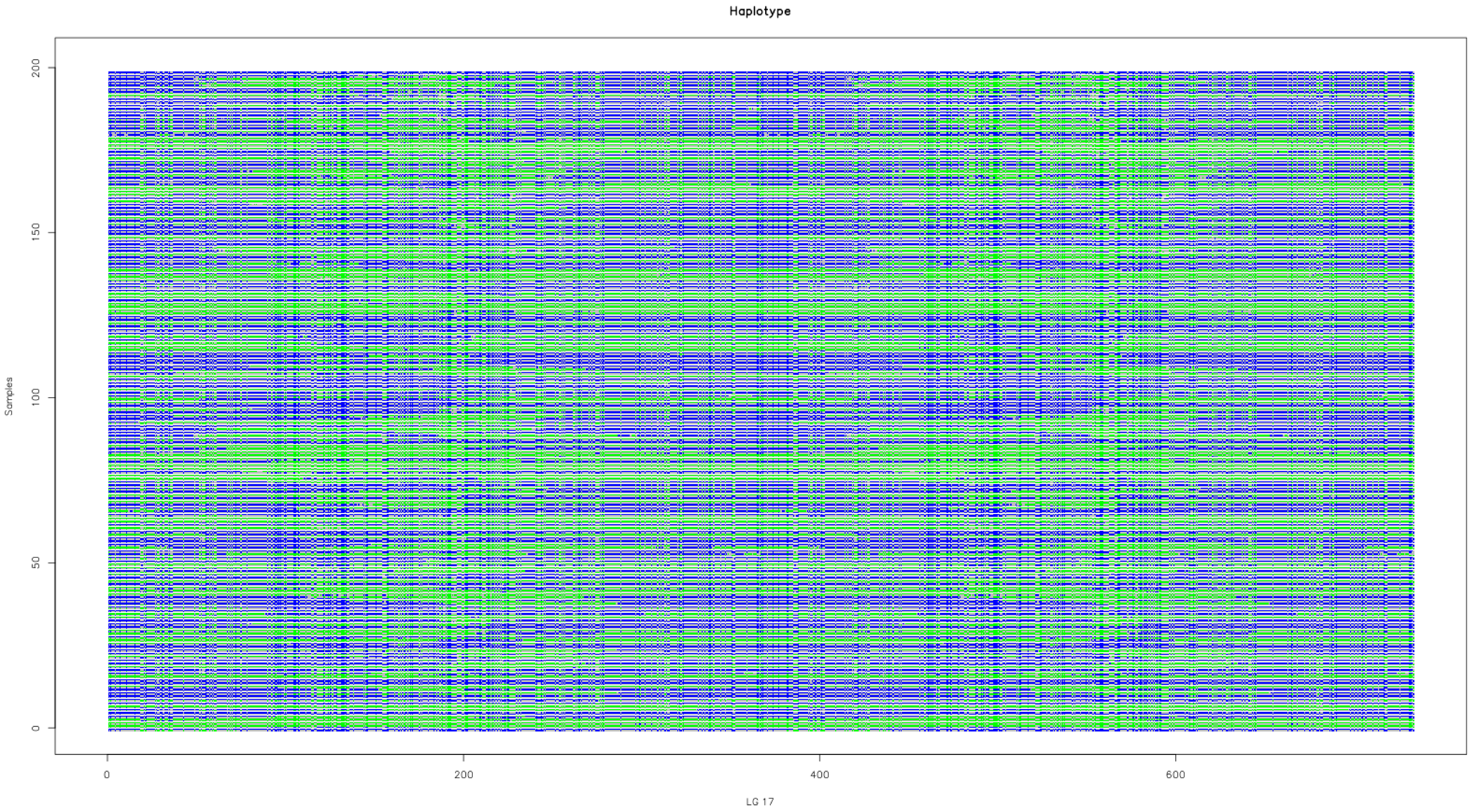
Haplotype
Samples
0
50
100
150
200
0
200
400
600
LG 17

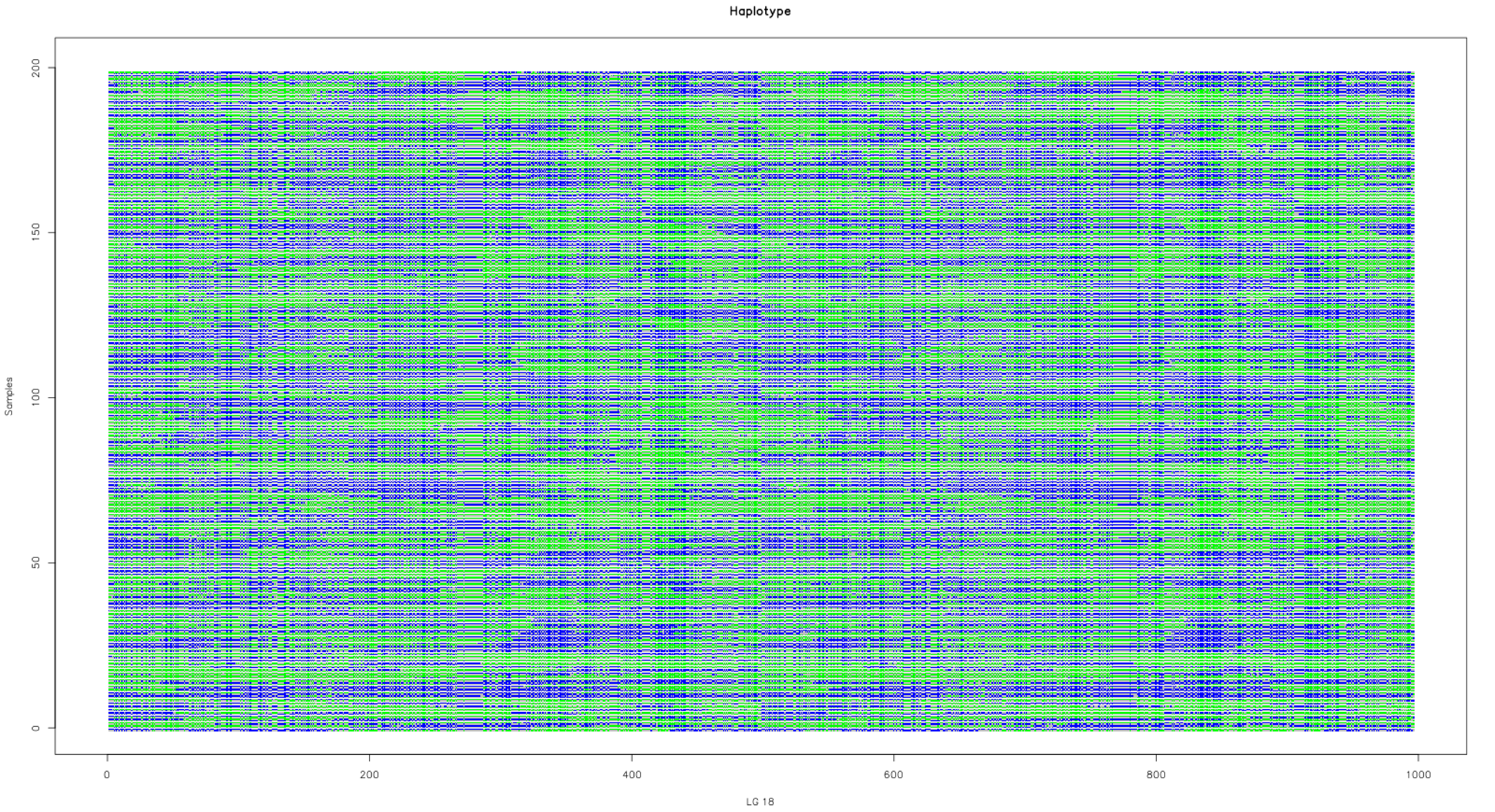

Haplotype
Samples
0
50
100
150
200
0
200
400
600
800
1000
LG 18

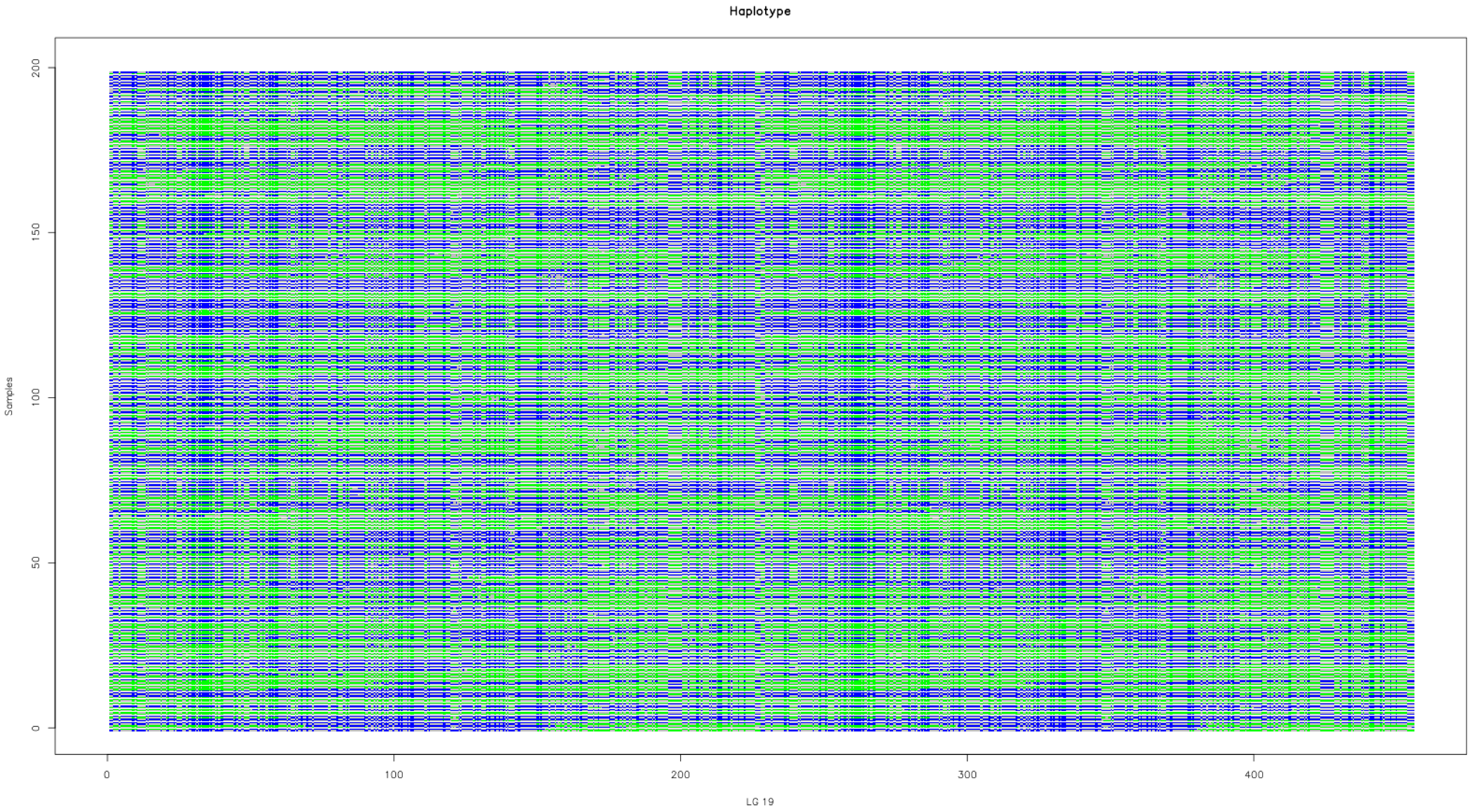

Haplotype
Samples
LG 19
0
50
100
150
200
0
100
200
300
400

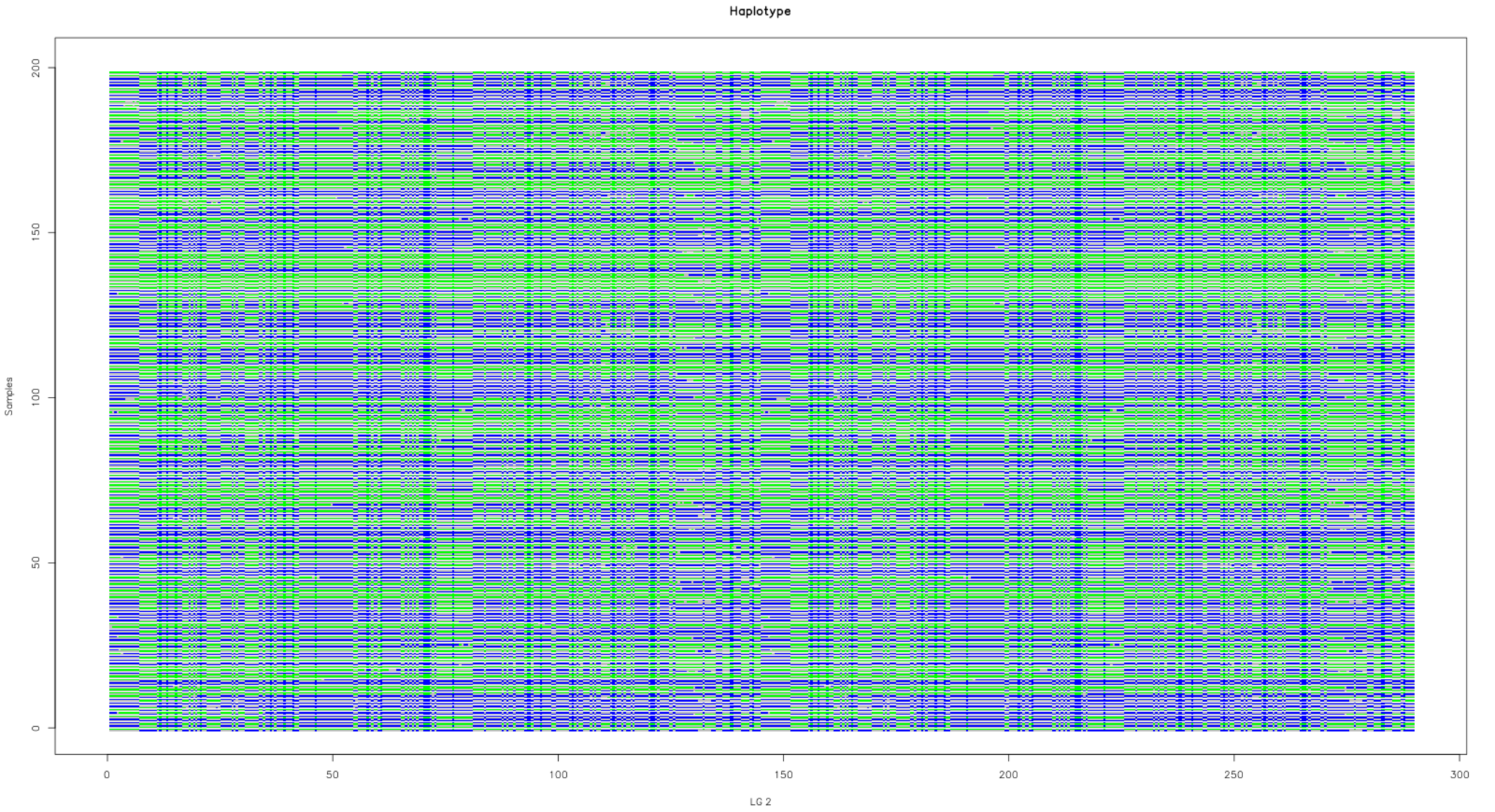
Haplotype
Samples
LG 2
0
50
100
150
200
250
300

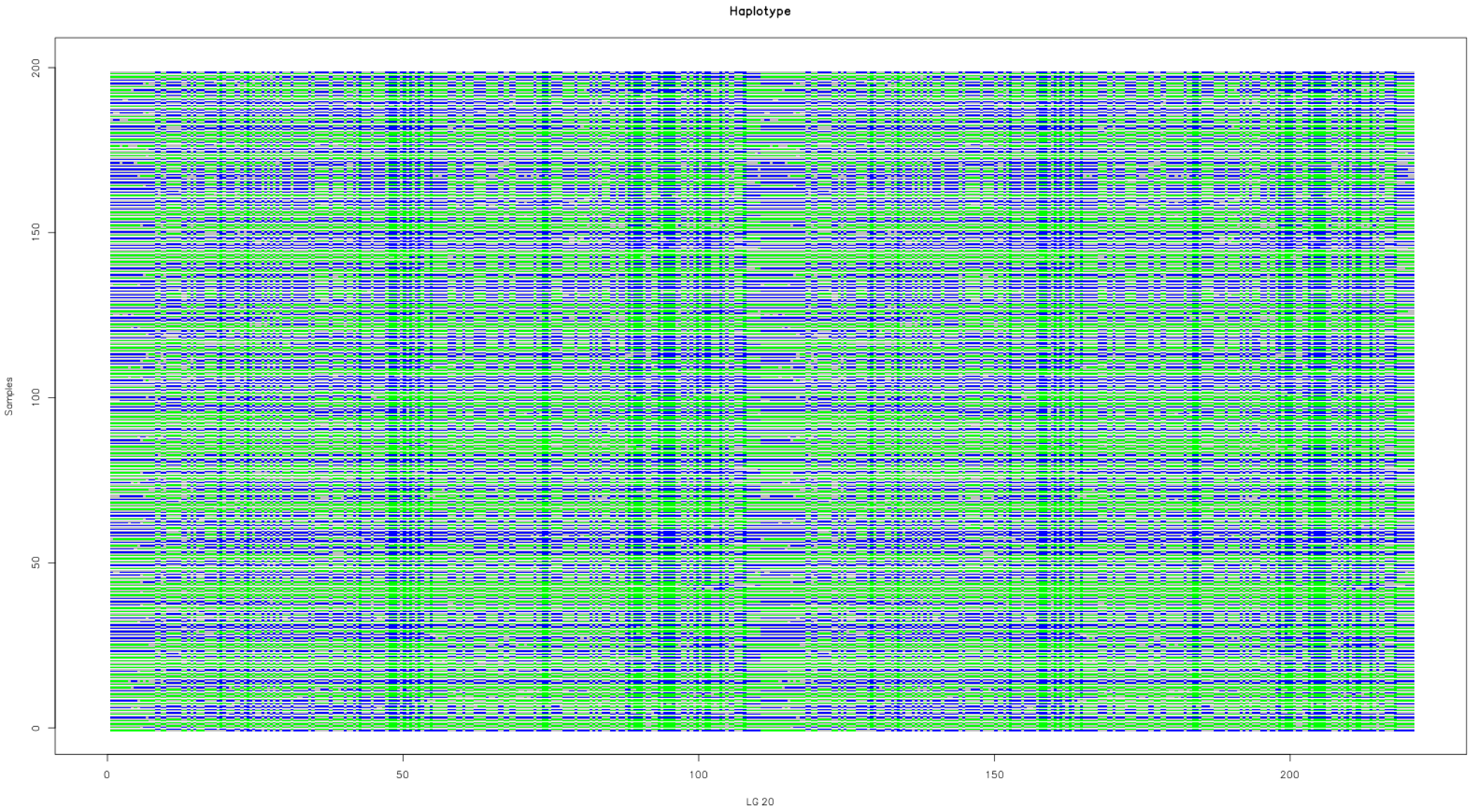
Haplotype
Samples
LG 20
0
50
100
150
200
0
50
100
150
200

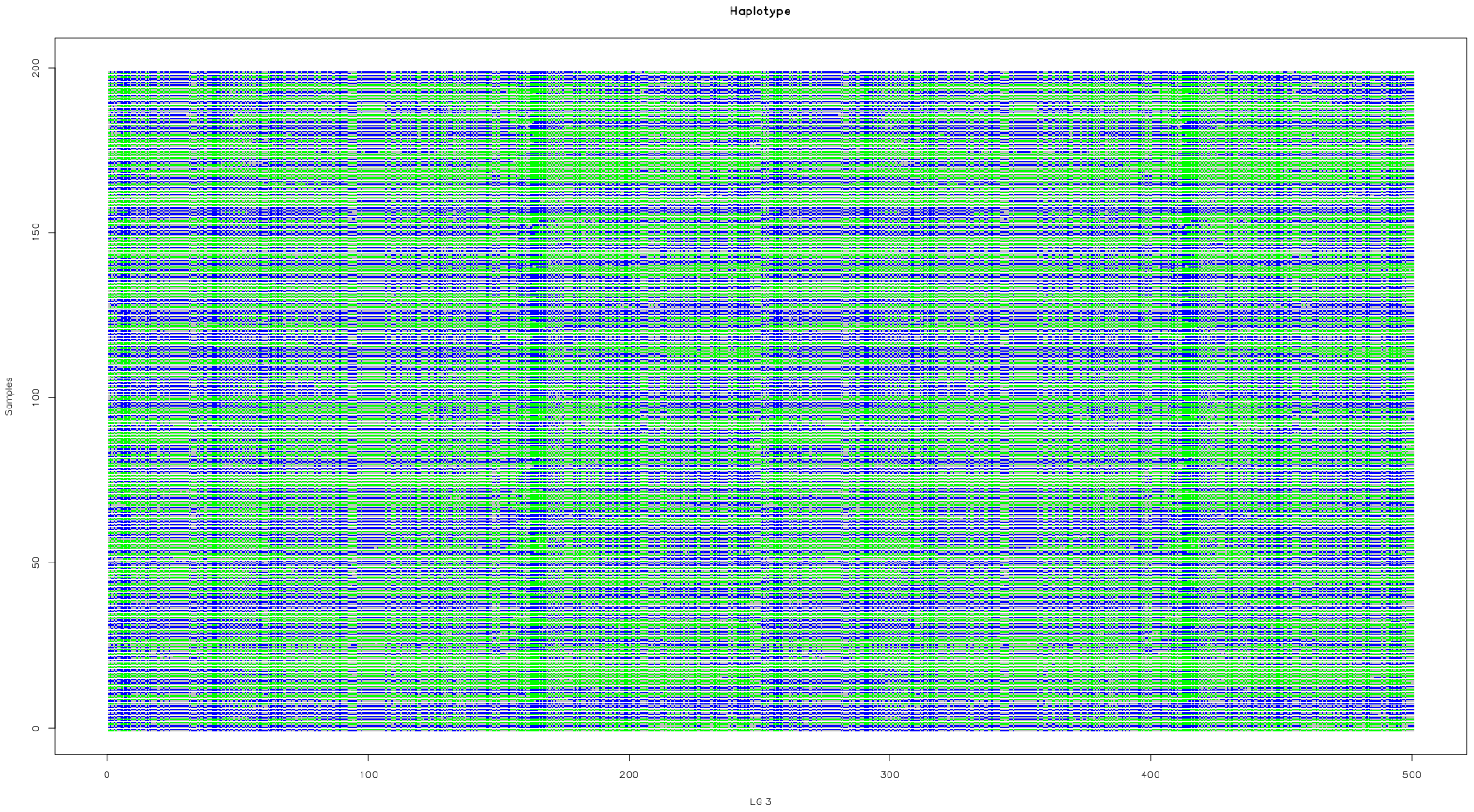
Haplotype
Samples
200
150
100
50
0
0
100
200
300
400
500
LG 3

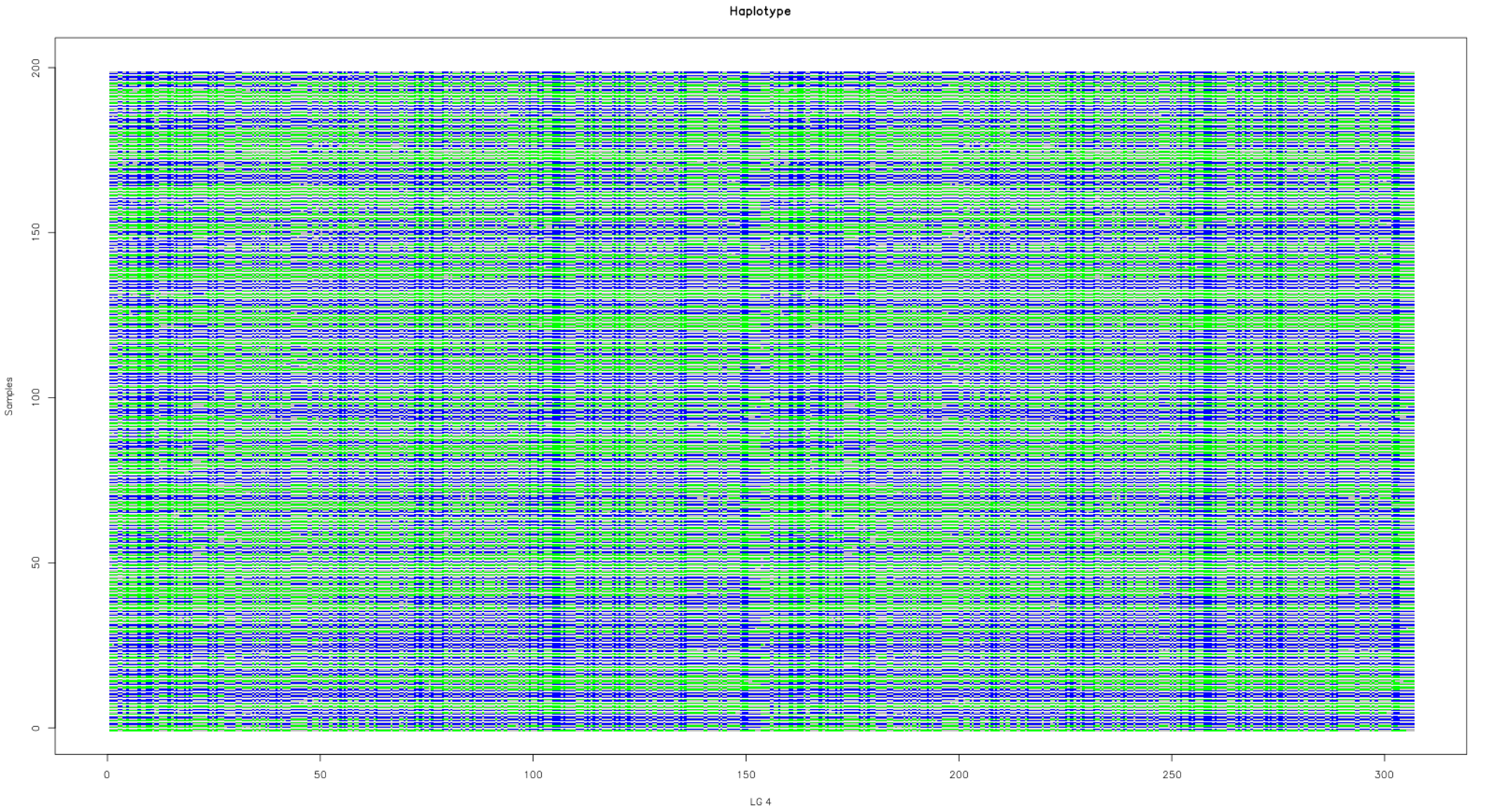
Haplotype
Samples
LG 4
0
50
100
150
200
0
50
100
150
200
250
300

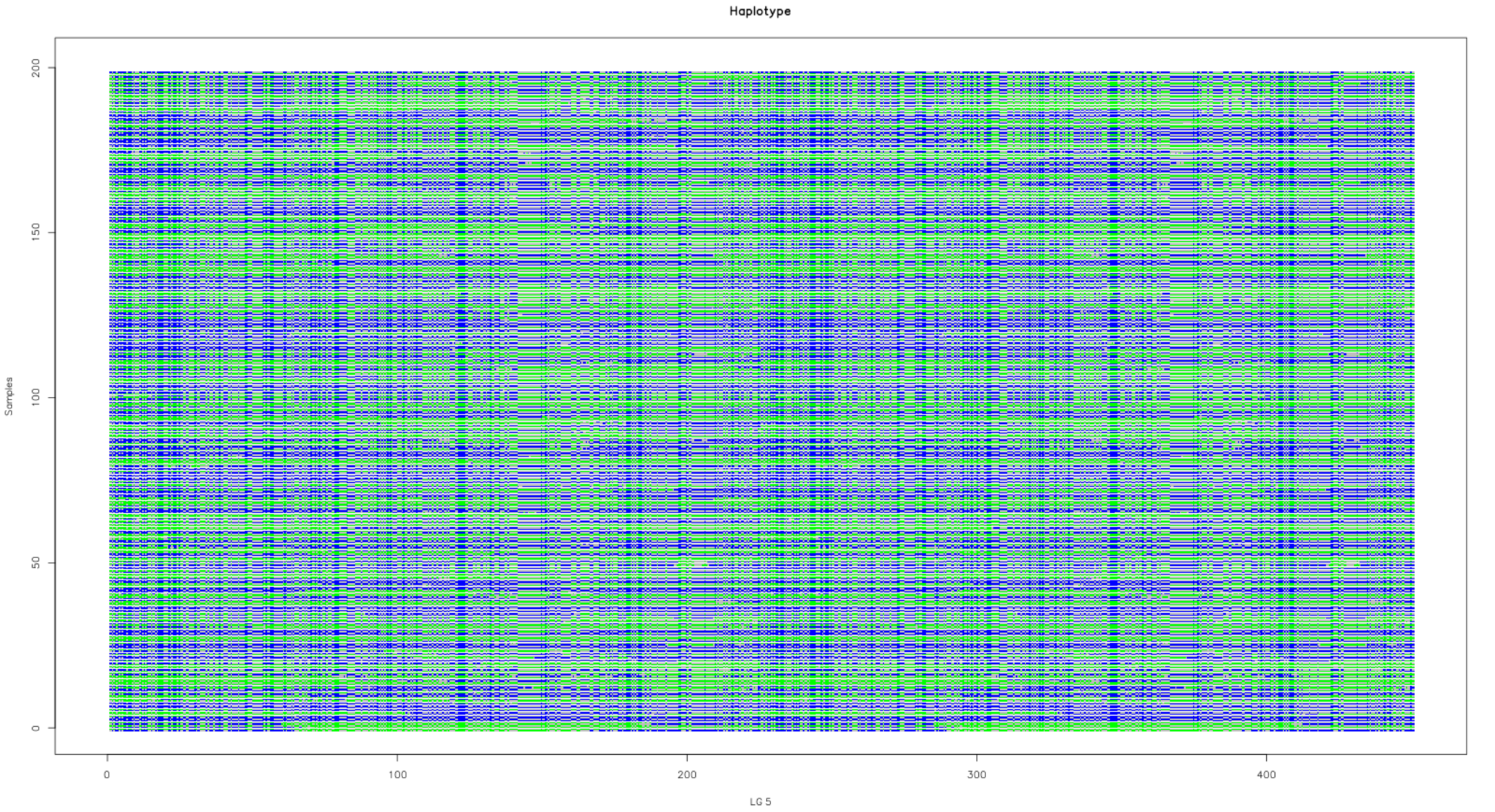
Haplotype
Samples
LG 5
0
50
100
150
200
0
100
200
300
400

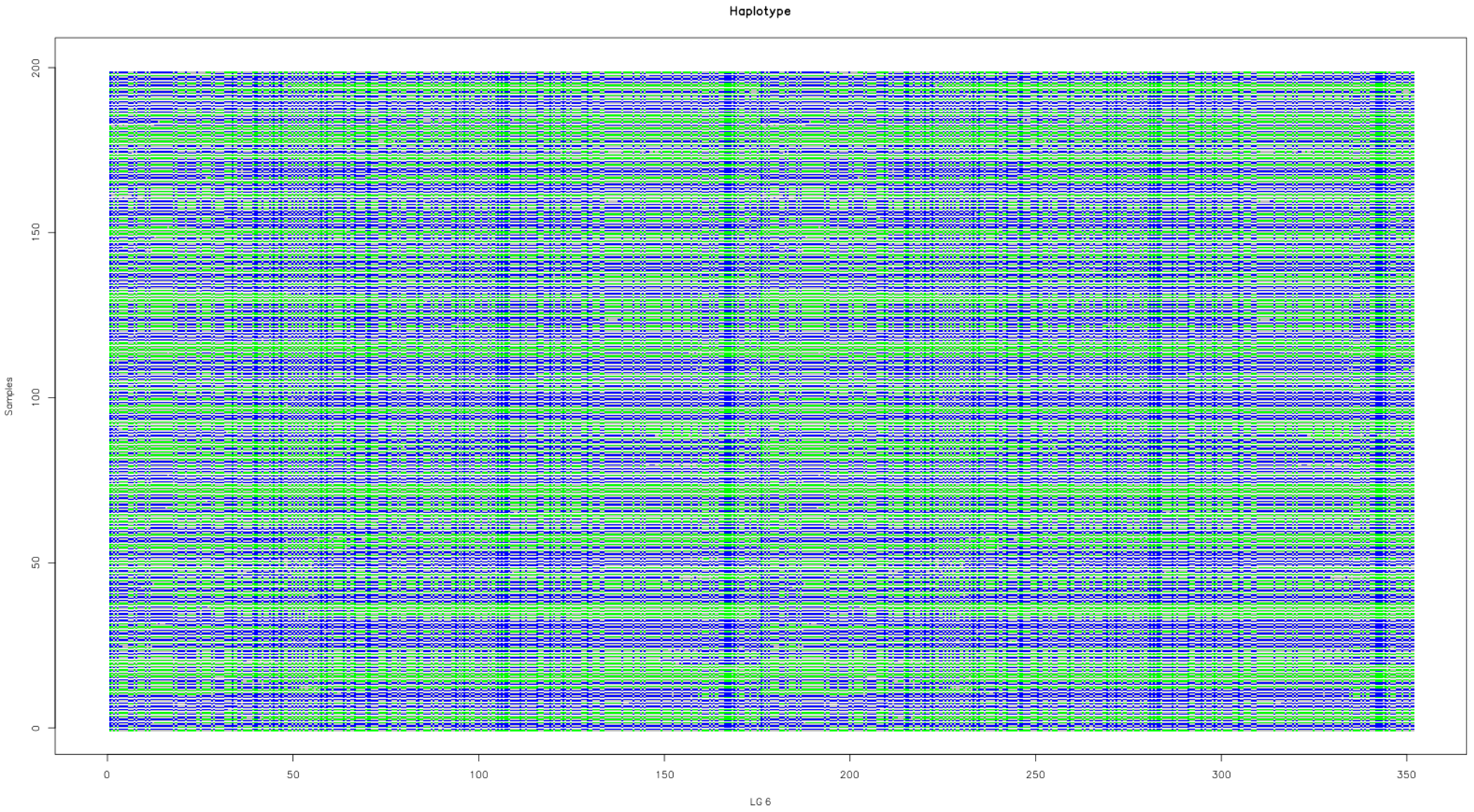

Haplotype
Samples
LG 6
0
50
100
150
200
250
300
350

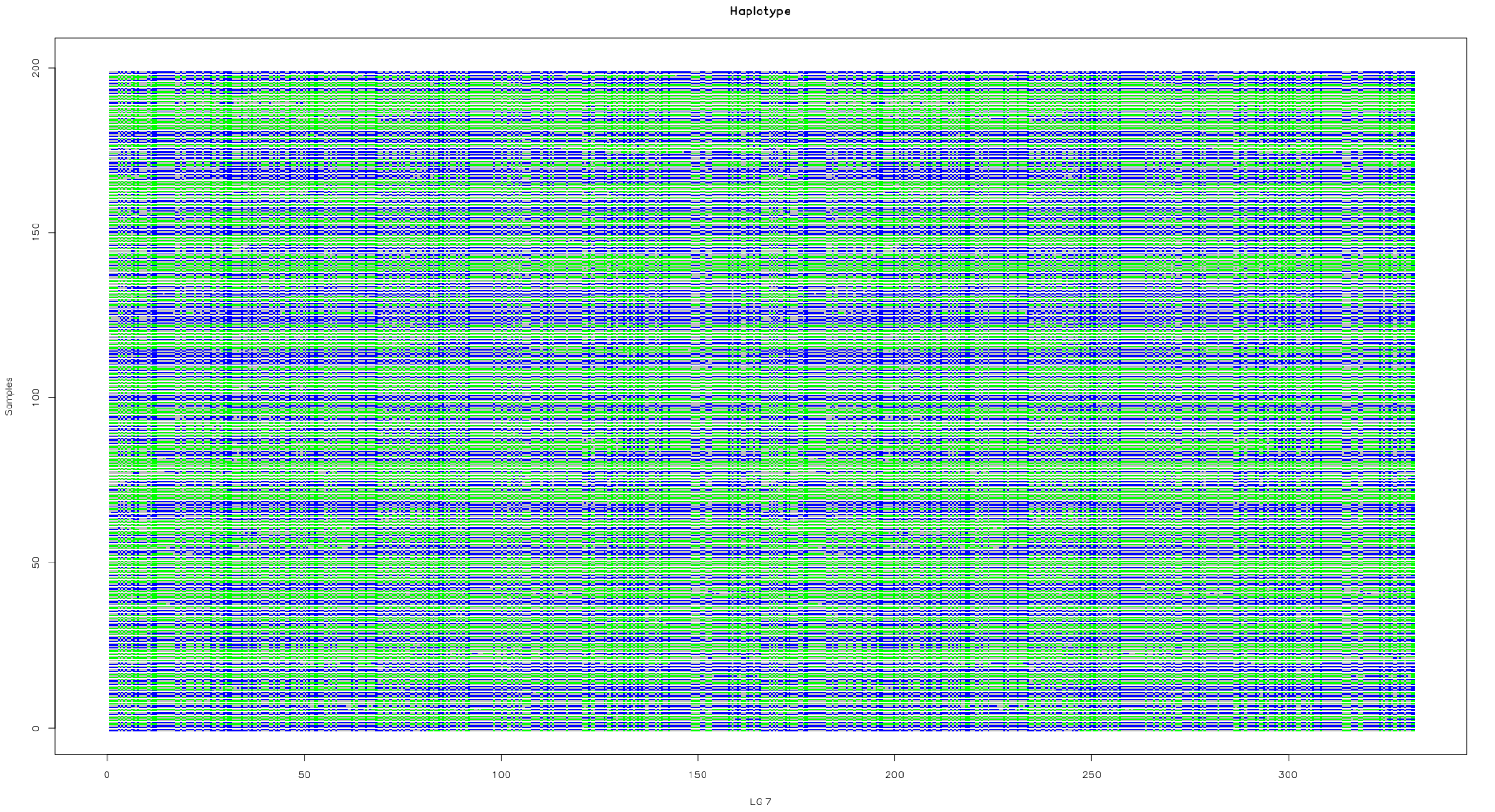
Haplotype
Samples
LG 7
0
50
100
150
200
250
300

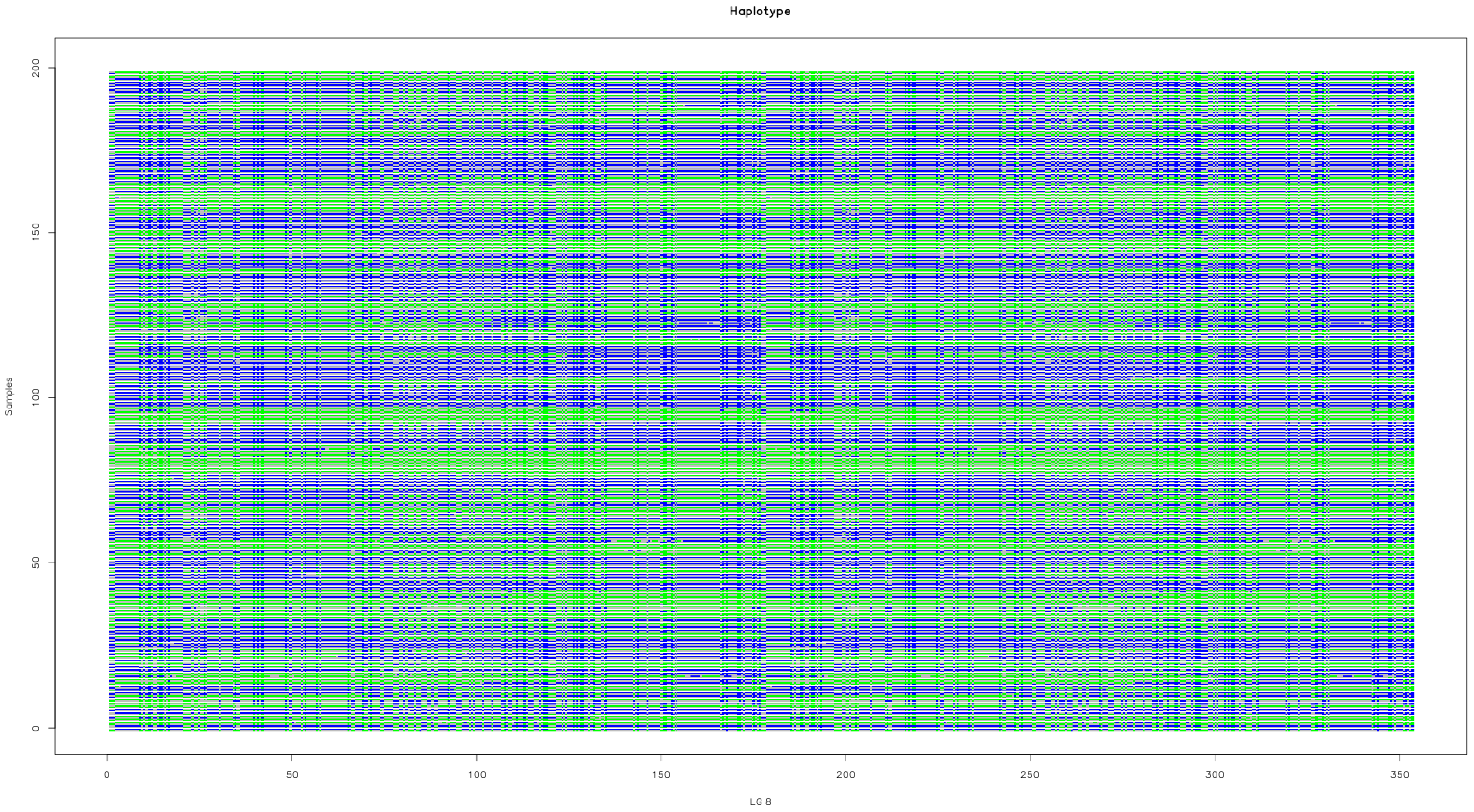
Haplotype
Samples
0
50
100
150
200
0
50
100
150
200
250
300
350
LG 8

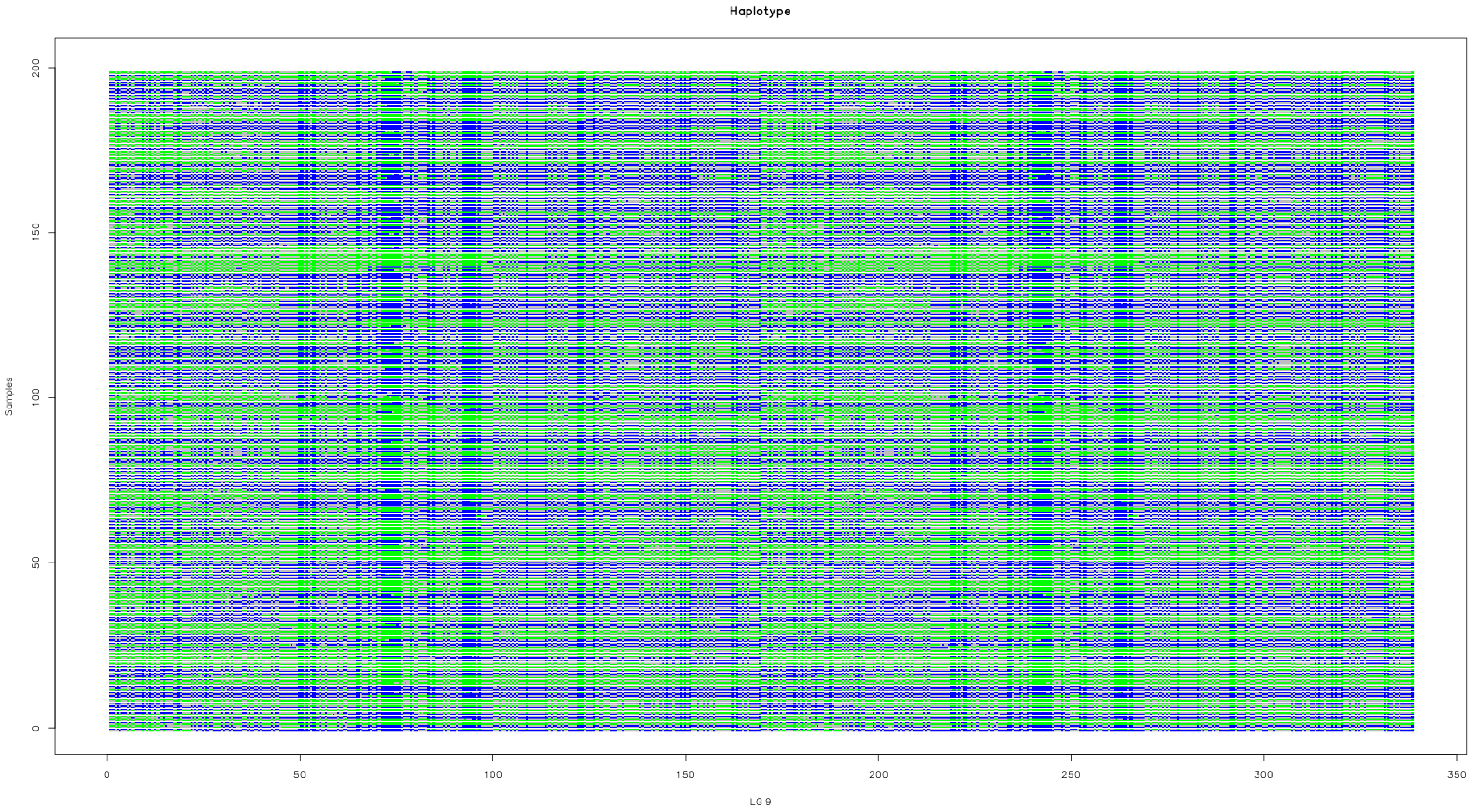
Haplotype
Samples
LG 9
0
50
100
150
200
250
300
350
