# Supplementary material for: Construction of a high-density genetic map and QTL mapping of leaf traits and plant growth in an interspecific F1 population of Catalpa bungei × Catalpa duclouxii Dode
Source: BMC Plant Biol. 2019 Dec 30;19:596. doi: 10.1186/s12870-019-2207-y (PMC6937828; doi:10.1186/s12870-019-2207-y)
Supplement: Supplementary file 6 — Additional file 6: Figure S2. Heat maps of the integrated genetic map. Each cell represents the pairwise LOD scores of two markers. [file 12870_2019_2207_MOESM6_ESM.pdf]

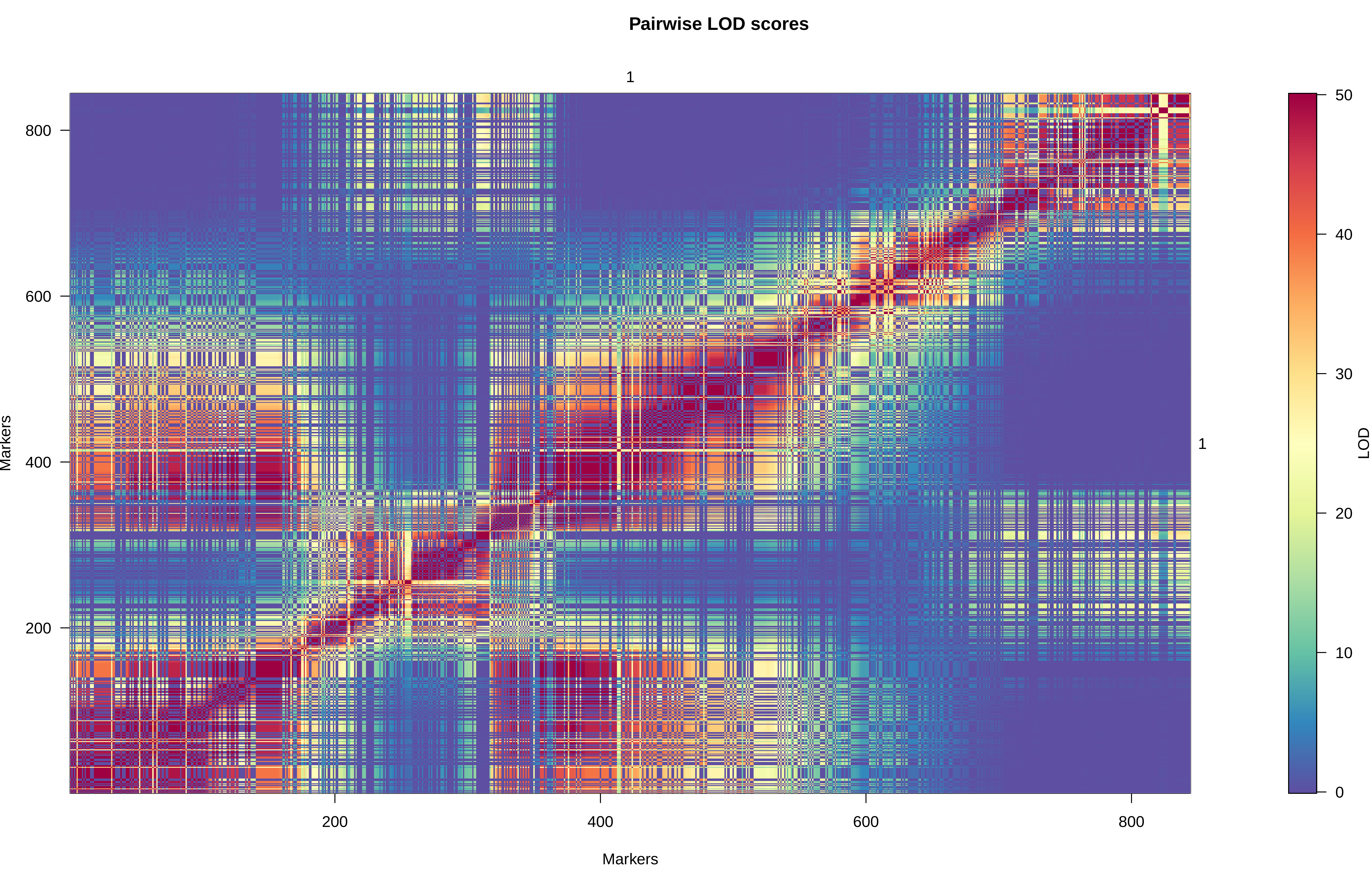
Pairwise LOD scores
1
1
Markers
Markers
200
400
600
800
200
400
600
800
LOD
0
10
20
30
40
50

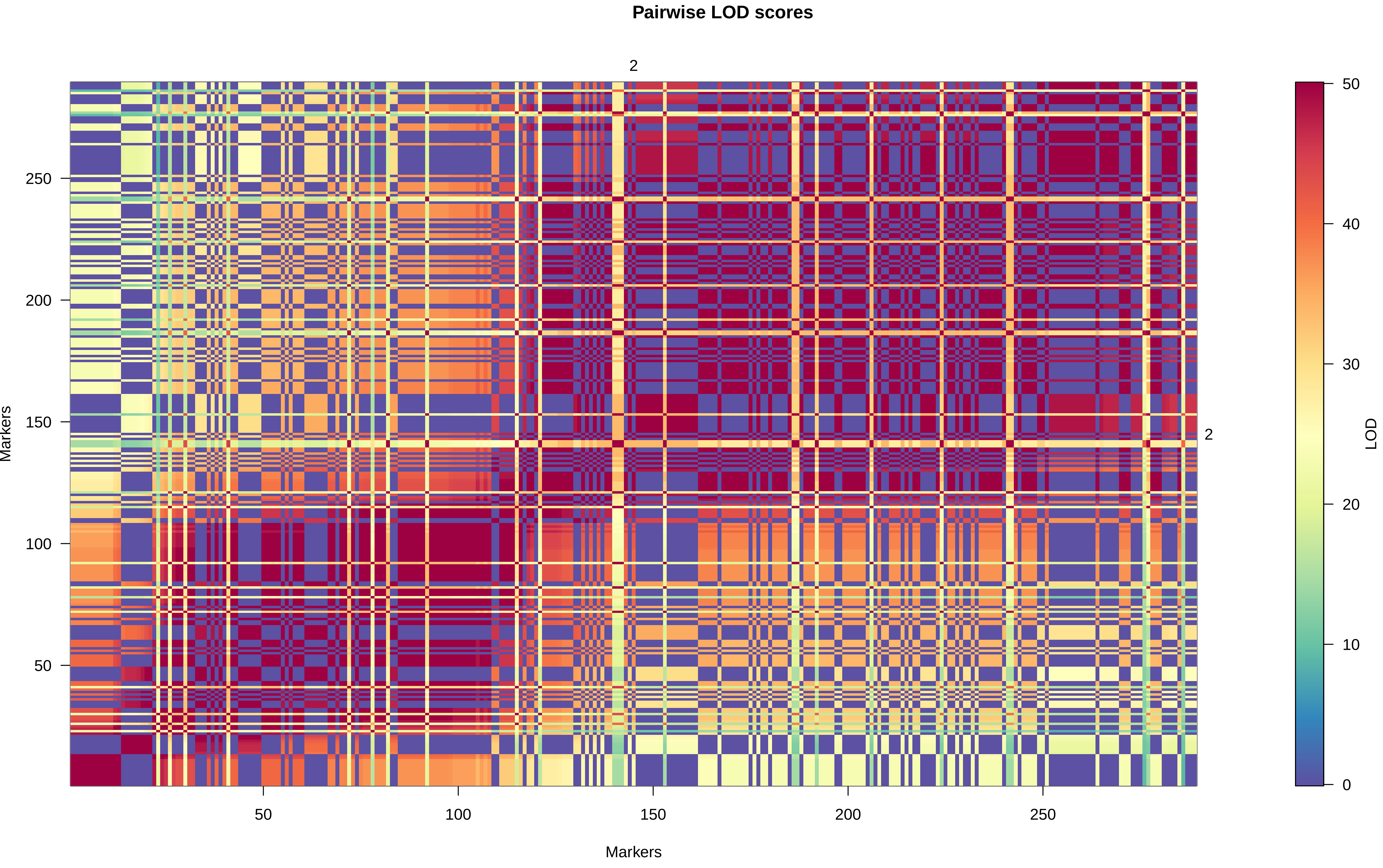
Pairwise LOD scores
2
2
250
200
150
100
50
Markers
50
100
150
200
250
Markers
50
40
30
20
10
0
LOD

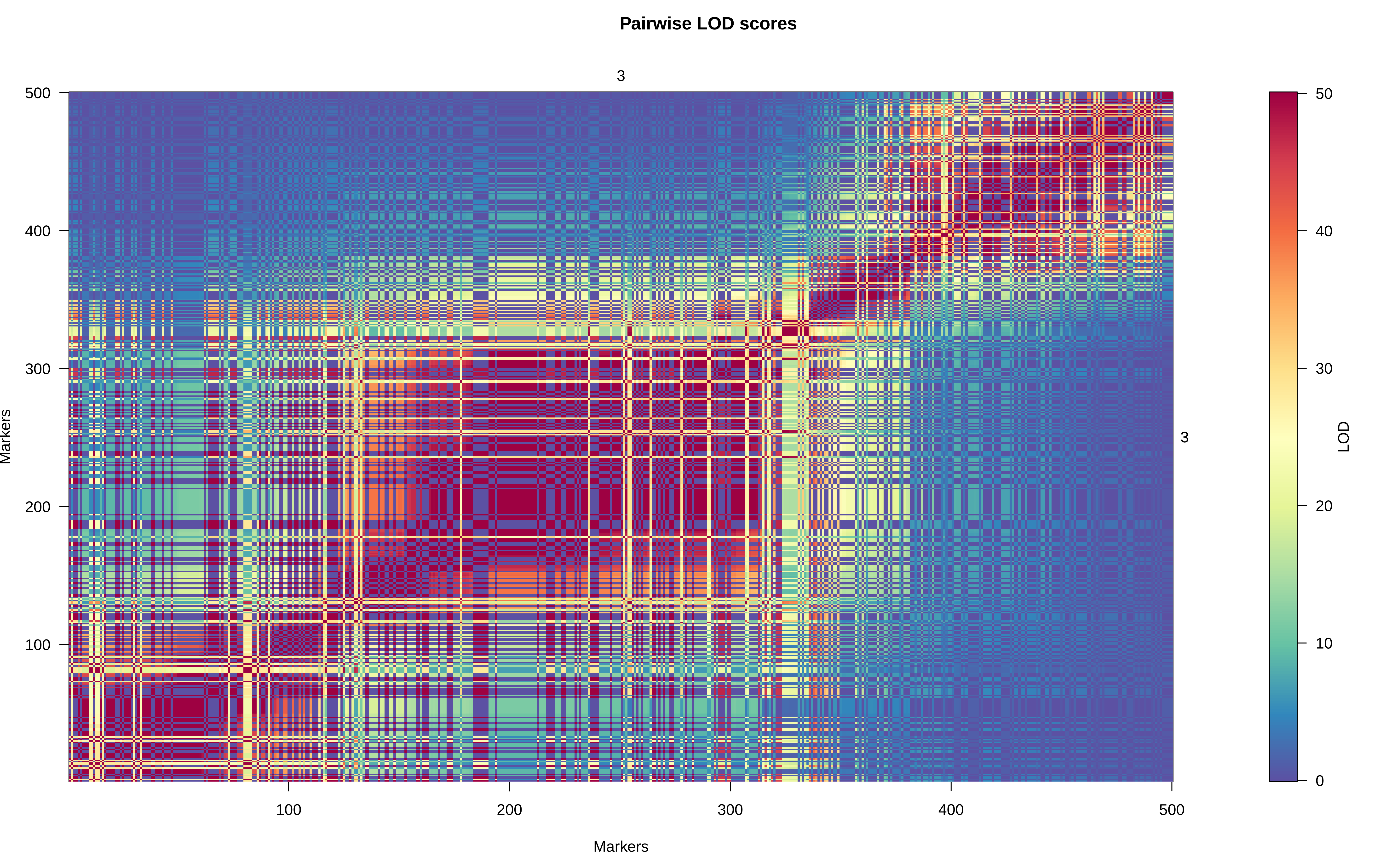
Pairwise LOD scores
3
3
500
400
300
200
100
100
200
300
400
500
Markers
Markers
50
40
30
20
10
0
LOD

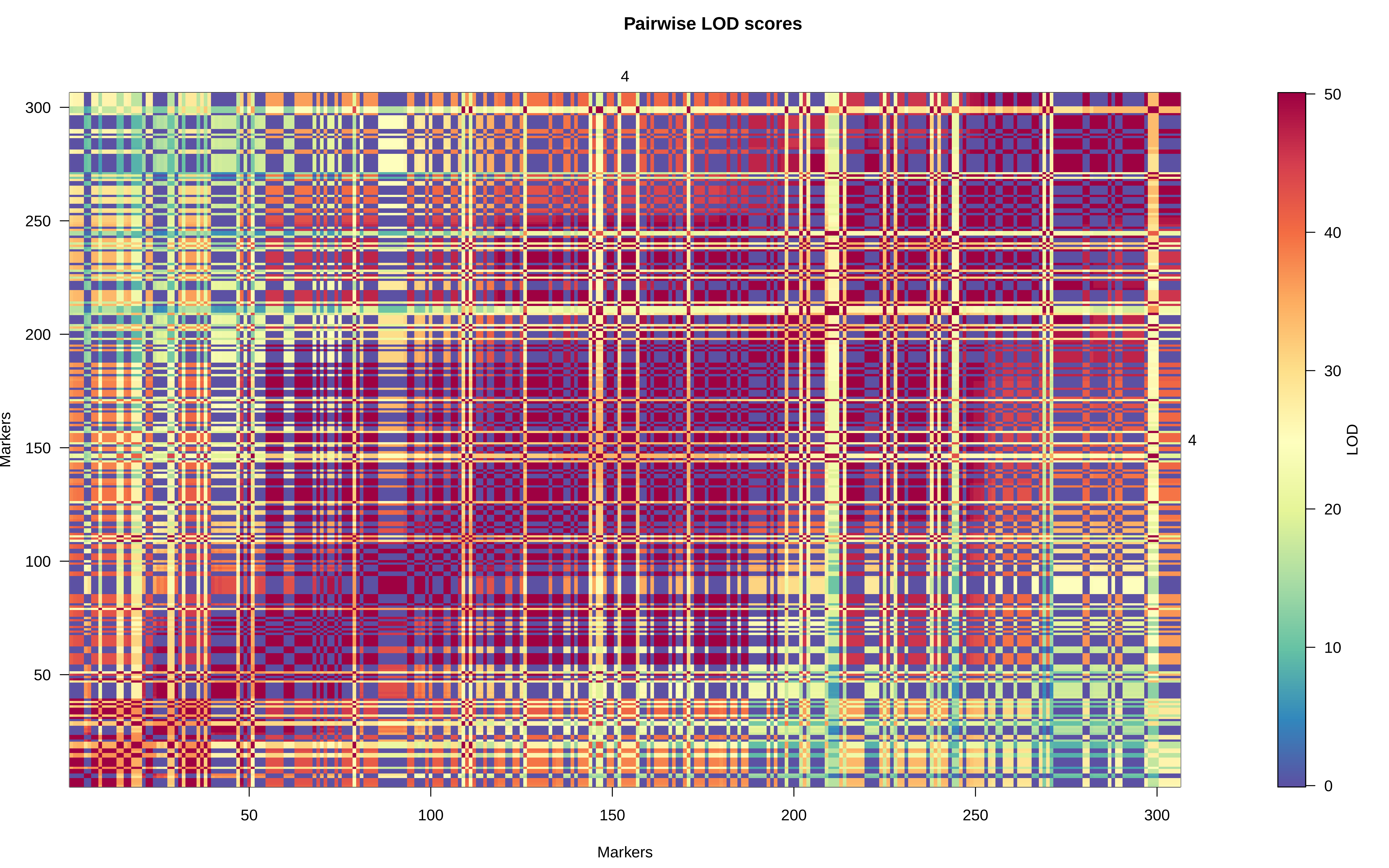

Pairwise LOD scores
4
4
Markers
Markers
300
250
200
150
100
50
50
100
150
200
250
300
LOD
50
40
30
20
10
0

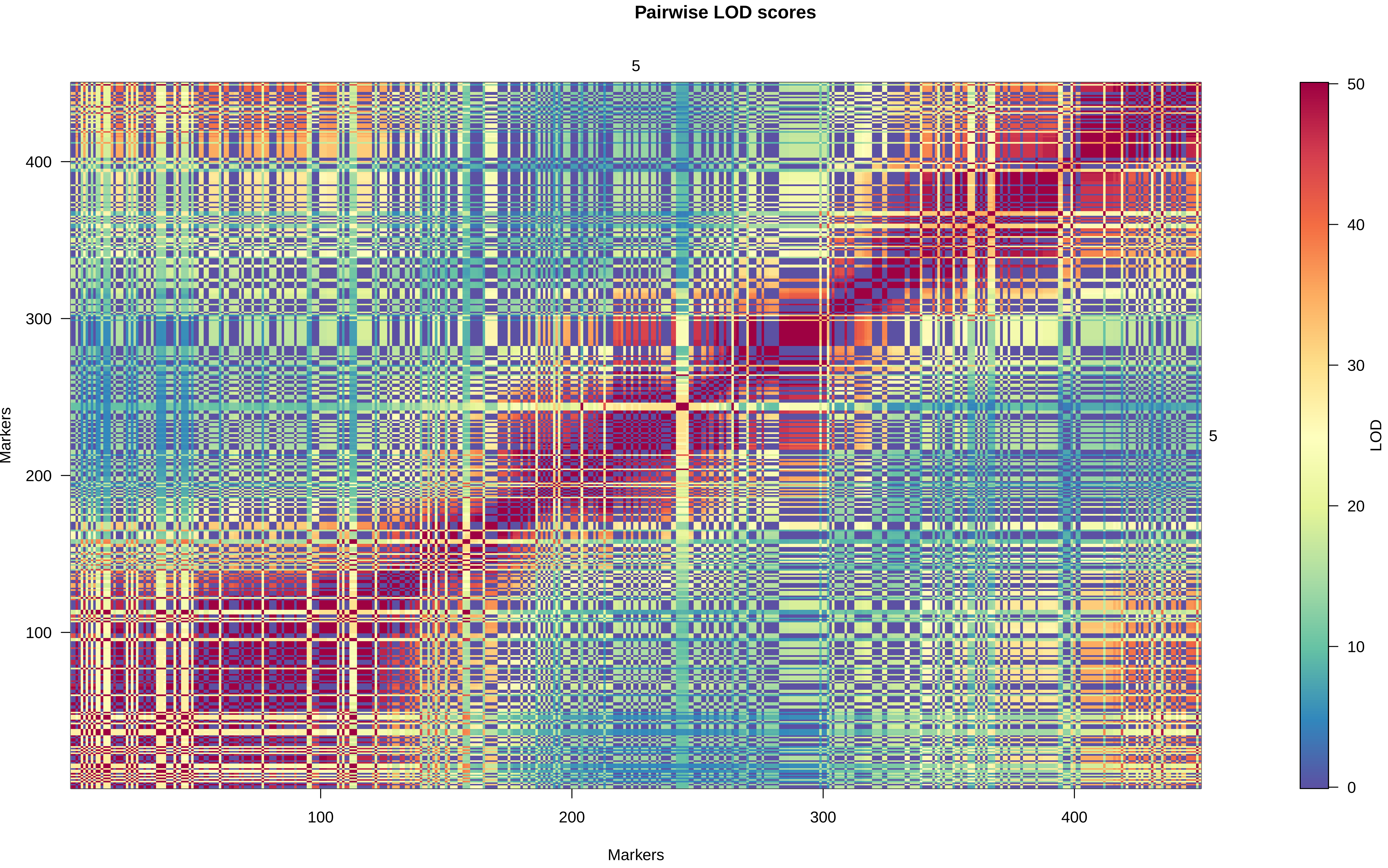

Pairwise LOD scores
5
400
300
200
100
Markers
100
200
300
400
Markers
5
50
40
30
20
10
0
LOD

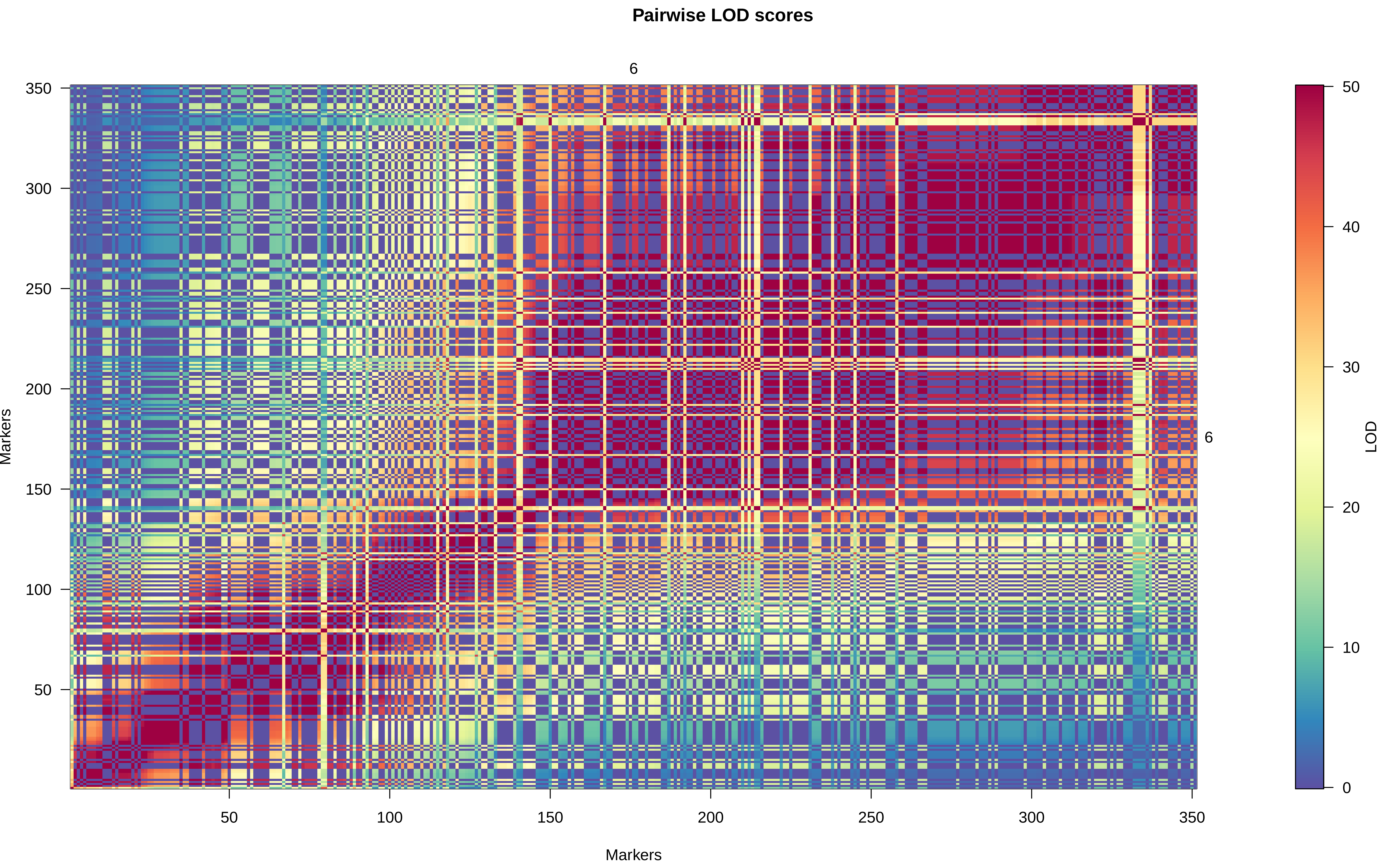
Pairwise LOD scores
6
6
Markers
Markers
50
100
150
200
250
300
350
50
100
150
200
250
300
350
LOD
0
10
20
30
40
50

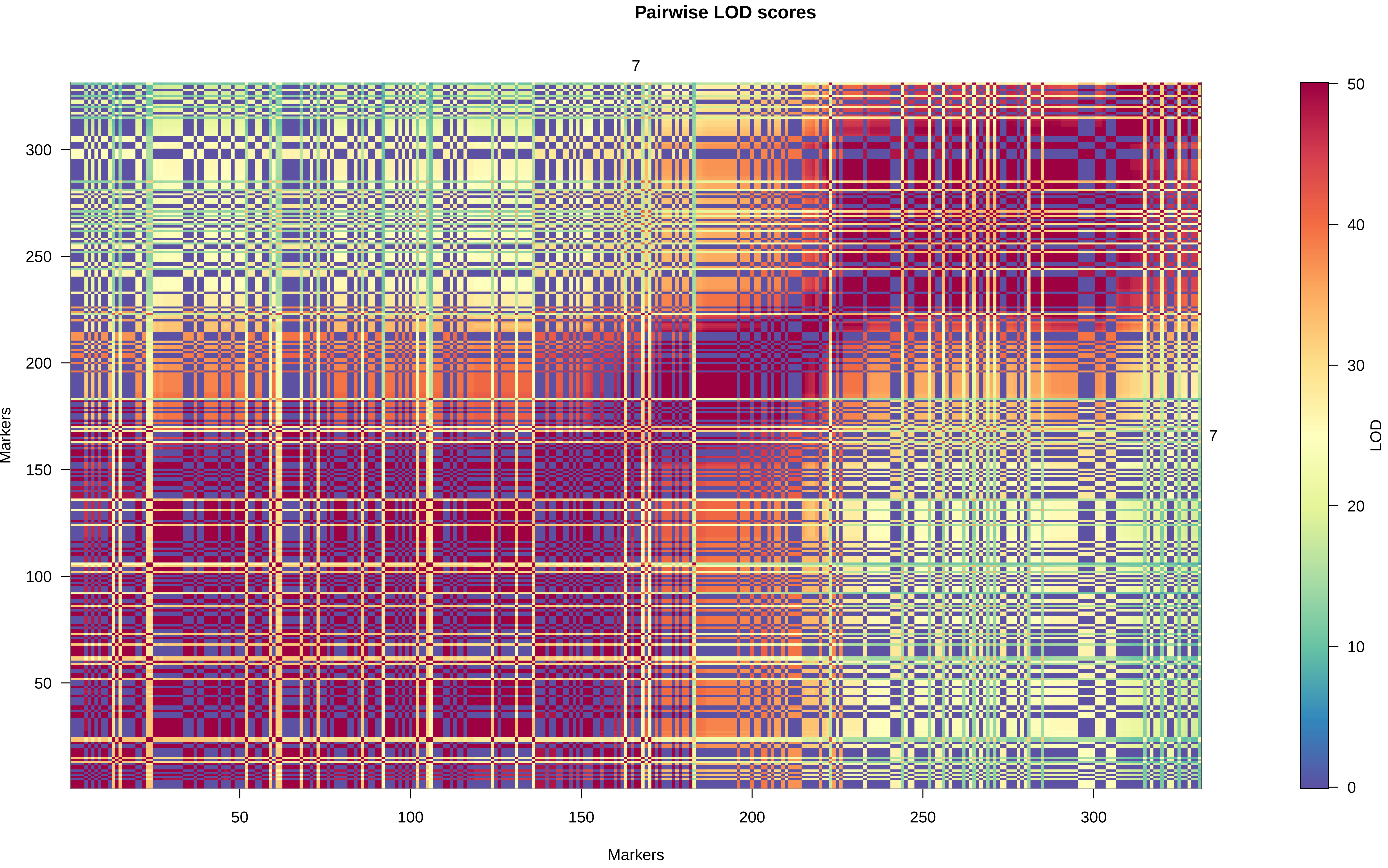

Pairwise LOD scores
7
7
Markers
Markers
50
100
150
200
250
300
50
100
150
200
250
300
LOD
0
10
20
30
40
50

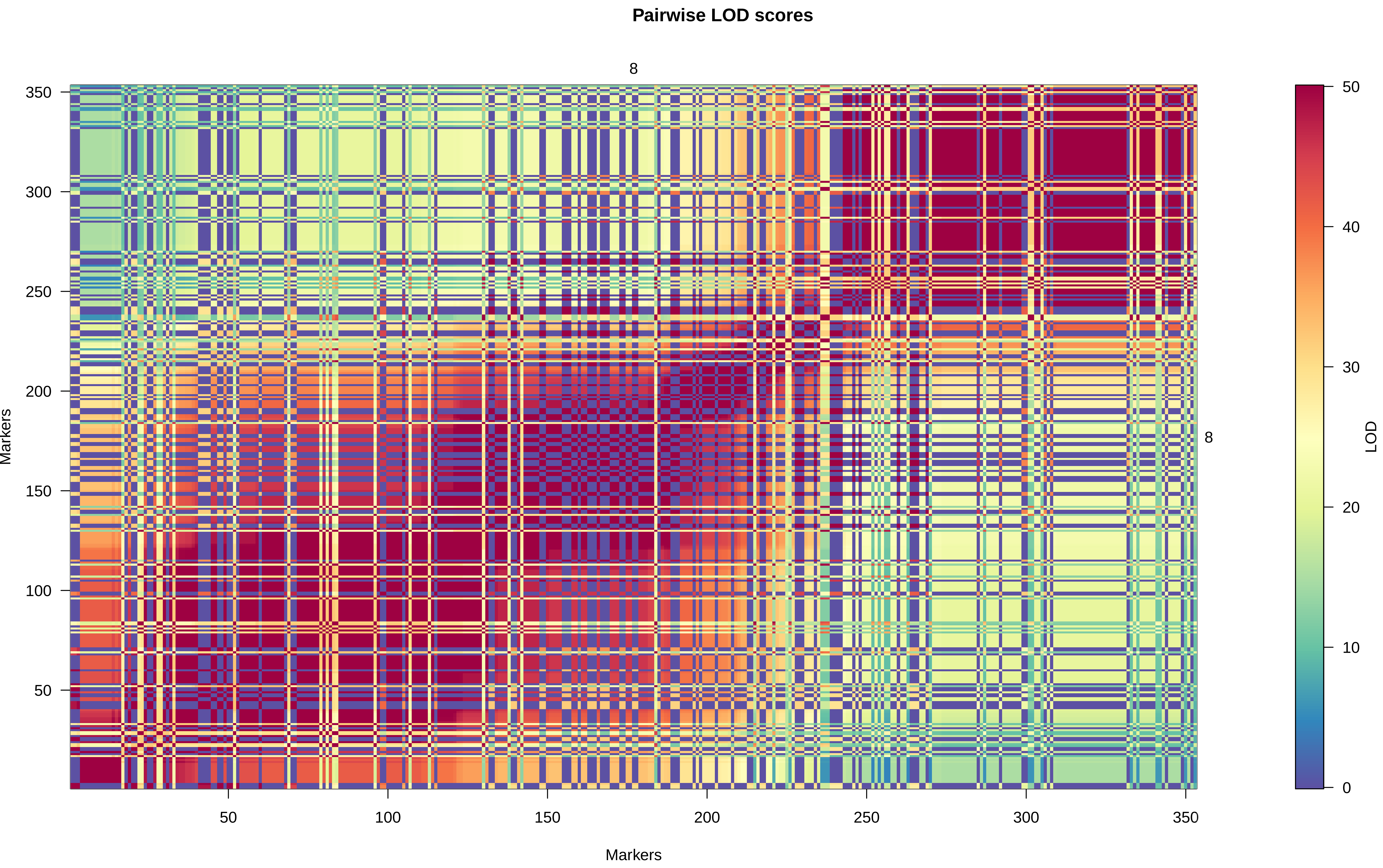
Pairwise LOD scores
8
8
Markers
Markers
350
300
250
200
150
100
50
50
100
150
200
250
300
350
LOD
50
40
30
20
10
0

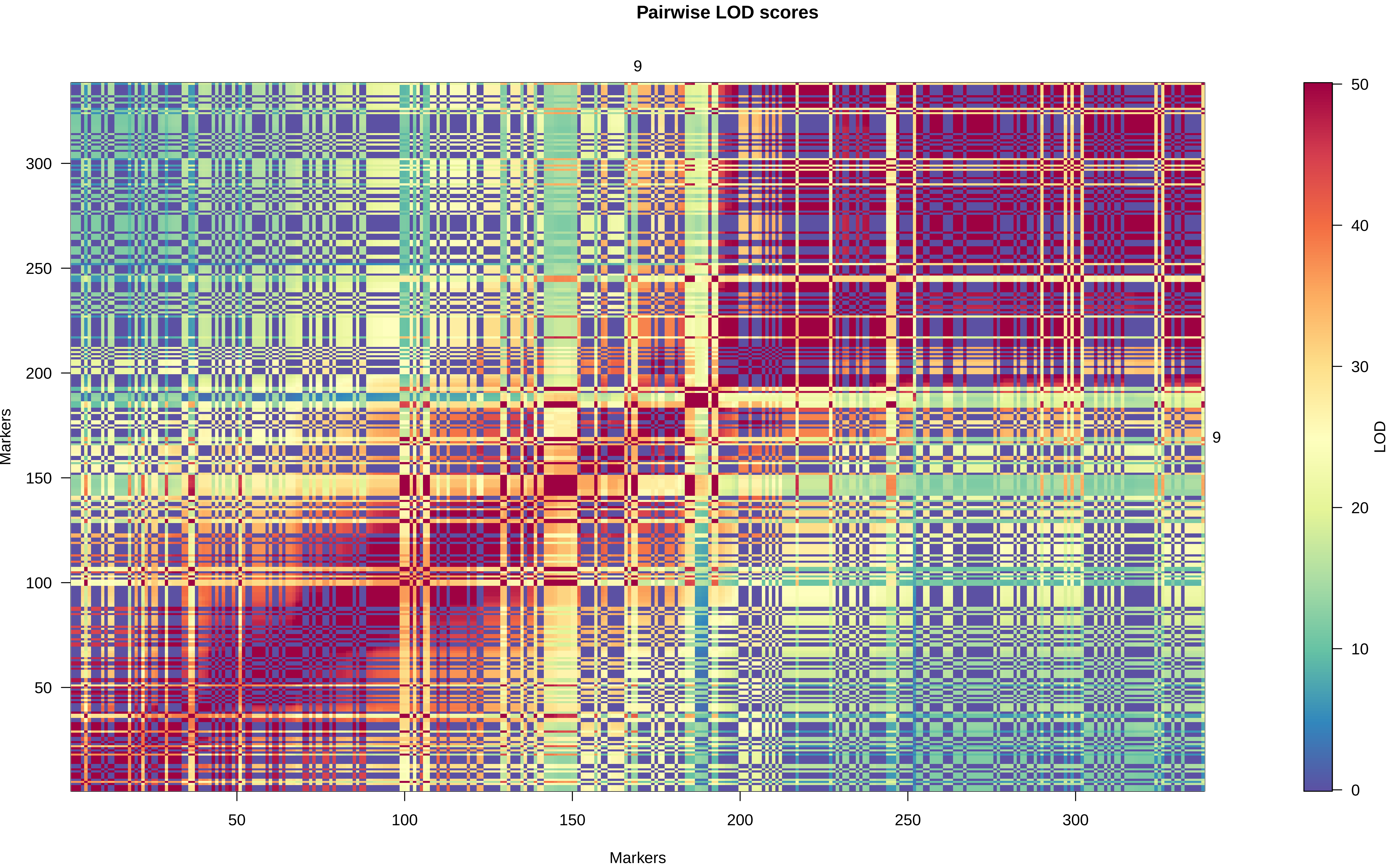

Pairwise LOD scores
9
300
250
200
150
100
50
50
100
150
200
250
300
Markers
Markers
9
50
40
30
20
10
0
LOD

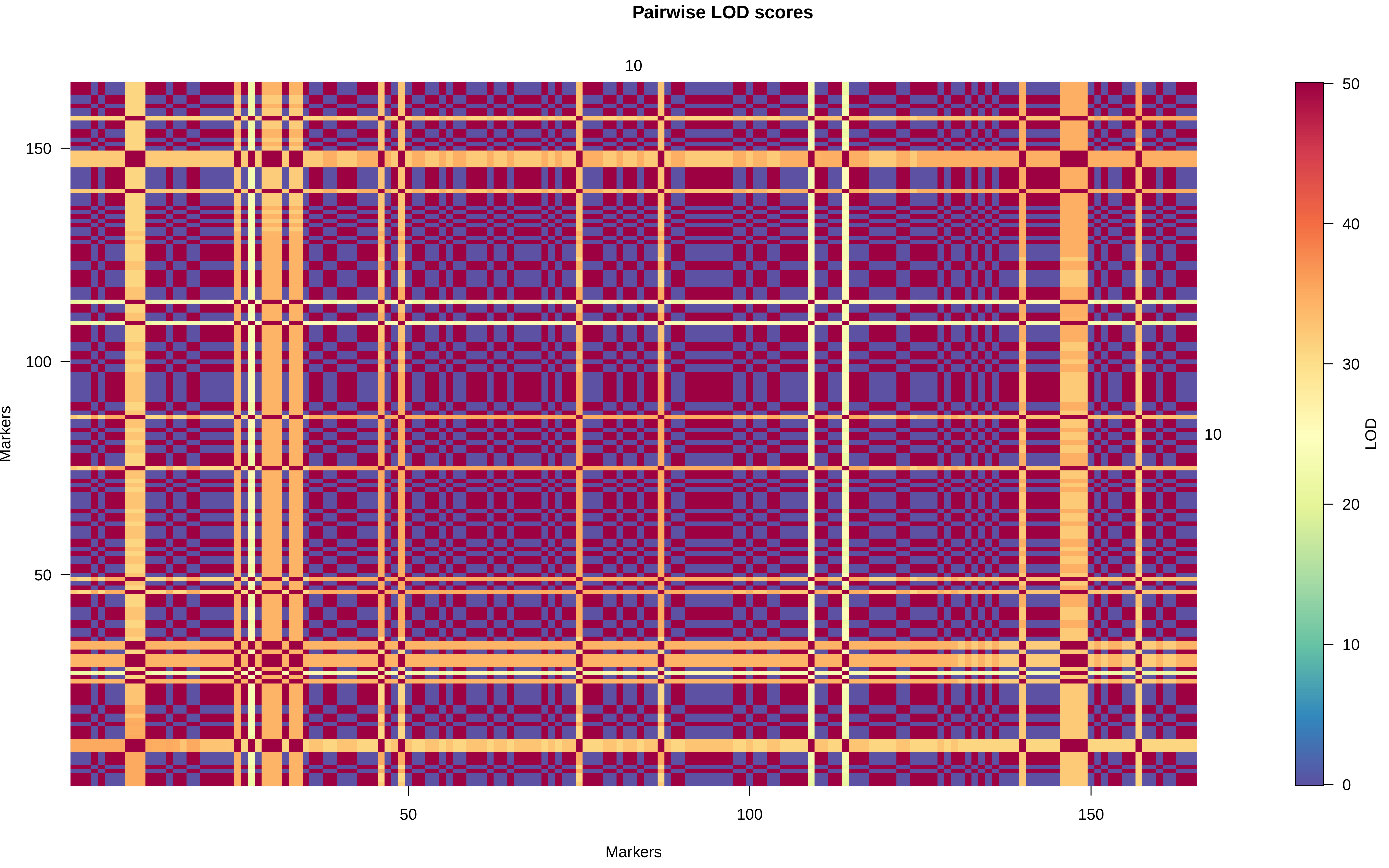

Pairwise LOD scores
10
150
100
50
Markers
10
50
100
150
Markers
50
40
30
20
10
0
LOD

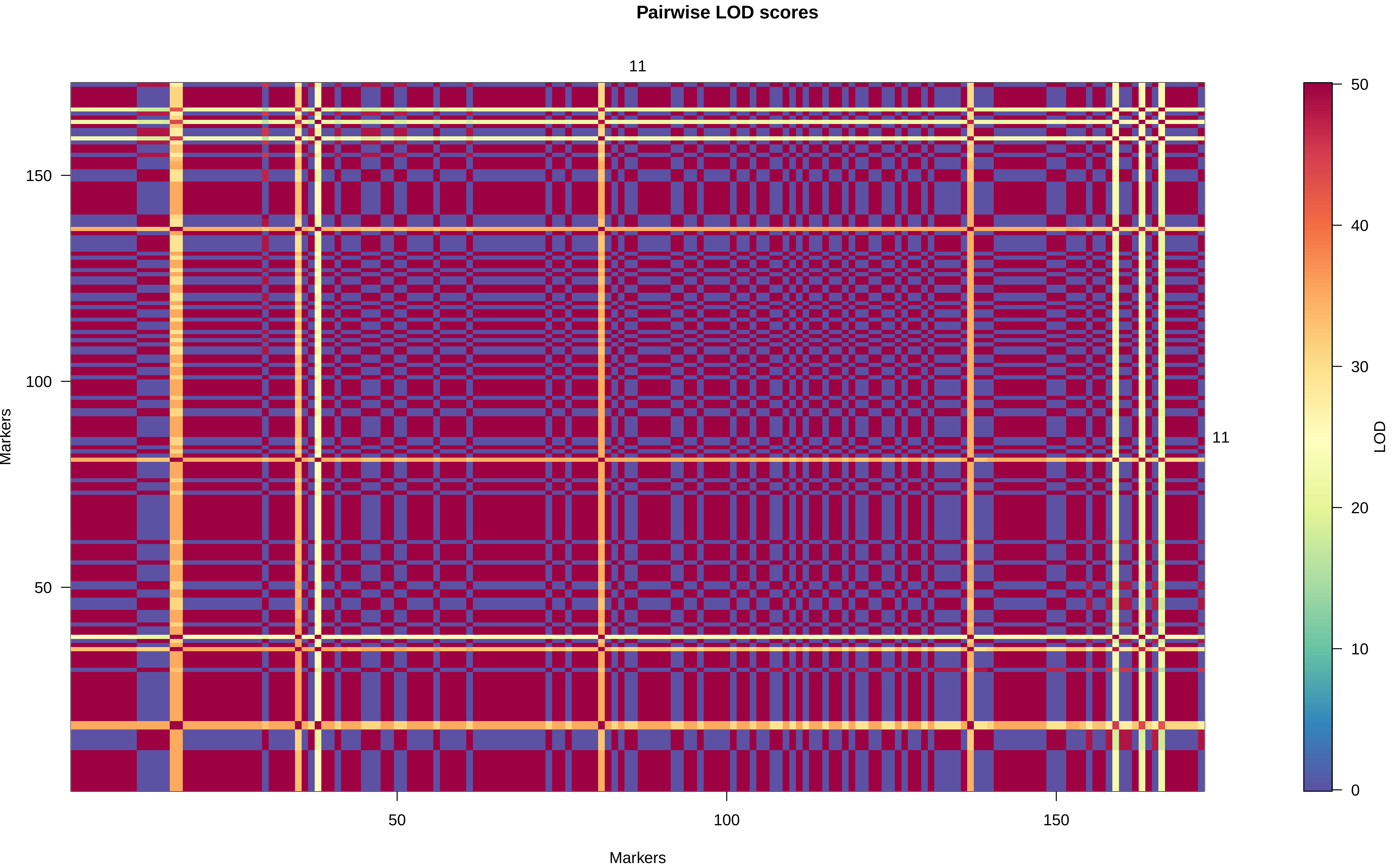

Pairwise LOD scores
11
11
150
100
50
Markers
50
100
150
Markers
50
40
30
20
10
0
LOD

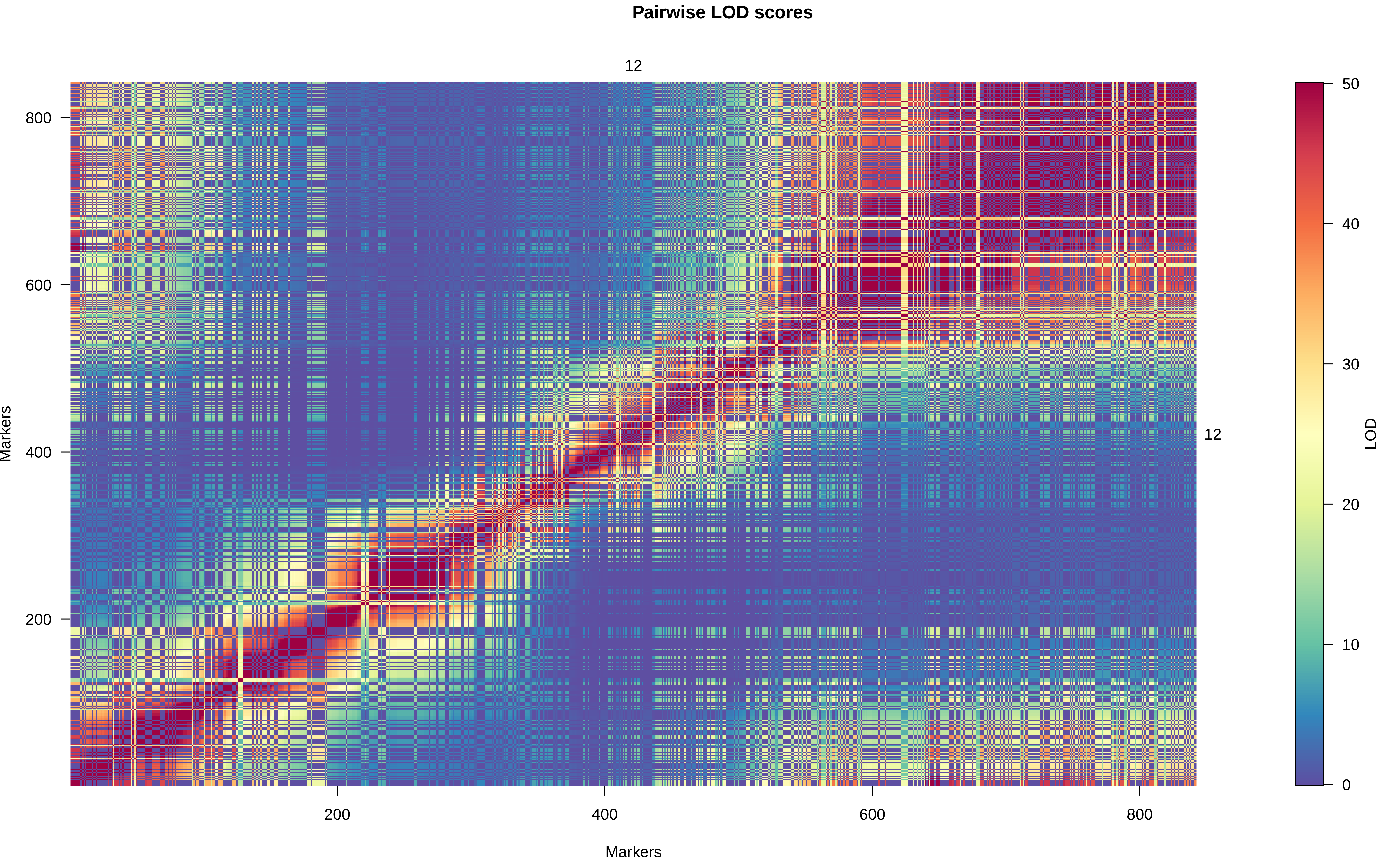
Pairwise LOD scores
12
12
Markers
Markers
200
400
600
800
200
400
600
800
LOD
0
10
20
30
40
50

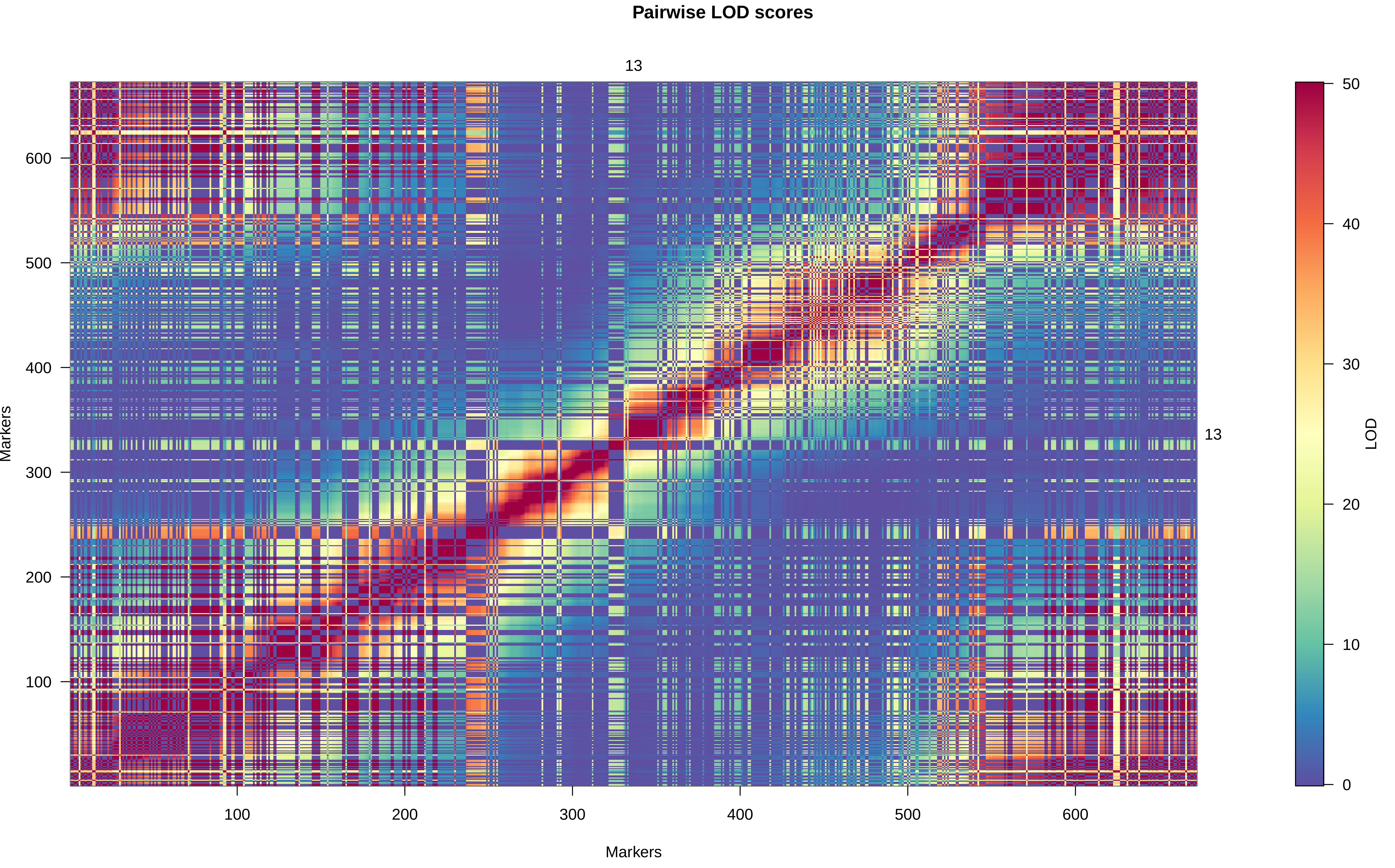
Pairwise LOD scores
13
13
Markers
Markers
LOD
100
200
300
400
500
600
0
10
20
30
40
50

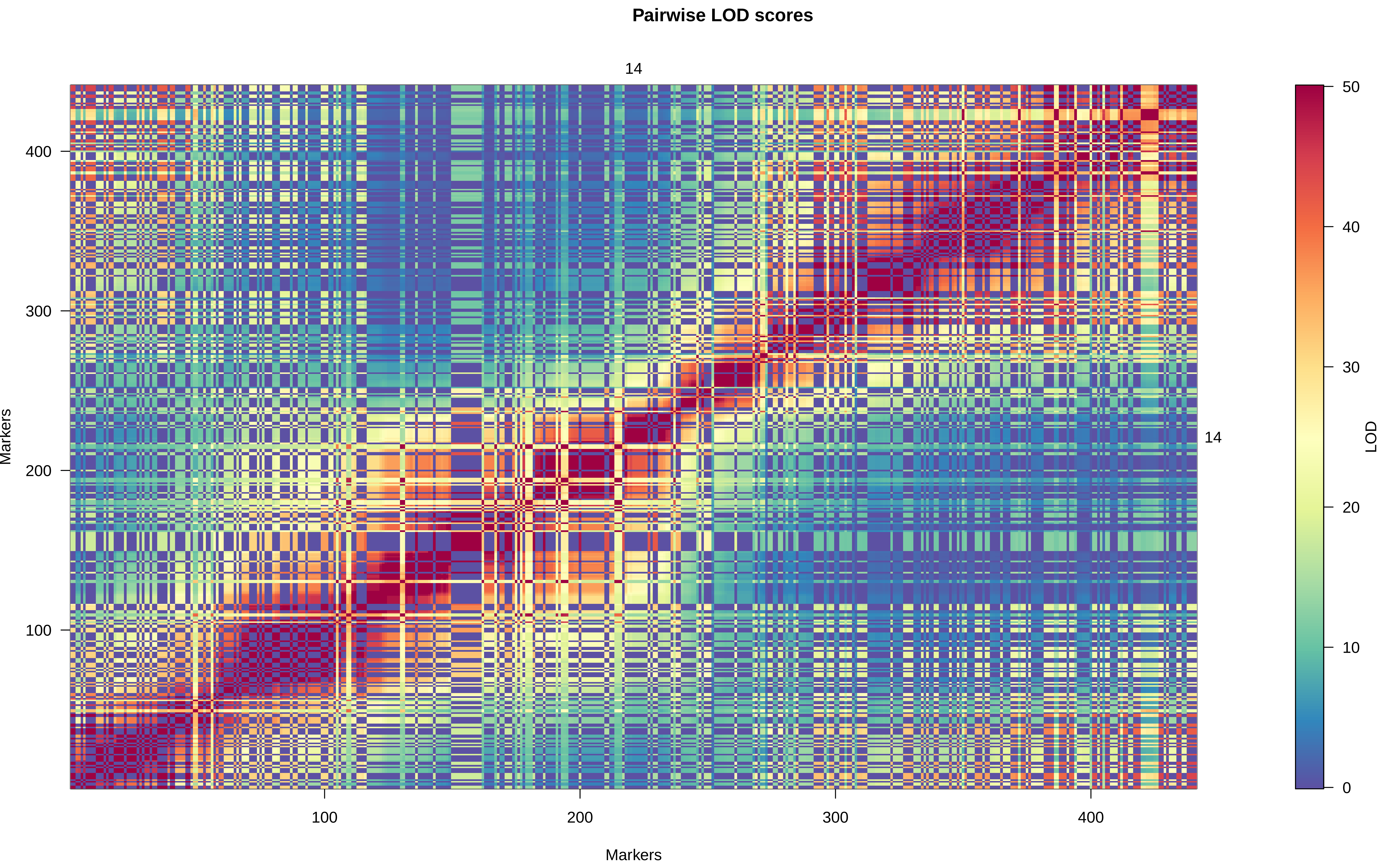

Pairwise LOD scores
14
14
400
300
200
100
100
200
300
400
Markers
Markers
LOD
50
40
30
20
10
0

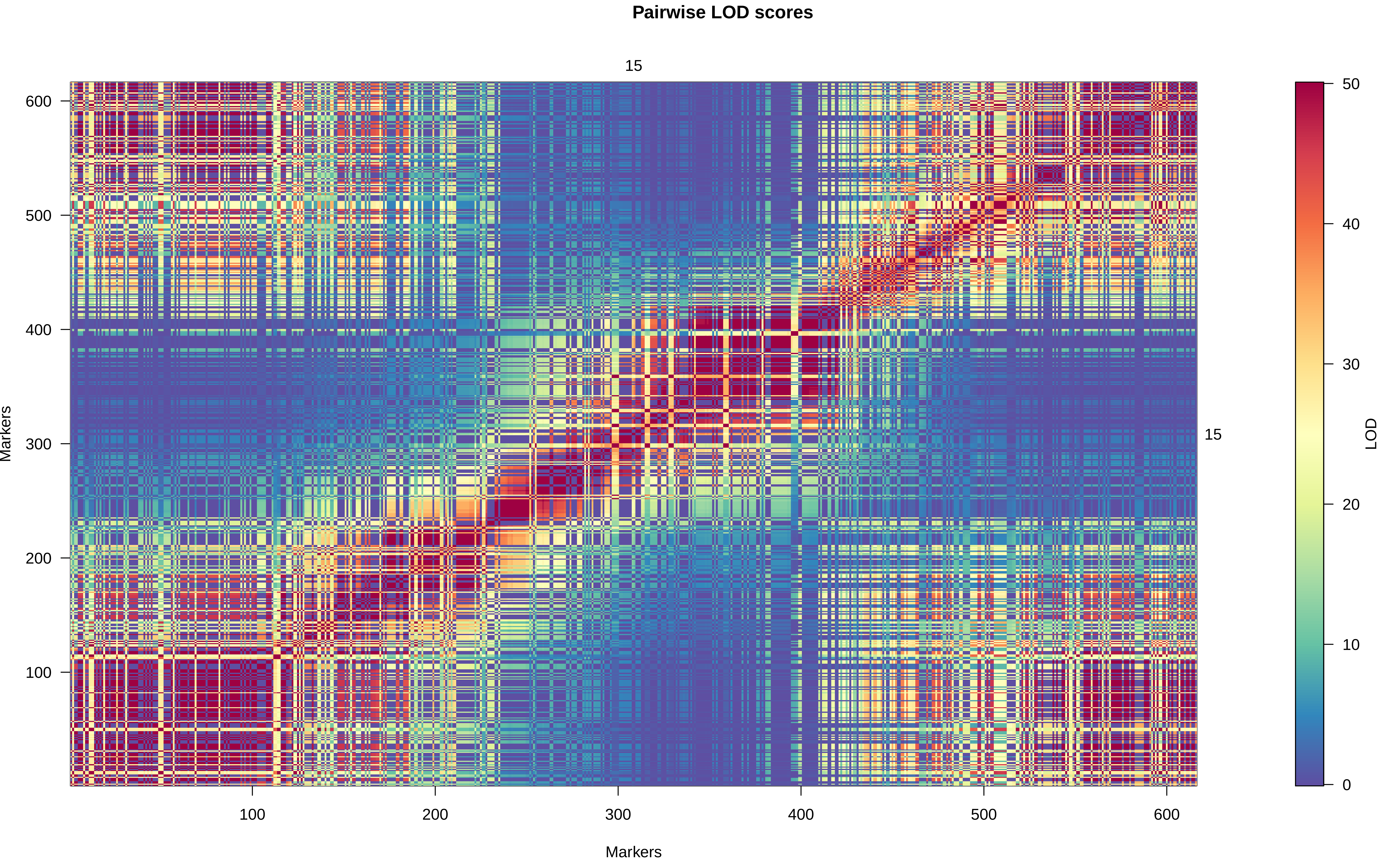
Pairwise LOD scores
15
15
Markers
Markers
100
200
300
400
500
600
LOD
0
10
20
30
40
50

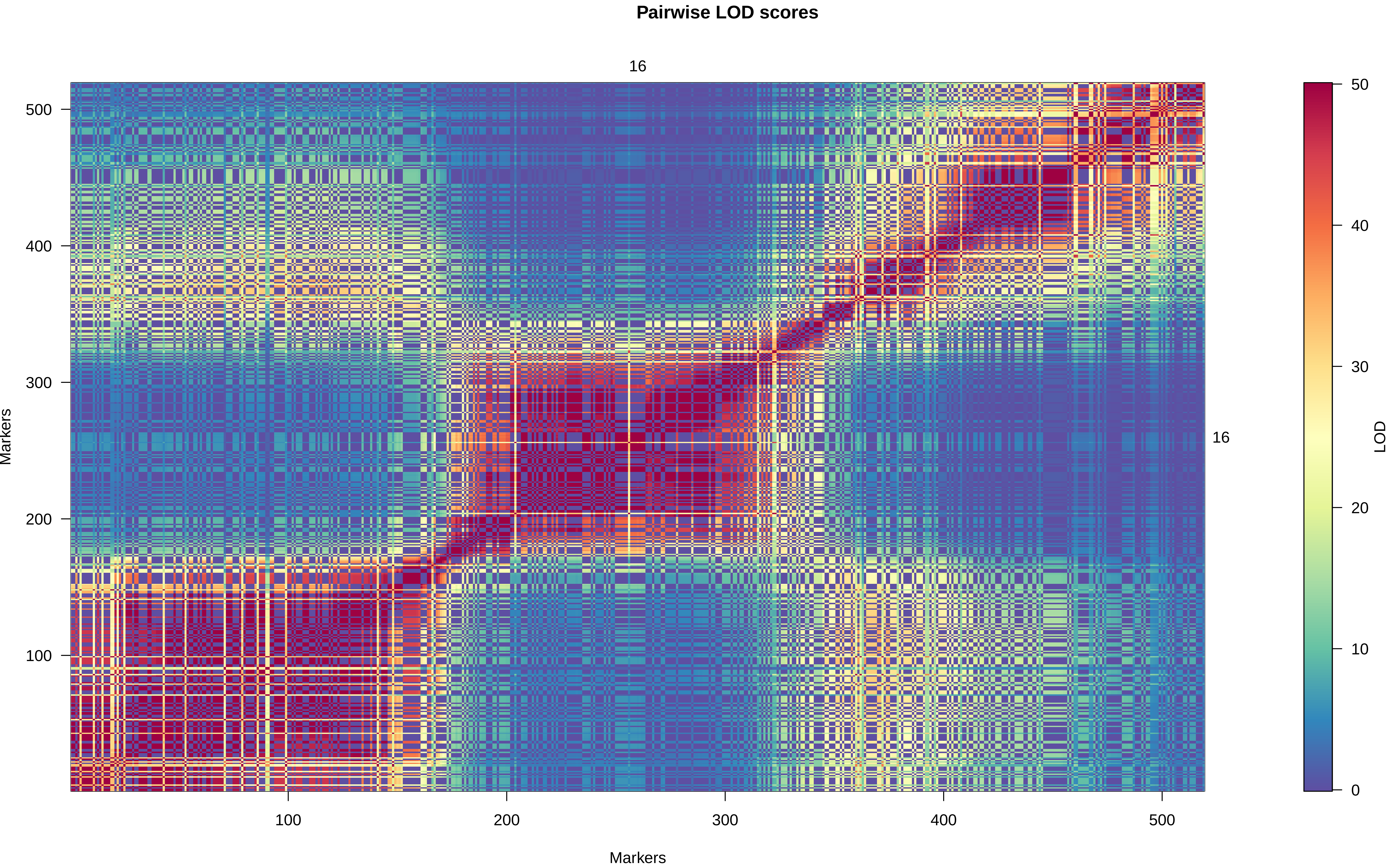

Pairwise LOD scores
16
16
Markers
Markers
100
200
300
400
500
LOD
0
10
20
30
40
50

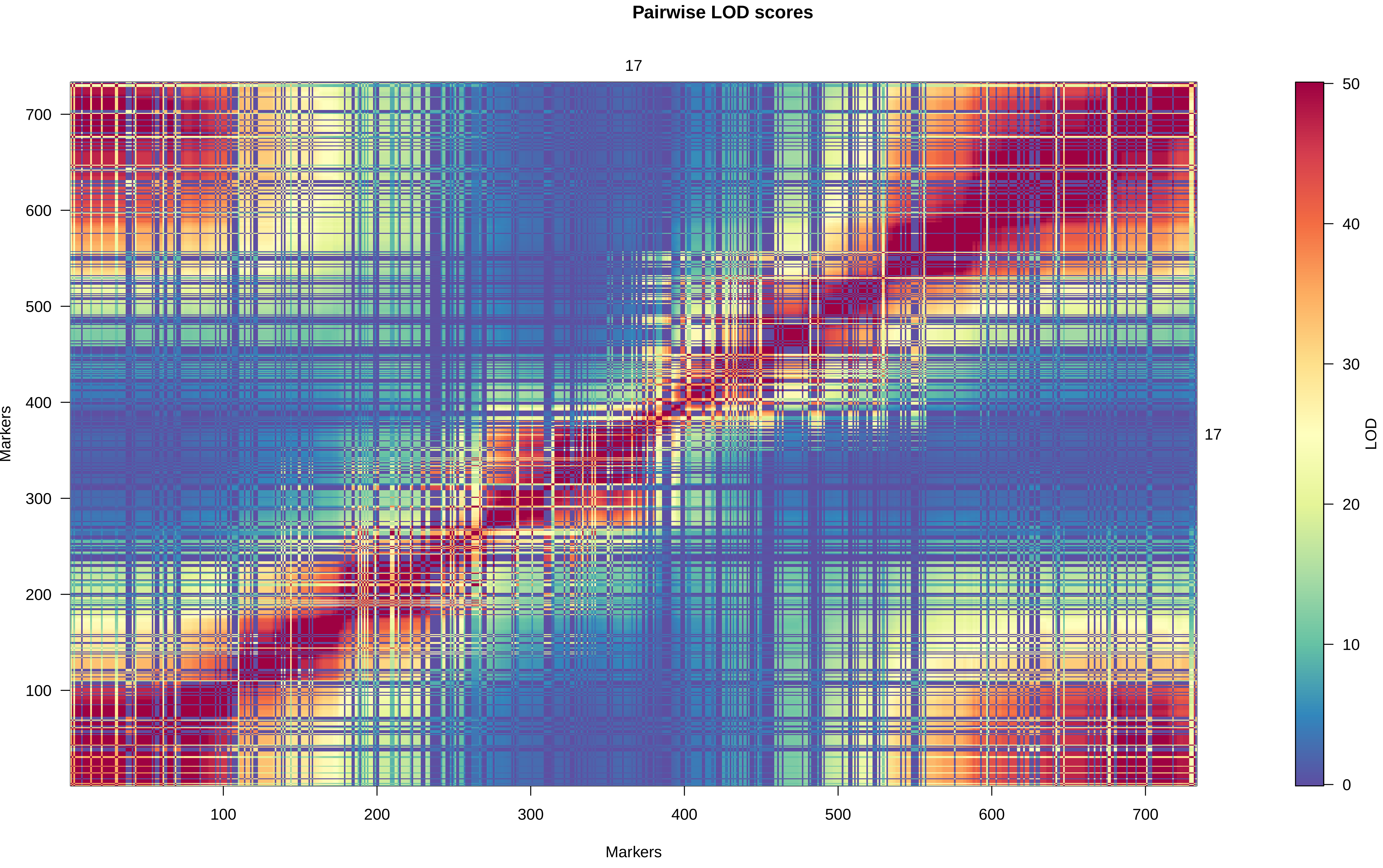

Pairwise LOD scores
17
17
Markers
Markers
100
200
300
400
500
600
700
LOD
0
10
20
30
40
50

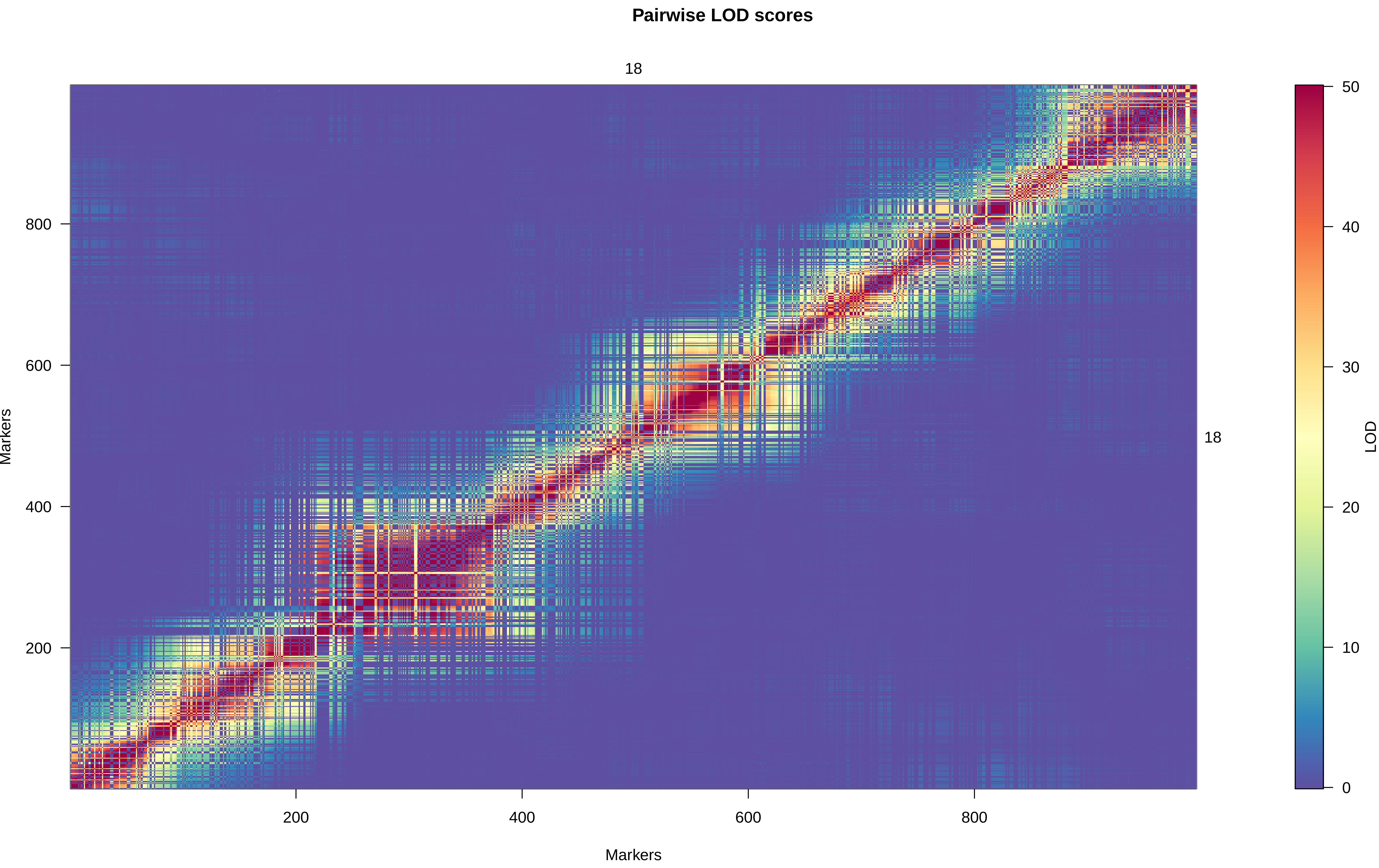
Pairwise LOD scores
18
18
Markers
Markers
800
600
400
200
200
400
600
800
LOD
50
40
30
20
10
0

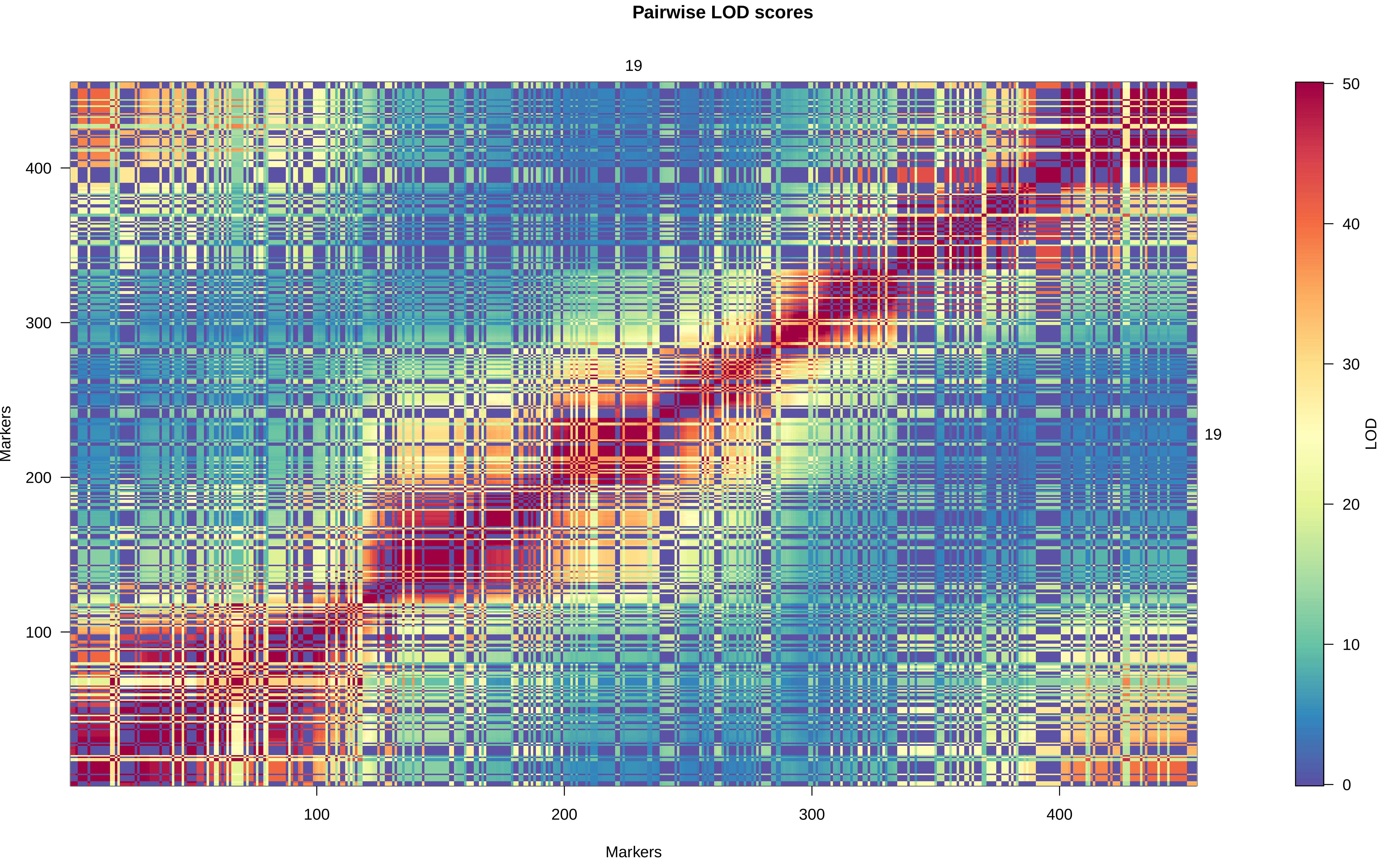
Pairwise LOD scores
19
19
400
300
200
100
Markers
100
200
300
400
Markers
50
40
30
20
10
0
LOD

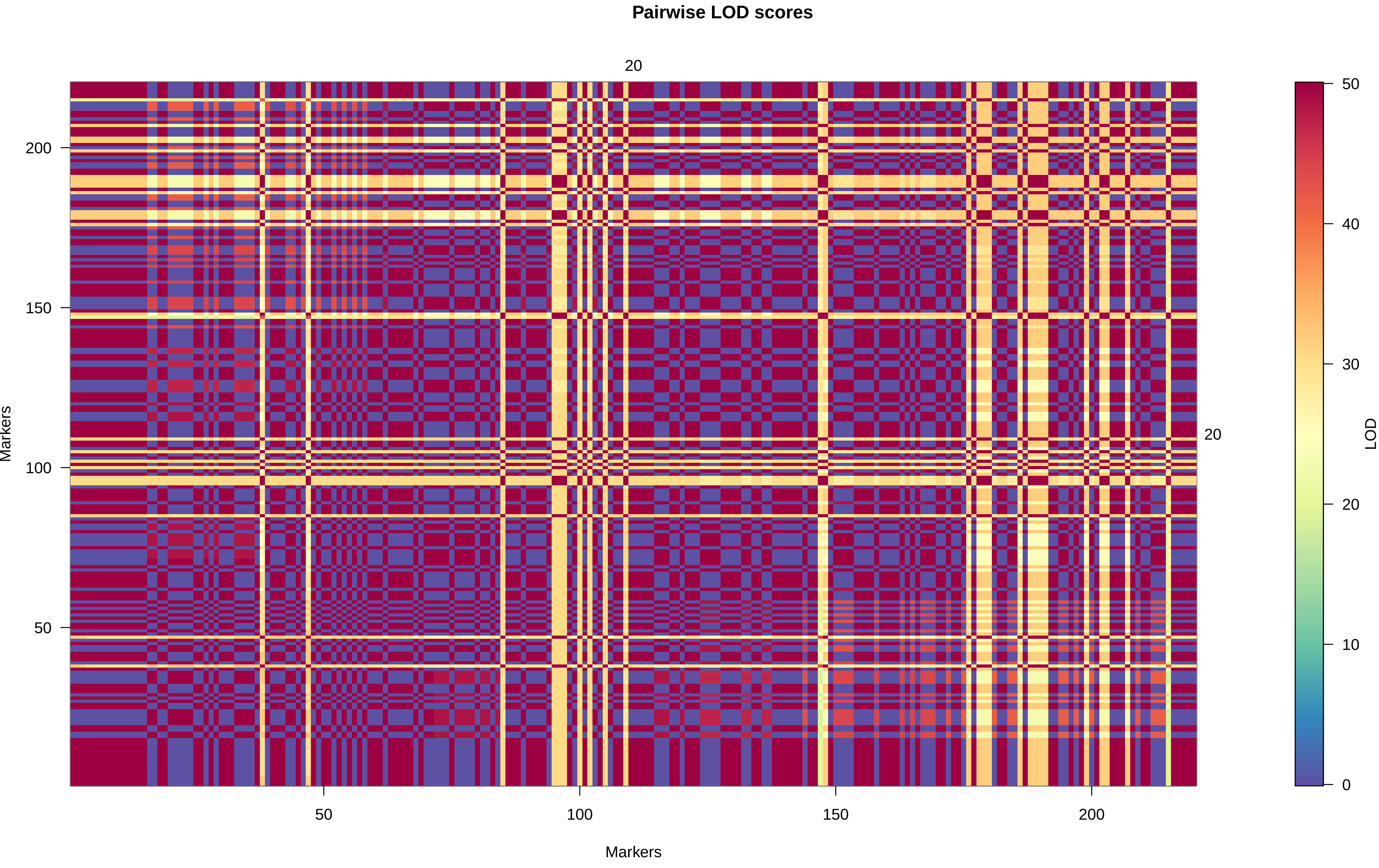
Pairwise LOD scores
20
20
Markers
Markers
200
150
100
50
50
100
150
200
LOD
50
40
30
20
10
0
