# Supplementary figures and images for: Construction of a high-density genetic map and QTL mapping of leaf traits and plant growth in an interspecific F1 population of Catalpa bungei × Catalpa duclouxii Dode
Source: BMC Plant Biol. 2019 Dec 30;19:596. doi: 10.1186/s12870-019-2207-y (PMC6937828; doi:10.1186/s12870-019-2207-y)

# LeafArea

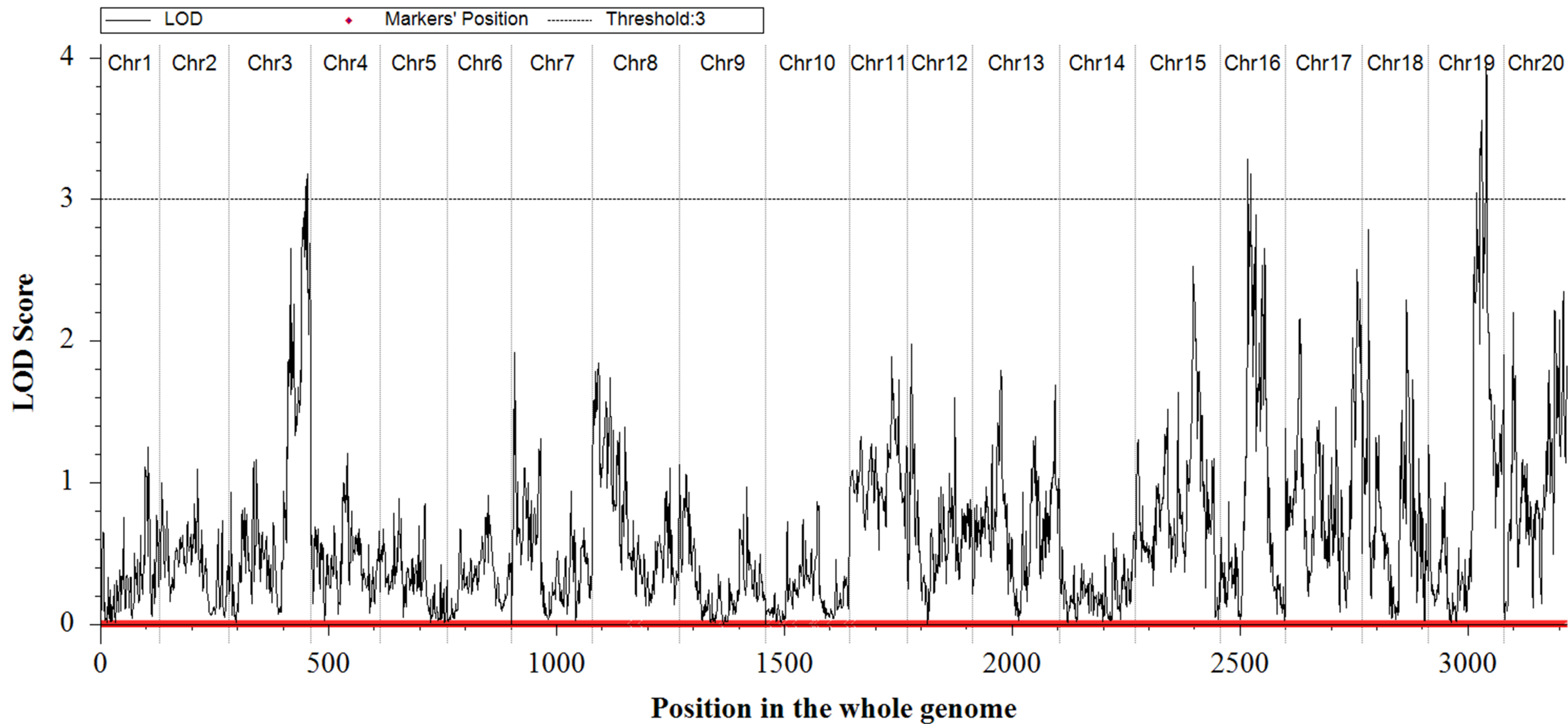

# LeafLength

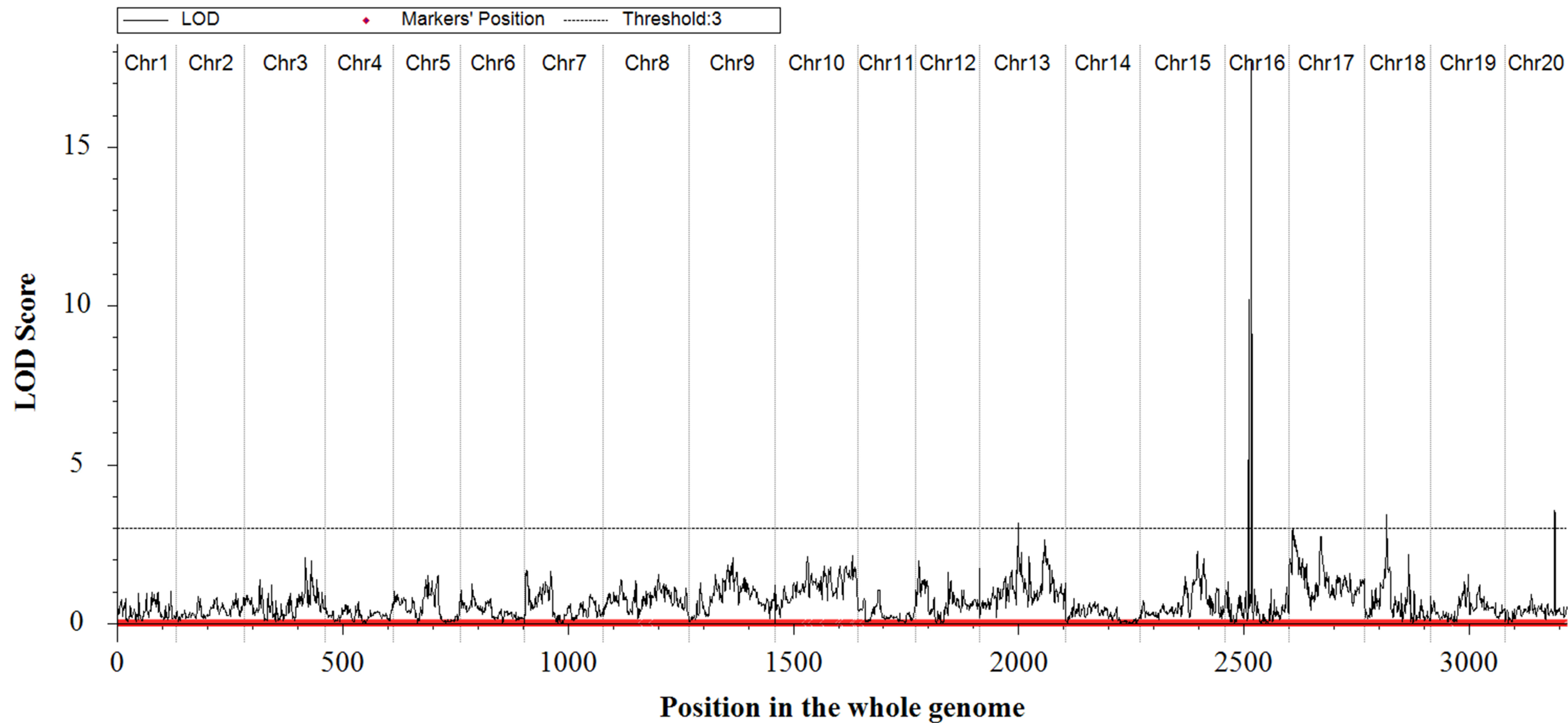

# LeafPerimeter

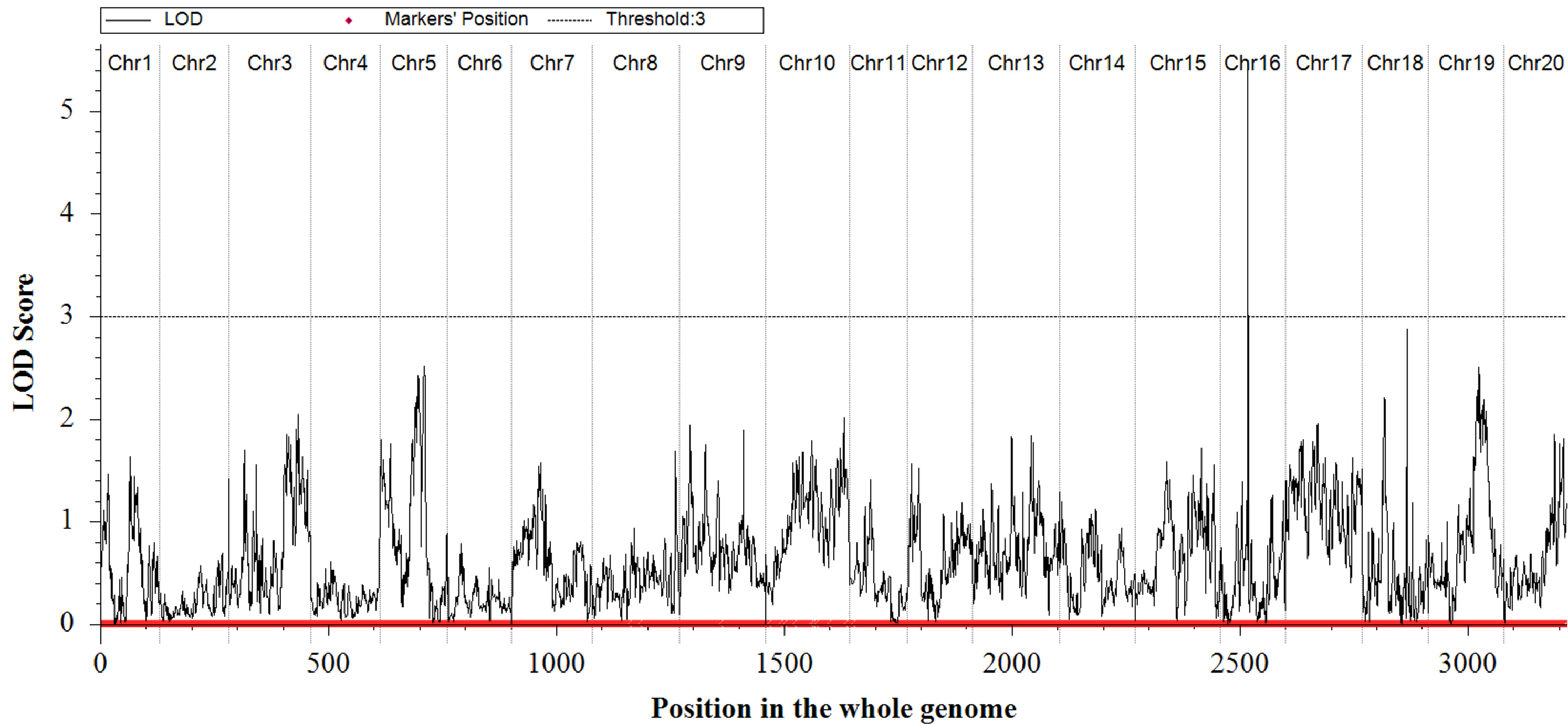

# LeafWidth

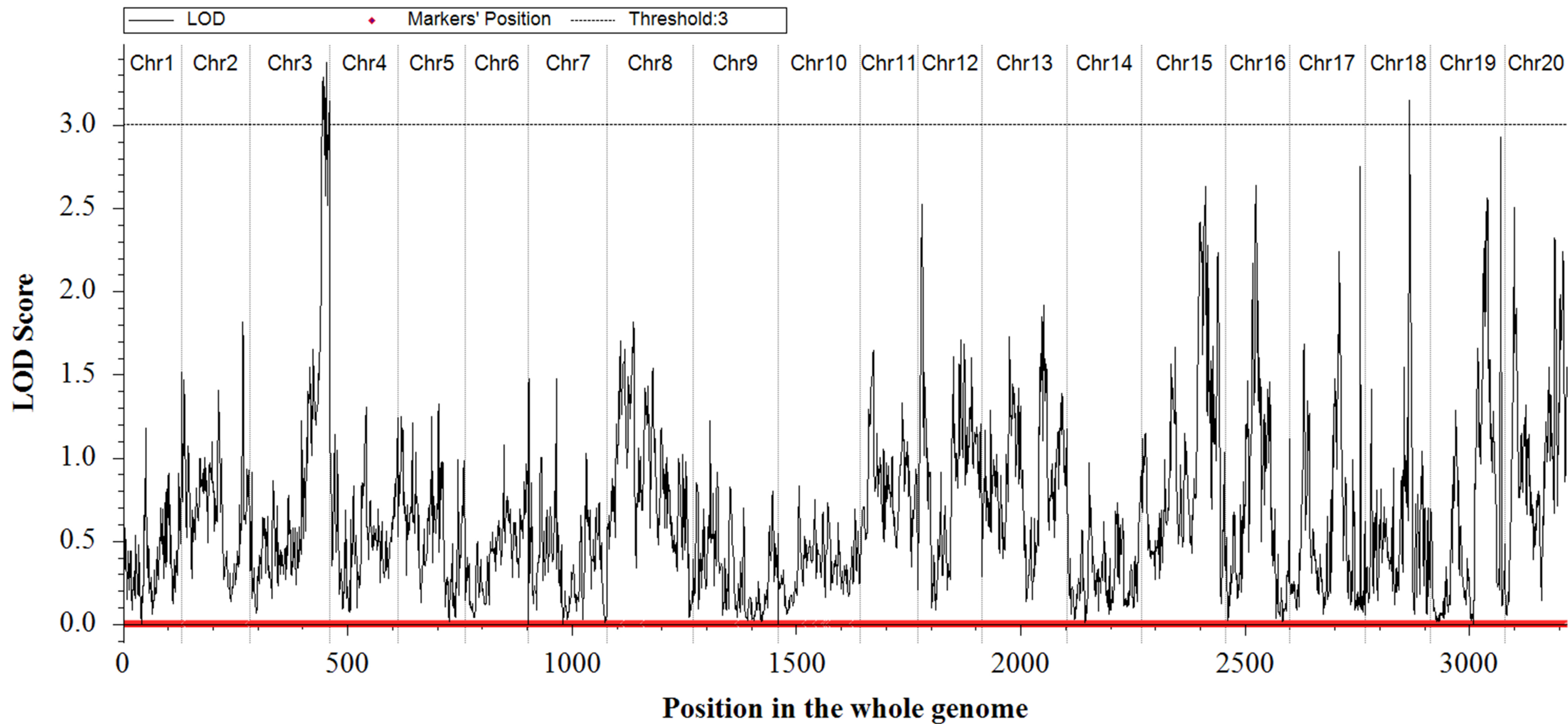

# L/W ratio

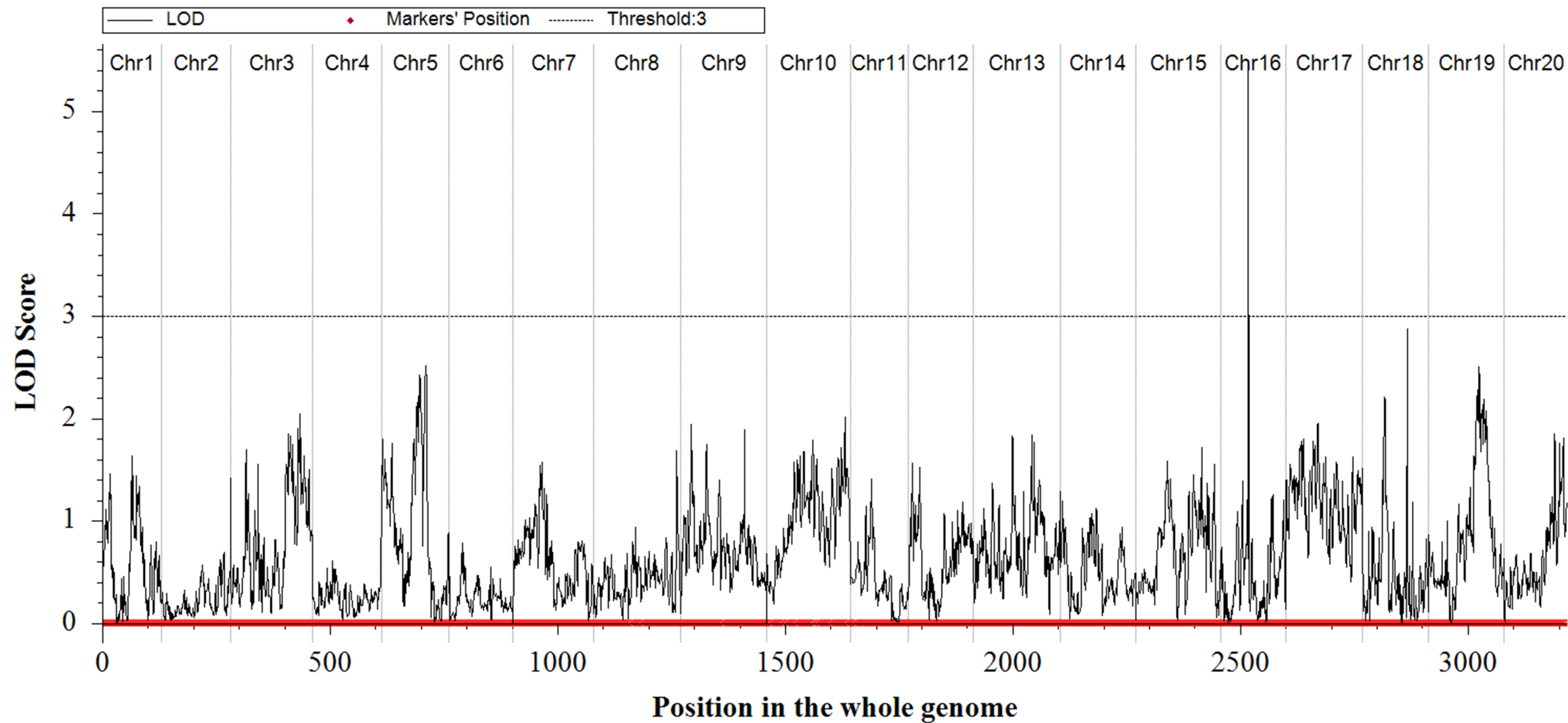

# PetioleLength

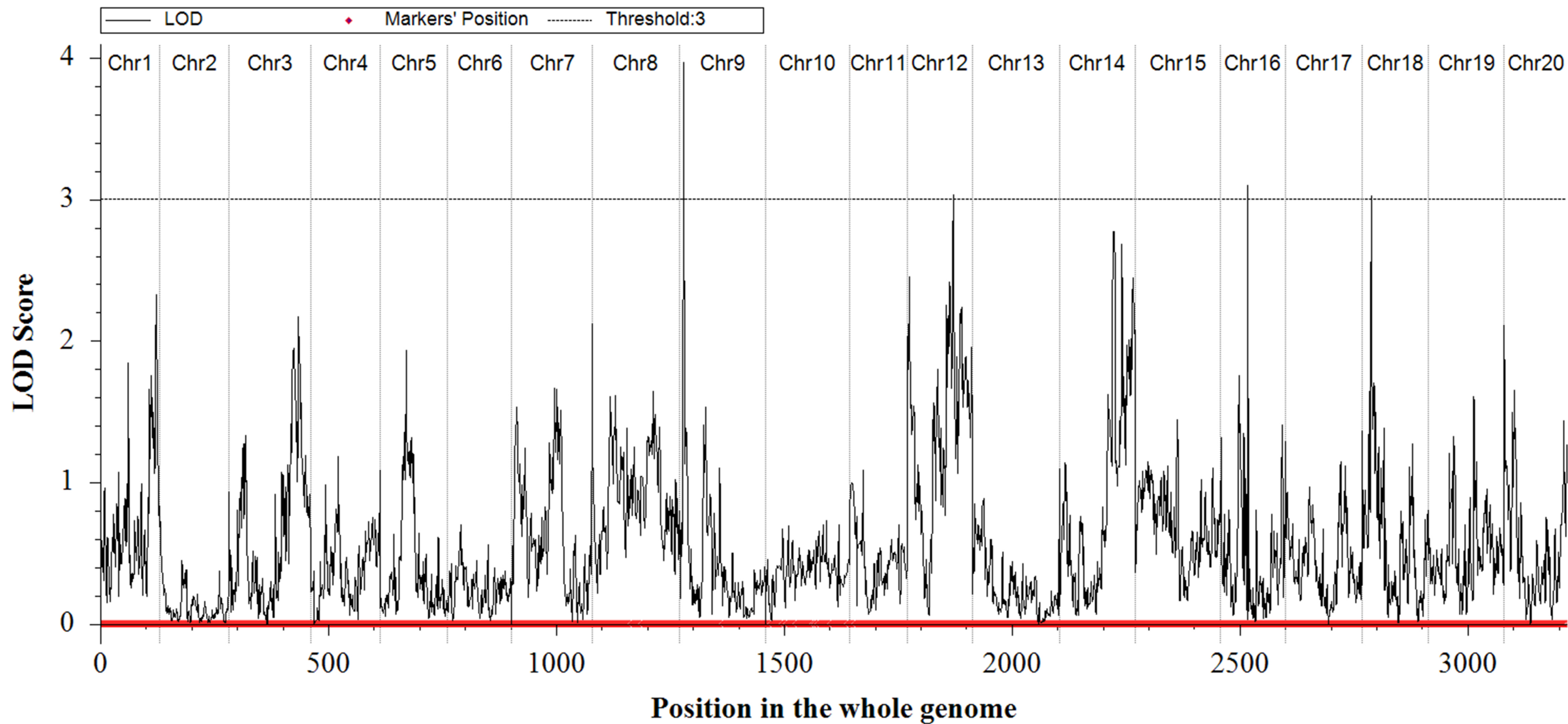

# SPAD

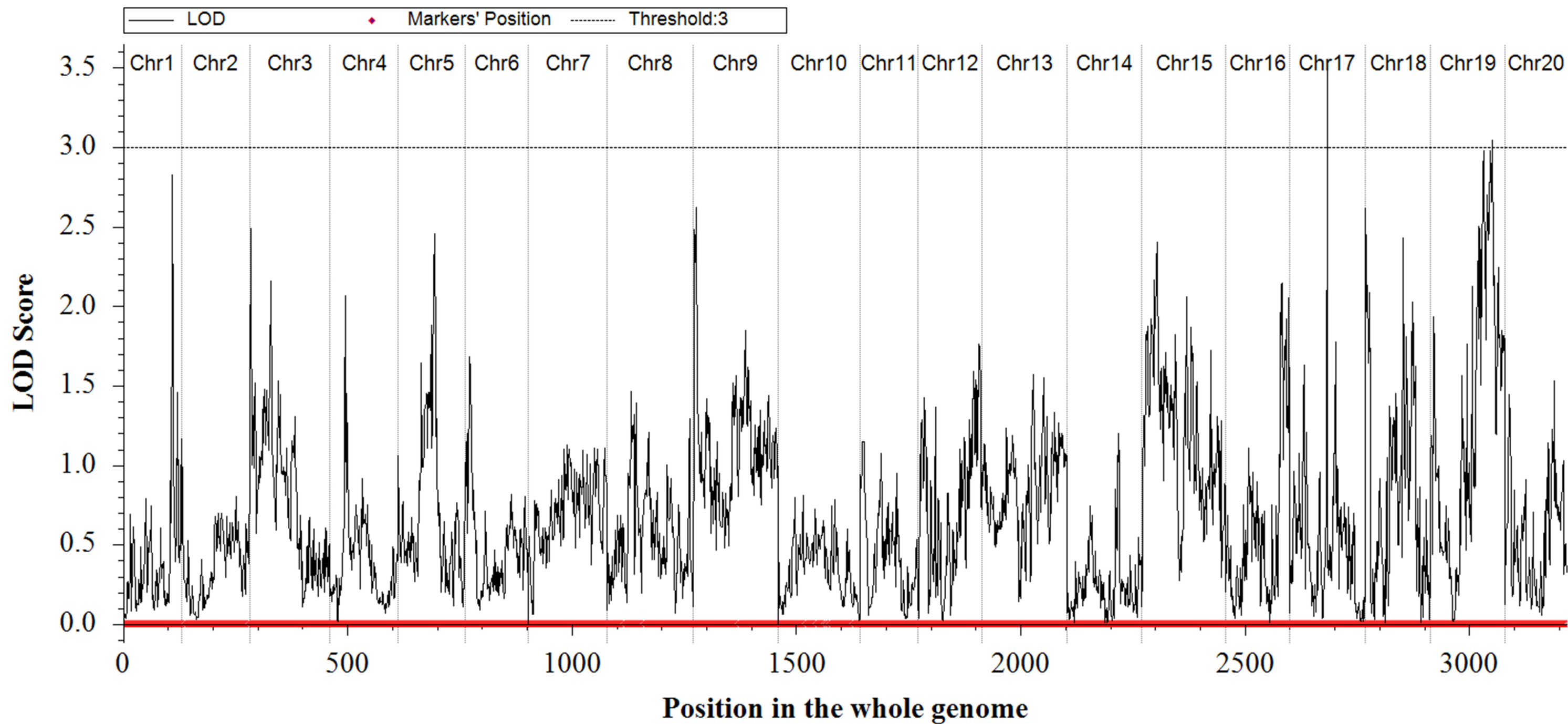

Supplement: Supplementary file 8 — Additional file 8: Figure S3. QTL analysis of the seven leaf traits using the ICIM method in GACD. The x-axis indicates the map position (cM) in the 20 LGs, while the y-axis represents the LOD score. The horizontal line in the chart represents the LOD threshold. [file 12870_2019_2207_MOESM8_ESM.pdf]

# PlantHeight(10.10)

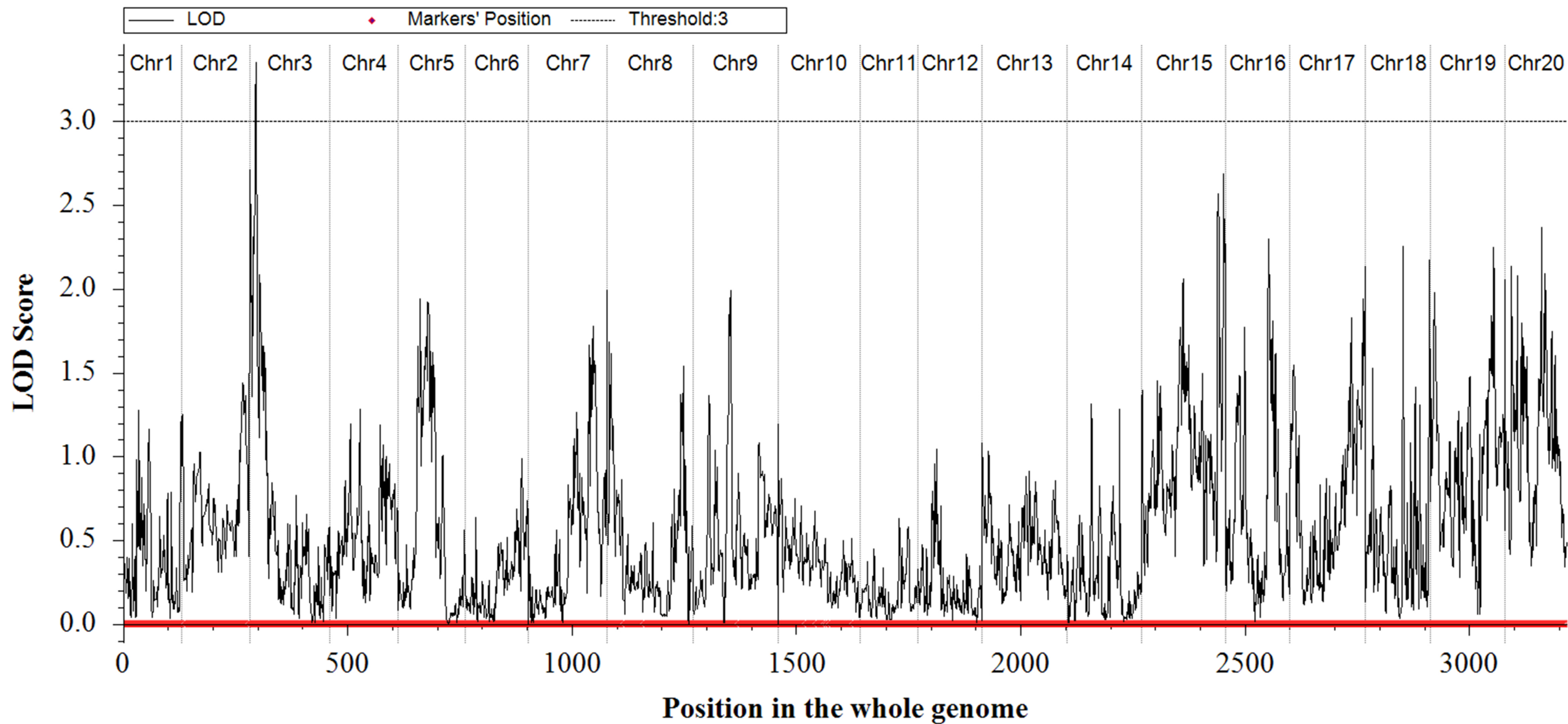

# PlantHeight(6.30)

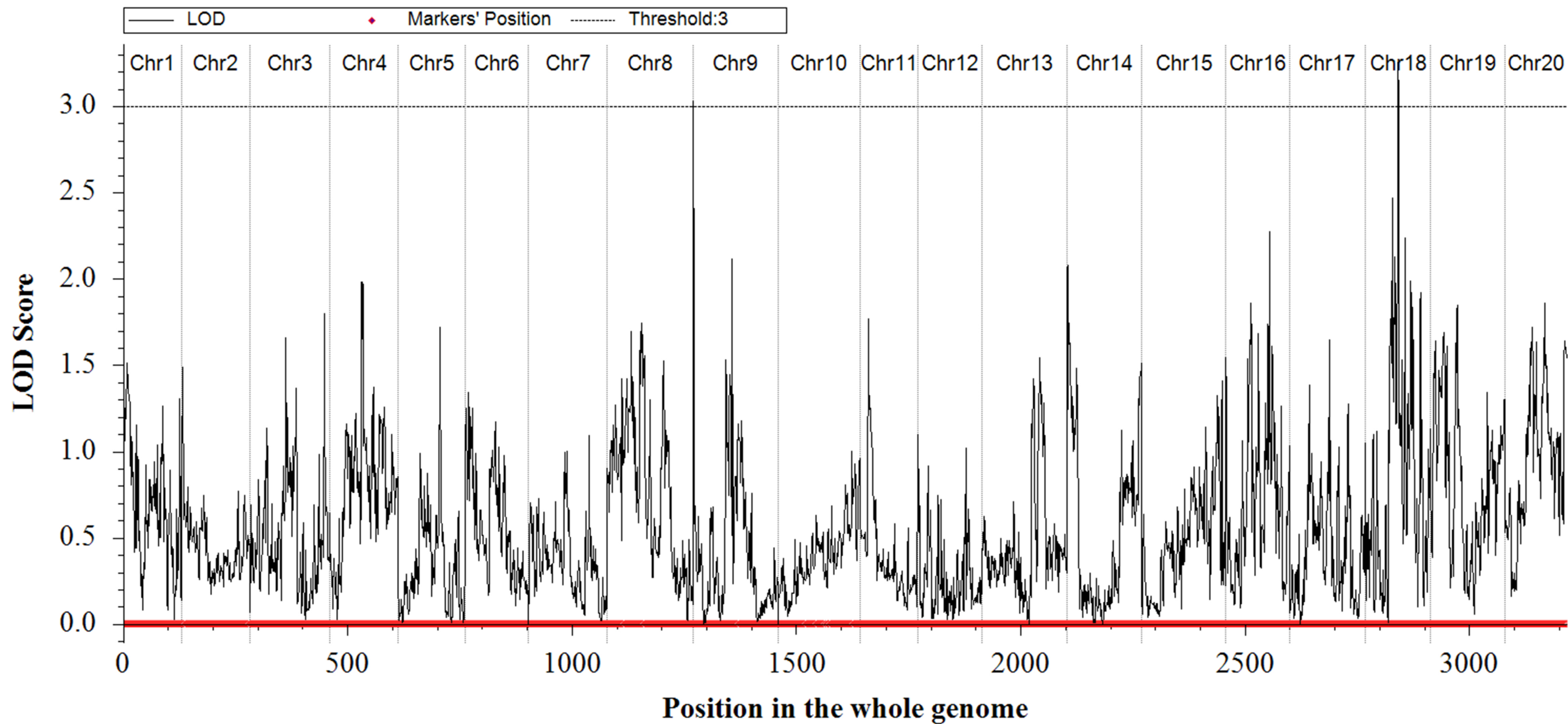

# PlantHeight(7.15)

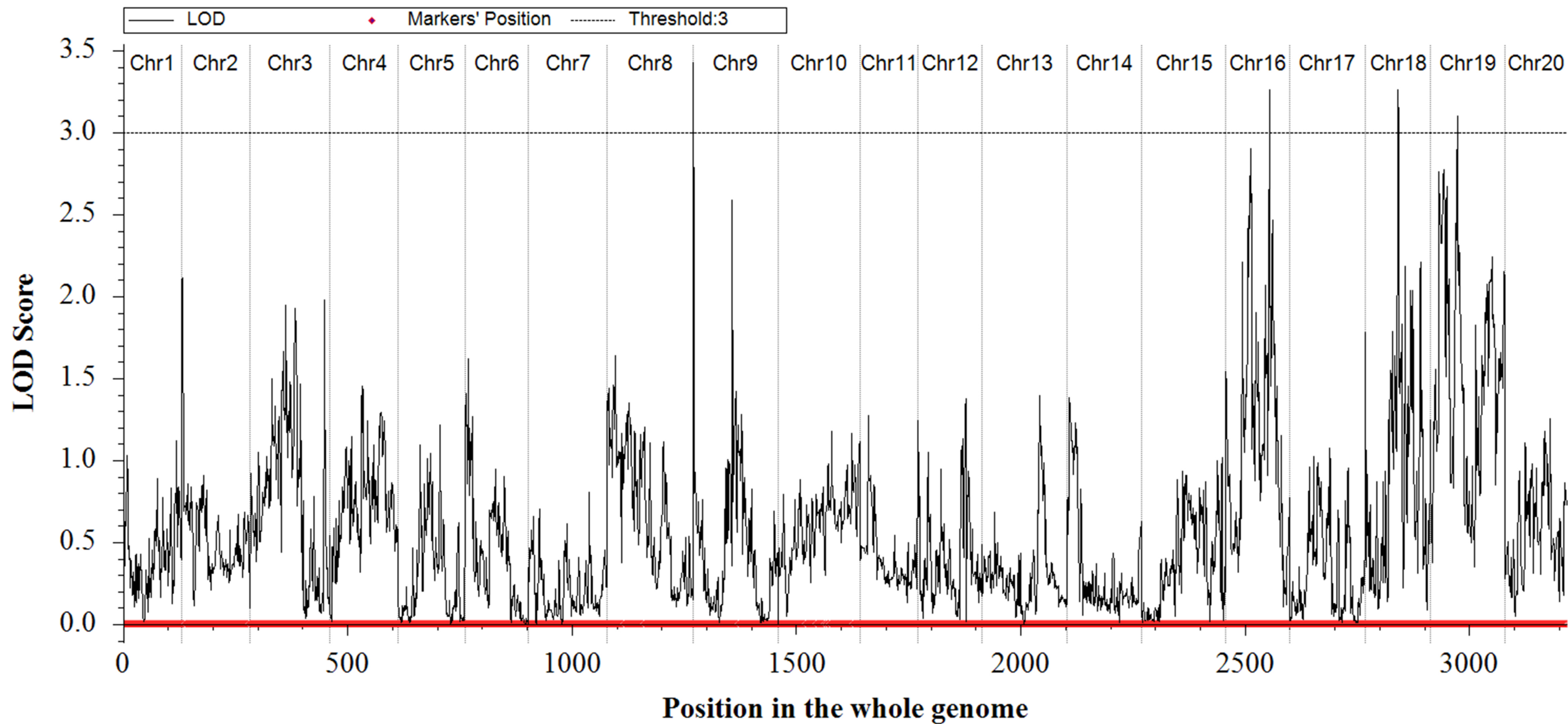

# PlantHeight(7.31)

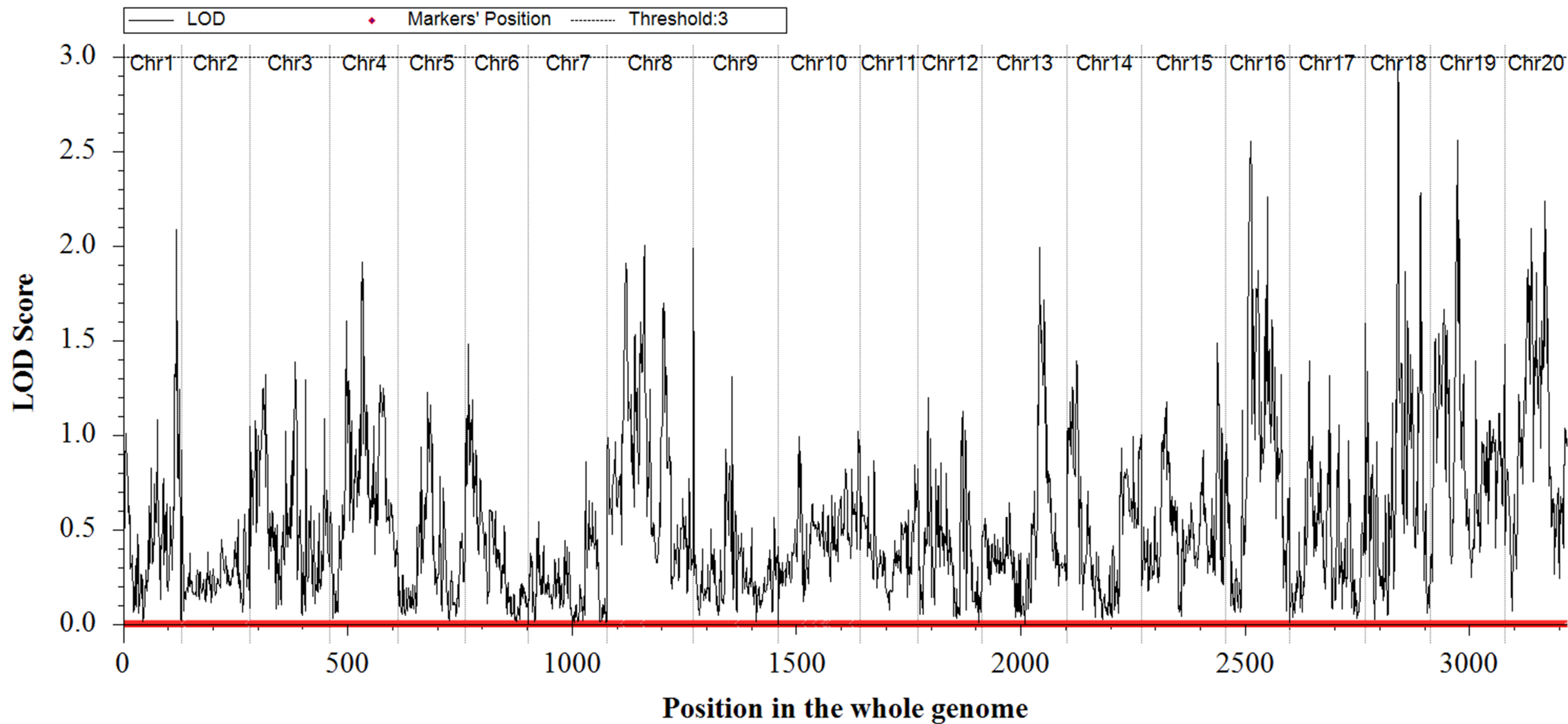

# PlantHeight(8.15)

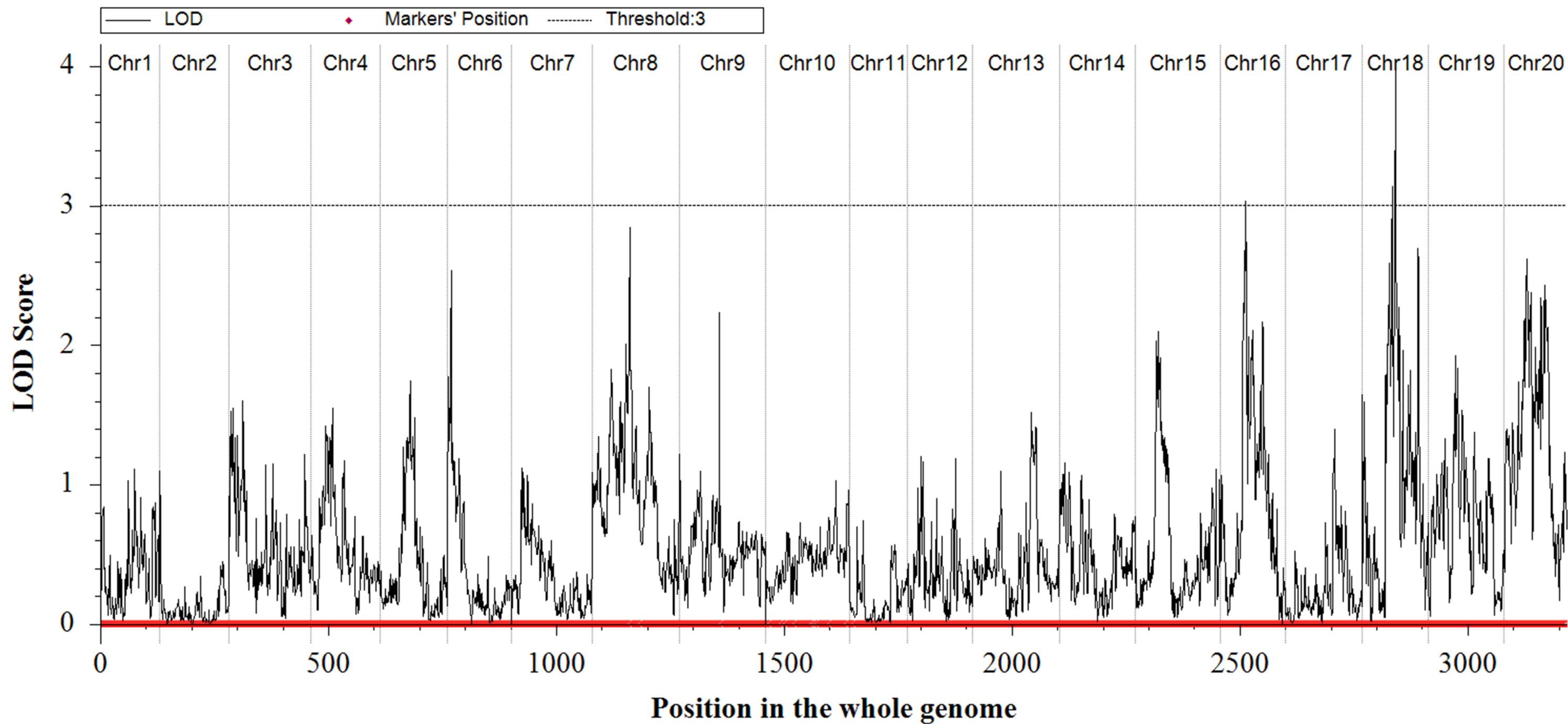

# PlantHeight(8.15)

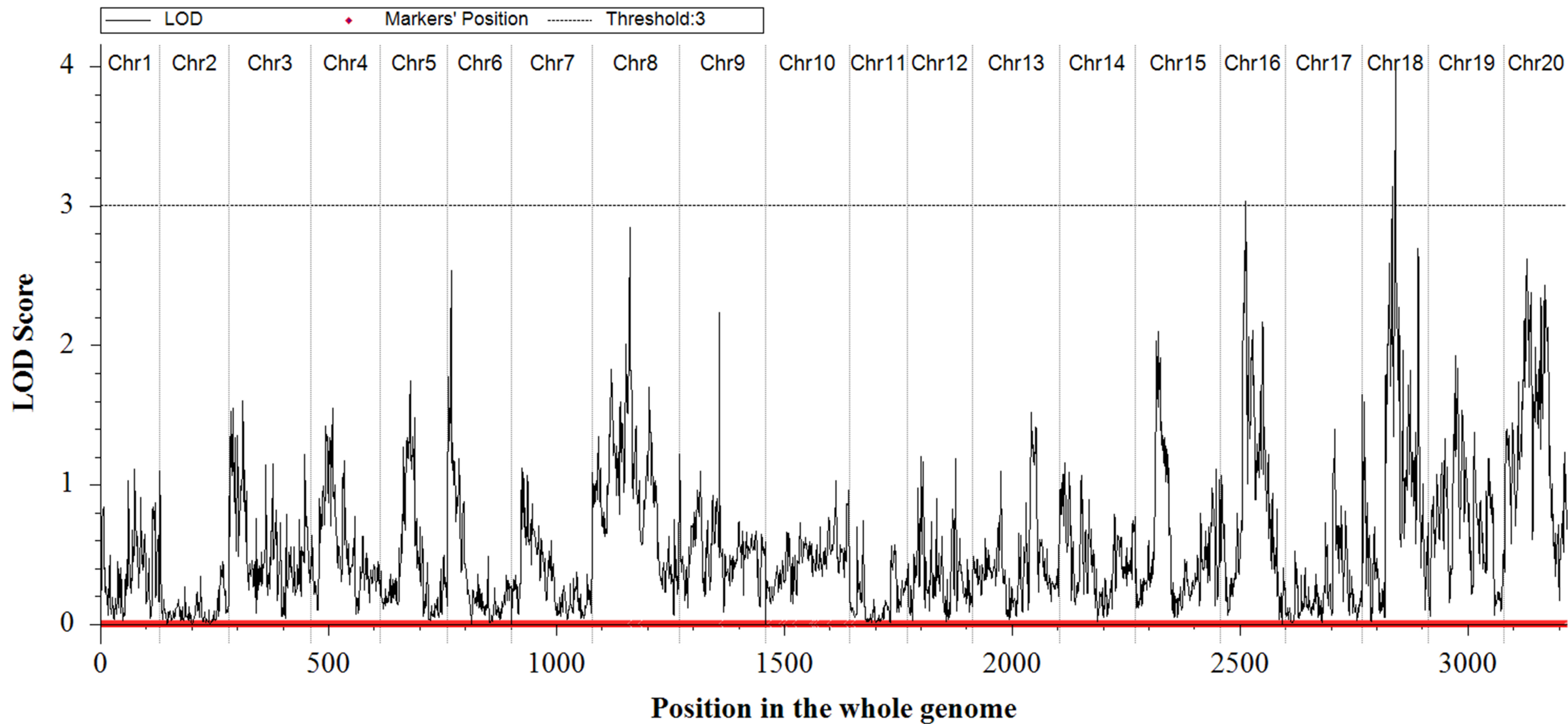

Supplement: Supplementary file 9 — Additional file 9: Figure S4. QTL analysis of the plant growth traits at different time points using the ICIM method in GACD. The x-axis indicates the map position (cM) in the 20 LGs, while the y-axis represents the LOD score. The horizontal line in the chart is the LOD threshold. [file 12870_2019_2207_MOESM9_ESM.pdf]

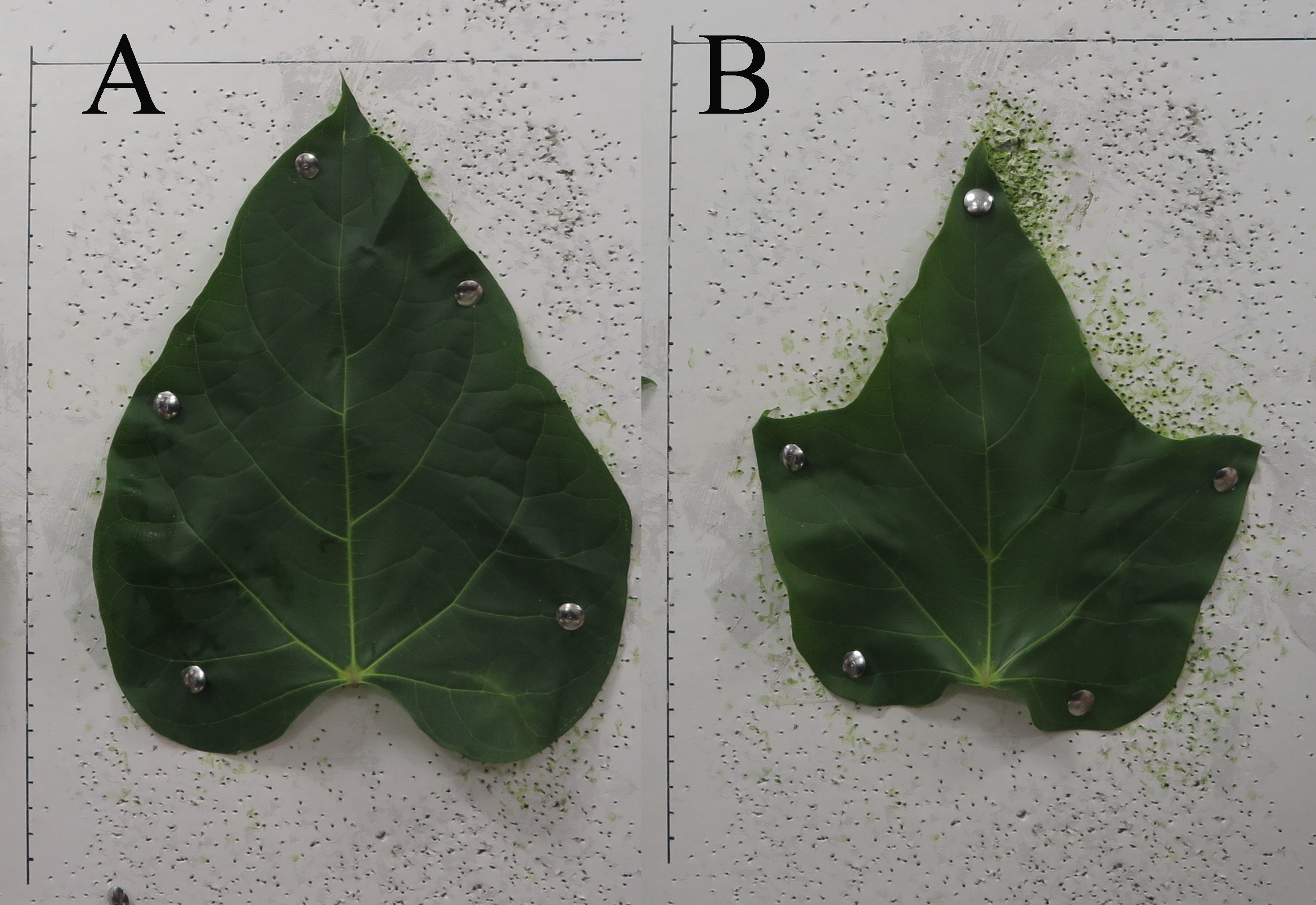

Supplement: Supplementary file 13 — Additional file 13: Figure S5. The leaf of “7080” (A) and “16-PJ-3” (B). [file 12870_2019_2207_MOESM13_ESM.jpg]

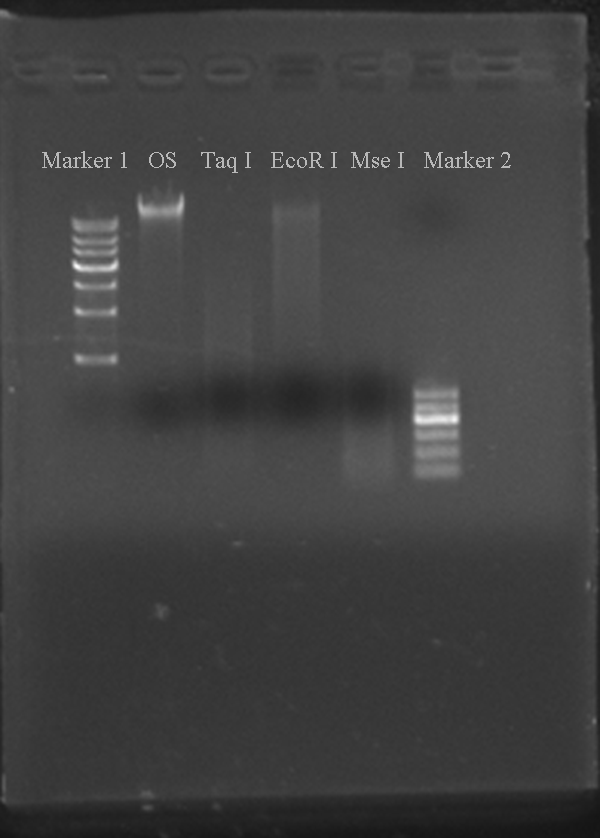

Supplement: Supplementary file 14 — Additional file 14: Figure S6. The gel electrophoresis of DNA digestion pre-experiment using TaqI, EcoRI and MseI. The “OS” stands for original DNA sample without enzyme treatment; Marker 1 and marker 2 are 1Kb DNA Ladder (TransGen Biotech, Beijing, China) and Marker I (Tiangen, Beijing, China), respectively. [file 12870_2019_2207_MOESM14_ESM.tif]
